# Acute capillary blood DNA methylation responses to swimming exercise in high‐performance male and female swimmers

**DOI:** 10.1113/EP093387

**Published:** 2026-06-12

**Authors:** C. D. Goldsmith, N. G. Lawler, D. B. Pyne, M. Kozlovskaia, K. McGibbon, L. J. G. Mitchell, A. D. Govus

**Affiliations:** ^1^ Translational Health Research Institute Western Sydney University Parramatta New South Wales Australia; ^2^ School of Science Western Sydney University Parramatta New South Wales Australia; ^3^ Australian National Phenome Centre, Health Futures Institute Murdoch University Perth Western Australia Australia; ^4^ Centre for Computational and Systems Medicine, Health Futures Institute Murdoch University Perth Western Australia Australia; ^5^ Research Institute Sport and Exercise University of Canberra Canberra Australia; ^6^ Performance Science Unit Queensland Academy of Sport Nathan Queensland Australia; ^7^ Victorian Institute of Sport Albert Park Victoria Australia; ^8^ Discipline of Sport and Exercise Science La Trobe University Bundoora Victoria Australia

**Keywords:** 5mC, biomarkers, epigenetics, phenotype, RRBS, sport

## Abstract

Epigenetic markers, particularly DNA methylation, are promising tools for monitoring athlete health and training due to their role in cellular regulation, exercise responsiveness and molecular stability. However, exercise‐induced epigenetic changes in peripheral blood in trained individuals remain unexplored. Therefore, we investigated the whole blood DNA methylation responses to swimming performed in the moderate (below critical speed) and severe (above critical speed) intensity domains using minimally invasive sampling. Ten high‐performance swimmers (5 males, 5 females; age 16–24 years) completed a 12 × 25 m maximal effort trial to determine critical swimming speed. One week later, participants performed two separate trials in the moderate and severe domains. Capillary blood (1 mL) was collected pre‐ and post‐exercise for DNA methylation profiling via reduced representation bisulfite sequencing. Global methylation and differentially methylated loci (DMLs) and regions (DMRs) were assessed using logistic regression. Exercise induced global hypomethylation regardless of intensity (severe domain: 0.25% difference; 95% CI: 0.023, 0.372; *P*_adj_ = 0.030; moderate domain: 0.17% difference; 95% CI: 0.012, 0.306; *P*_adj_ = 0.037). In contrast, DMLs and DMRs were largely intensity‐specific, with <10% of DMLs and no DMRs shared between domains. Promoter DMRs were identified in immune‐related genes: *ARHGDIA* (−13.5% in the severe domain) and *RNF7* (+20.6% in the moderate domain). Although global methylation patterns were consistent between swimming trials, exercise intensity‐specific DNA methylation signatures were detectable from capillary blood samples. These results highlight the potential of epigenetic profiling for personalised monitoring of training responses and athlete health.

## Abstract (highlights)

- **What is the central question of this study?**
  Does swimming exercise performed at low and high intensities elicit distinct DNA methylation responses in trained athletes and can this be detected from a 1 mL capillary blood sample?
- **What is the main finding and its importance?**
  Exercise altered global hypomethylation regardless of intensity, but most differentially methylated loci and regions were intensity specific. Promoter methylation changes occurred in immune‐related genes. These findings demonstrate that distinct, intensity‐dependent DNA methylation signatures can be detected from small blood samples, supporting the potential of epigenetic profiling for monitoring athlete training and health.

## 1 INTRODUCTION

The ability to monitor and respond to the internal physiological state of an athlete is critical for optimising performance, recovery and long‐term health in elite swimming. While traditional monitoring strategies rely on physiological, biochemical and subjective wellness metrics, advances in molecular profiling are opening new avenues for more precise, personalised health insights. Among these molecular profiling methods, epigenetics is emerging as a promising tool given its capacity to offer insights into the exercise responses of individual athletes (Etayo‐Urtasun et al., 2024; Goldsmith et al., 2021; Jacques et al., 2023).

Epigenetics refers to heritable changes in gene function that do not involve changes in the DNA sequence. Epigenetic modifications influence chromatin remodelling, by causing structural changes in how DNA is packaged within the nucleus. In turn, these changes alter the accessibility of genes to the transcriptional machinery (i.e., proteins involved in gene transcription) governing whether genes are transcriptionally active (‘turned on’) or silenced (‘turned off’). One of the most extensively studied epigenetic mechanisms is DNA methylation (5mC), which is the addition of methyl‐groups to cytosine (C) nucleotides. This modification most commonly occurs at cytosine nucleotides (C) that are immediately followed by guanine nucleotides (G), known as CpG sites. Across the human genome, DNA methylation is widespread, with approximately 70–80% of CpG sites being methylated in most cells. When DNA methylation occurs in regulatory regions of the genome, including gene promoters, it is highly correlated with gene silencing. Through these mechanisms, DNA methylation contributes to the regulation of genes and biological pathways.

DNA methylation is responsive to exercise, nutrition, stress and immune activation (Etayo‐Urtasun et al., 2024; Fesneau et al., 2024; García‐García et al., 2024; Goldsmith et al., 2024, 2021; Jacques et al., 2022), all of which contribute to training adaptations and post‐exercise recovery. Unlike transient fluctuations in gene expression or protein abundance, DNA methylation provides a more stable molecular record of health, as methylation marks can persist over longer time scales, across cell divisions, and reflect cumulative environmental and physiological exposures over time (Etayo‐Urtasun et al., 2024). Moreover, cells and tissues exhibit epigenetic memory, whereby prior training induces lasting alterations in the epigenetic landscape (i.e., the overall pattern of epigenetic modifications across the genome that determines which genes are active or inactive in a cell) that influence subsequent responses to exercise (Haupt et al., 2022; Seaborne et al., 2018). Seaborne et al. (2018) showed that in skeletal muscle, resistance training modified DNA methylation at genes involved in hypertrophy and metabolic regulation, leaving these genes epigenetically ‘primed’ even after periods of detraining enabling a more rapid adaptive response during retraining. The longitudinal stability of DNA methylation in response to exercise makes it a promising biomarker for monitoring training load, recovery status and readiness to train in athletes (Ding et al., 2026). As peripheral blood contains a heterogeneous mixture of immune cells that respond dynamically to physiological stressors such as exercise, blood‐based DNA methylation profiles can provide a systemic snapshot of immune activation, inflammatory signalling and metabolic stress that is relevant to athlete recovery and adaptation.

Characterising how exercise intensity influences DNA methylation signatures in accessible biological samples, such as capillary blood, may provide new opportunities for molecular profiling in sport. Despite the potential applications, epigenetic profiling in elite sport is in its infancy. While epigenetic responses to exercise have been studied in diseased populations, in particular elderly adults and cancer survivors, few studies to date have examined how exercise affects epigenetic signatures in the blood of trained individuals (Etayo‐Urtasun et al., 2024; Hunter et al., 2019). In addition, there is a critical gap in research using minimally invasive sampling approaches that could be feasibly implemented outside of the clinic or laboratory, such as small samples of capillary blood. Such innovations are essential for translating molecular tools from the laboratory to the daily training environment.

In this study, we profiled CpG methylation in 1.0 mL finger‐prick capillary blood samples collected from well‐trained (Tier 3) (McKay et al., 2022) male and female swimmers. Samples were collected before and after swimming trials performed within the moderate (below critical swimming speed) and severe (above critical swimming speed) exercise intensity domains, which were based on swimmers’ critical swimming speed calculated from a 12 × 25 m swimming test. We assessed the feasibility of detecting exercise‐induced DNA methylation changes in capillary blood using a minimally invasive protocol to characterise global and locus‐specific epigenetic responses to exercise in different exercise intensity domains. Our approach lays a foundation for DNA methylation profiling methods to be developed for individualised athlete monitoring that can be implemented at the poolside.

## 2 METHODS

### 2.1 Ethics

Ethical approval for this study was granted by the Human Research Ethics Committee, La Trobe University (HEC21001) and conformed to the Declaration of Helsinki. Written informed consent was obtained from all participants before data collection.

### 2.2 Study design

**FIGURE 1:**
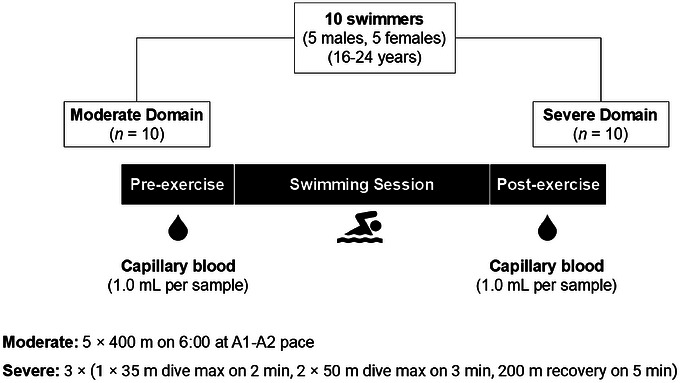
Study design schematic. Ten participants (five males, five females) performed a 12 × 25 m critical speed test approximately 1 week before the test week. During the testing week, participants performed two standardised swimming trials in the moderate, and severe exercise intensity domains, which were scheduled one day apart. To profile participants’ metabolic and epigenetic responses to exercise, a capillary blood sample (1.0 mL whole blood) was collected after 5 min of hand warming before and immediately after each standardised swimming trial.

This study employed a repeated measures design involving separate swimming trials in a 25‐m pool as described previously (Govus et al., 2025). As shown in Figure 1, 10 well‐trained swimmers performed a series of two separate swimming trials performed in the moderate (above critical swimming speed) and severe (above critical swimming speed) exercise intensity domains, scheduled one day apart in randomised and counterbalanced order. To profile participants’ epigenetic responses to exercise, a capillary blood sample (1.0 mL whole blood) was collected from their fingertip after 5 mins of hand warming before and immediately after each standardised swimming trials. Swimmers’ DNA methylation signature was analysed for the moderate and severe domain trials. Briefly, each standardised swimming trial was performed at the same time of day (∼06.30–07.00 h), swimmers were asked to abstain from caffeine and alcohol for 24 h before each exercise session and replicate their food intake (breakfast) before each testing session. After a standardised warm up (200 m easy swim, followed by 6 × 50 m efforts as 25 m pace/25 m recovery), swimmers performed the swimming trial in the moderate and severe intensity domains (Figure 1).

**TABLE 1:** Average lap times (s) and blood lactate concentrations (mmol/L) for male (*n* = 5) and female (*n* = 5) swimmers for trials performed in the moderate and severe intensity domains.

Variable	Moderate (5 × 400 m)		Severe [3 × (1 × 35 m, 2 × 50 m)]			
	Females (400 m)	Males (400 m)	Females (35 m)	Males (35 m)	Females (50 m)	Males (50 m)
Time (s)	308.6 ± 6.4	284.6 ± 12.8	18.6 ± 0.8	16.4 ± 0.2	28.1 ± 1.1	24.8 ± 0.3
						
Blood lactate (mmol/L)	1.3 ± 0.4	1.7 ± 0.7			8.6 ± 6.1	11.1 ± 3.5

Data are means ± standard deviation. Laps times have been averaged for each set where sets were performed.

### 2.3 Participants

Athletes were recruited from two swimming squads within Queensland, Australia. At the time of data collection, participants were ranked within the top 200 in their event within Australia. The top 10 participants (5 males, 5 females, age: 16–24 years) based on their World Aquatics points were included from a larger study of 16 athletes (Govus et al., 2025). Participants completed an online pre‐exercise medical screening questionnaire (ESSA Adult Pre‐Exercise Screening Form), were non‐smokers, free of metabolic disorders, and not taking any medication (other than oral iron supplements and/or asthma medication with an appropriate therapeutic use exemption). Female athletes taking hormonal contraceptives capable of altering the endogenous hormonal milieu for at least 3 months before testing were not excluded from the study.

### 2.4 Capillary blood collection, storage and transport protocols

Capillary whole blood (1.0 mL) was collected into a lithium heparin collection tube (Minicollect, Greiner Vacuette, Singapore) before and immediately after each swimming trial, following 5 min of hand warming to enhance local blood flow. The capillary blood samples were then centrifuged for 15 min at 1500 *g* to separate the plasma from the pelleted blood cells. Plasma and cells were stored on ice and then at −80°C within 2 h post‐collection. The separated samples were transported on dry ice via a medical courier for metabolic and epigenetic profiling.

### 2.5 DNA extraction

DNA was extracted from blood cells using the Genefind v3 whole blood DNA extraction kit (Beckman & Coulter, Brea, CA, USA) according to the manufacturer's instructions. DNA was eluted in nuclease free water and stored at −20°C until further analysis.

DNA quality control was assessed by measuring purity, concentration and fragment size distribution. DNA purity was evaluated using a NanoDrop One spectrophotometer (Thermo Fisher Scientific, Waltham, MA, USA) based on *A*_260_/*A*_280_ absorbance ratios, with acceptable values ranging from 1.8 to 2.0. DNA concentration was quantified using a Qubit 4 Fluorometer (Thermo Fisher Scientific) with the dsDNA High Sensitivity Assay to ensure accurate double‐stranded DNA quantification. Fragment size distribution and DNA integrity were assessed using the Agilent 2100 Bioanalyzer (Agilent Technologies, Santa Clara, CA, USA). Samples were considered to meet quality control criteria if they demonstrated appropriate purity (*A*_260_/*A*_280_: 1.8–2.0), sufficient concentration for library preparation (>500 ng/µL) and intact fragment profiles with minimal degradation. All samples met these criteria and were deemed suitable for downstream sequencing.

### 2.6 Reduced representation bisulfite sequencing

Genomic DNA (1000 ng) was used as the starting material. The genomic DNA spiked with lambda DNA was digested using methylation‐insensitive restriction enzyme *Msp*I to enrich in CpG dinucleotides, followed by end repair and dA‐tailing. The adapter ligation was performed by using adapters with all cytosines being methylated. After size selection, DNA fragments were bisulphite‐treated using Bisulfite kit DNA methylation Gold Kit (cat. no. D5005, Zymo Research, Irvine, CA, USA). After the bisulphite conversion, unmethylated cytosines were converted to U (to T after PCR amplification), while methylated cytosines remained unchanged. The DNA library was ready after PCR amplification. The library kit NEBNext^®^ Ultra™ II DNA Library Prep Kit (cat. no. E7645B, New England Biolabs, Ipswich, MA, USA) was used before DNA was sequenced using an Illumina NovaSeq 6000 PE150 S4‐Xp flow cell (Illumina, San Diego, CA, USA). Paired‐ended 150 bp reads were obtained with a minimum of 10 GB of raw data per sample.

### 2.7 Raw read processing and quantification of DNA methylation

Low quality reads, short reads and adapters were trimmed from fastq files using Trim Galore (version 0.6.10) with default parameters for paired‐end reduced representation bisulfite sequencing (RRBS) fastq files (PHRED > 20, read length > 20 bp), before mapping to the human genome (hg38) using Bowtie2 (version 2.5.2) (Langmead & Salzberg, 2012). CpG methylation was retrieved using the Bismark methylation extractor with flags for paired, bisulphite and RRBS data (version 0.24.2) (Krueger & Andrews, 2011). Briefly, CpG methylation frequency for individual cytosines (C) in the genome was calculated as the ratio of the number of alignments with C that were methylated over the total coverage (methylated + unmethylated) denoted as β‐values:

$$\beta =\frac{\mathrm{Methylated}\;\mathrm{Cs}}{\left(\mathrm{Methylated}+\mathrm{Unmethylated}\;\mathrm{Cs}+100\right)}$$

β ranges from 0 to 1 with values around 0 being unmethylated (0%) and values of 1 being fully methylated (100%).

### 2.8 Determining capillary blood DNA methylation stability between testing days

To quantify the stability of DNA methylation at individual CpG loci over time within the same participant, intraclass correlation coefficients (ICCs) were estimated. ICCs are widely used in longitudinal and reliability studies to partition the total observed variance into between‐subject and within‐subject components (Zaimi et al., 2018). ICCs were determined to measure the proportion of total variance that is attributable to between‐subject differences versus within‐subject variability between pre‐ and post‐exercise methylation levels (*M*‐values) for each subject and CpG site. *M*‐values are useful measures of methylation to prevent bias arising from heteroscedasticity (unequal variance) seen when analysing beta values. *M*‐values are described as:

$$M=\mathrm{lo}g_{2}\left(\frac{\beta }{1-\beta }\right)$$

The ‘irr’ package (version 0.84.1) was used, which implements a two‐way mixed effect, single rater, absolute agreement ICC. This approach modelled all subjects repeated measurements (pre‐exercise vs. post‐exercise) for each CpG site. Two‐way mixed, absolute agreement ICC accounts for both subject differences and repeated measures.

Mathematically, if $Y_{ij}$ represents the methylation measurement for the i ‐th subject at the j ‐th time point, the ICC is defined as:

$$\mathrm{ICC}\;=\frac{\sigma_{\mathrm{between}}^{2}}{\sigma_{\mathrm{between}}^{2}+\sigma_{\mathrm{within}}^{2}}$$

where $\sigma_{\mathrm{between}}^{2}$ is variance between individuals, and $\sigma_{\mathrm{within}}^{2}\;\mathrm{is}\;$ variance within individuals over time. ICC ranges from 0 to 1, where 0 indicates all variation is within‐person (no stability) and 1 indicates all variation is between‐person (perfect stability over time).

These values were further categorised according to level of stability defined as follows: low stability is where ICC = 0.00–0.29, indicating loci with high within‐subject variation relative to between‐subject variation; moderate stability is where ICC = 0.30–0.74, indicating loci with intermediate reproducibility; and high stability is where ICC ≥ 0.75, representing loci with strong within‐subject stability over time.

### 2.9 Global DNA methylation

CpG sites were retained for analysis if they met a minimum coverage threshold of ≥10 reads across samples ensuring that methylation estimates were based on sufficient sequencing depth to be reliable (Wreczycka et al., 2017). To focus on CpG sites exhibiting biological variability, CpGs with low variability across samples (standard deviation < 2%) were excluded. Following filtering, 2,421,531 CpG sites were retained for global methylation analyses. To provide an interpretable summary of methylation changes across the genome, global methylation summaries were derived by aggregating CpG‐level methylation proportions within biologically defined genomic features. Promoter methylation was defined as CpG sites located within 2000 bp upstream and 200 bp downstream of annotated transcription start sites (TSS), yielding 22,048 CpGs. Gene body methylation was defined as CpG sites located downstream of TSS and upstream of annotated stop codons, yielding 25,922 CpGs. To avoid pseudo‐replication arising from the large number of CpG sites and to respect the paired study design, global, promoter and gene body methylation were summarised at the participant level. For each participant and time point (pre‐ and post‐exercise), the median methylation proportion across CpGs within each genomic feature was calculated. The Shapiro–Wilk test of normality and Student's paired *t*‐test were performed comparing individuals’ pre‐ and post‐exercise median DNA methylation levels for both the severe and moderate intensity domains. Statistical significance was accepted as *P* < 0.05.

### 2.10 Estimation of immune cell frequencies from DNA methylation

Blood is a heterogenous tissue comprising multiple immune cell types, each of which has a distinct methylation pattern (Goldsmith et al., 2024). As a result, changes observed in whole blood DNA methylation profiles can represent not only true biological variation within cells but also shifts in the relative abundance of different immune populations. Thus, estimating immune cell frequencies in blood can allow for better interpretation of methylation changes by helping to distinguish between cell composition effects and cell‐intrinsic methylation changes. We applied a reference‐based deconvolution approach using the EpiDISH R package (version 2.18.0) (Zheng et al., 2020). This method infers cell‐type proportions by leveraging publicly available DNA methylation reference profiles of immune cell subsets isolated by fluorescence activated cell sorting. We used reference data sets (Gervin et al., 2016; Lucassalas, 2018) which define cell‐type specific methylation signatures at informative CpG sites. EpiDISH implements a constrained projection algorithm that models the bulk methylation profile of each sample as a linear combination of these reference signatures. The estimated coefficients from this model correspond to the relative proportions of major immune cells including B cells, CD4^+^ T cells, CD8^+^ T cells, granulocytes, neutrophils and eosinophils. Methylation β‐values of CpG sites from each participant were used as input to the deconvolution model to estimate immune cell frequencies. Differences between participant cell frequencies were detected using a Kruskal–Wallis rank sum test. Significant relationships were indicated at *P* < 0.05.

### 2.11 Detection of differential DNA methylation

As with global DNA methylation analysis, after filtering out CpG sites with low sequencing coverage (<10×) and minimal variability across samples (standard deviation <2%), differential methylation analysis was conducted on 2,421,531 CpG sites using the *methylKit* package in R (version 1.26.0) (Akalin et al., 2012). Differentially methylated loci (DMLs) were determined using logistic regression. To address extra variability beyond that expected by the model, an overdispersion correction was applied using the method of McCullagh & Nelder (1989). To account for repeated measures and inter‐individual biological differences, participant ID was included in the model as a fixed effect covariate. To correct for multiple hypothesis testing, a standard false discovery rate (FDR)‐based method (Benjamini–Hochberg) was applied to correct *P*‐values using the *methylKit* package. Significant DMLs were accepted at a Benjamini–Hochberg adjusted *P*‐value (*P*_adj_) < 0.05. Differentially methylated regions (DMRs) were then determined by clustering a minimum of two adjacent significant DMLs of the same directionality within 500 bp. The statistical significance of DMRs was computed by combining individual CpG Benjamini–Hochberg adjusted *P*‐values in each region using Stouffer's *Z*‐score with the R package *metap* (version 1.12) (Dewey, 2014). Significant relationships were identified as *P* < 0.001.

### 2.12 Data availability and analysis code

The code to reproduce analyses conducted in this manuscript is available on the project's GitHub repository: ChloeDG/SPP01.

## 3 RESULTS

### 3.1 Standardised swimming trials

The results from the cohort characteristics and standardised swimming trials for the included athletes are summarised in Table 1.

### 3.2 General DNA methylation patterns and stability

**FIGURE A1:**
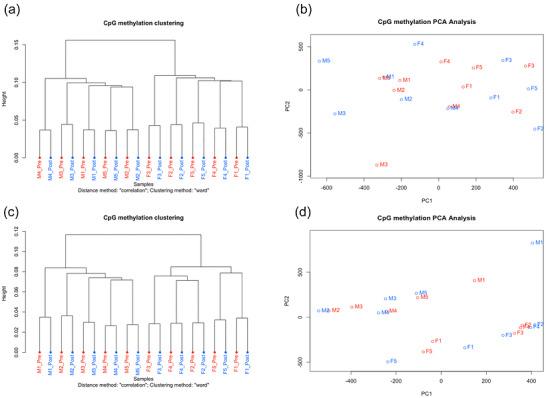
DNA methylation data clustering for the severe (a) and moderate domain trials (c). PCA clustering samples based on all CPG sites after filtering for males (M) and females (F) in the severe (b) and moderate intensity domain trials (d).

To determine levels of DNA methylation in capillary blood of the swimmers pre‐ and post‐exercise in different intensity domains, we used reduced representation bisulfite sequencing (RRBS). RRBS promotes genomic regions that are rich in CpG sites (locations in DNA where a cytosine nucleotide occurs next to a guanine nucleotide and methylation commonly occurs) using the restriction enzyme *Msp*I, which cuts DNA at all CCGG sites, enabling measurement of DNA methylation at ∼10% of all CpG sites in the mammalian genome. This approach focuses on regions where methylation is classically understood to link to biological function. To first assess overall data structure and confirm data quality prior to downstream analysis, we examined global methylation patterns across all samples. To identify general methylation patterns, a principal component analysis (a statistical method that reduces complex datasets to reveal major sources of variation between samples) was performed on DNA methylation levels in all samples after filtering and normalisation (Appendix Figure A1). CpG methylation clustering separated the samples by sex in the first node of the dendrogram, followed by participant and then exercise intensity domain (moderate or severe), highlighting the similarity of samples clustered together.

**FIGURE A2:**
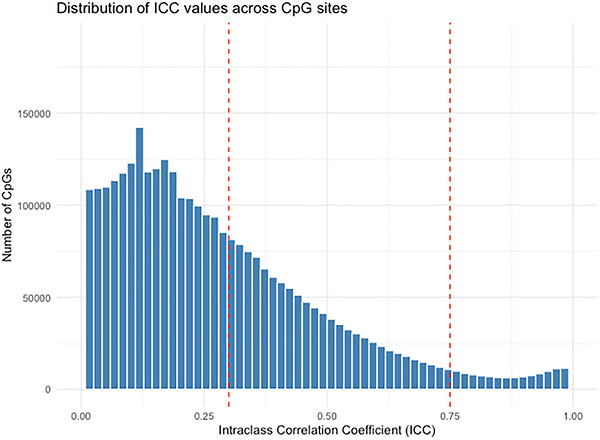
Intraclass correlation coefficient (ICC) determined for each CPG site. These values were further categorised according to level of stability: low stability: ICC = 0.00–0.29, indicating loci with high within‐subject variation relative to between‐subject variation; moderate stability: ICC = 0.29–0.74, indicating loci with intermediate reproducibility; and high stability: ICC ≥ 0.75, representing loci with strong within‐subject stability over time.

To identify the stability of DNA methylation between testing days, ICC values were determined for individual CpG sites (Appendix Figure A2). The ICC measures the reproducibility of methylation measurements across repeated samples and helps to distinguish true biological signal from technical noise. Higher ICC values indicate CpG sites with more consistent methylation levels across time points whereas lower values indicate sites that vary more between measurements. ICCs were stratified into stability thresholds (low, moderate and high) and are presented as the percentage of CpGs falling into each category (Appendix Table A1). Sixty‐four percent of all CpGs included in the analysis fell into the low stability category, 32.3% into the moderate and 3.7% into the high stability category. This pattern of distribution indicates that many CpG sites display short term variability with those falling in the moderate and high categories representing more stable markers.

### 3.3 Estimation of immune cell frequencies from DNA methylation

**FIGURE A3:**
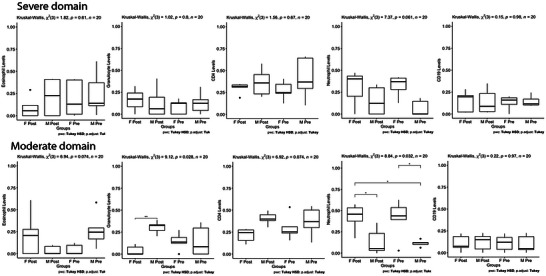
Estimation of immune cell frequencies from DNA methylation data from male (M) (*n* = 5) and female (F) (*n* = 5) swimmers. The *x*‐axis represents the ratio of each subset detected in the sample. Pre, pre‐exercise; Post, post‐exercise.

Since processing of bulk tissues can be affected by sample cellular composition, we used the DNA methylation levels in each sample and available reference datasets to estimate the frequencies of major immune cells in participant blood (Appendix Figure A3). We did not detect any significant differences between pre‐ and post‐exercise samples in either exercise intensity domain in any of the immune cells tested (*P*_adj_ = 0.06–0.80). It appears that the cellular composition of capillary blood is relatively stable, and thus any changes in methylation observed after exercise are more likely to reflect true biological regulation rather than changes in circulating immune cell frequencies.

### 3.4 Global methylation patterns after exercise

**FIGURE 2:**
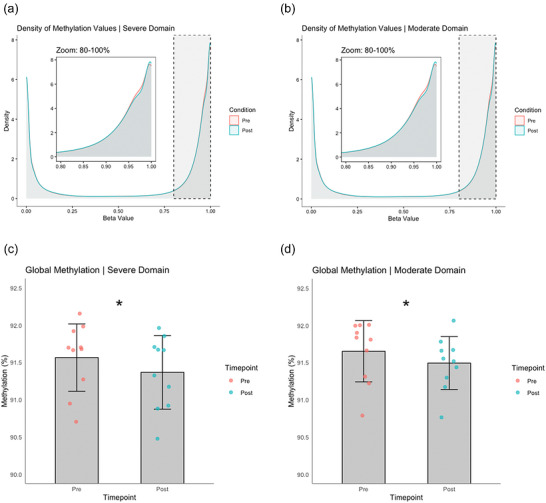
Global DNA methylation distribution in capillary blood cells of highly trained swimmers (*n* = 10). (a, b) Density plot describes the methylation profiles observed in response to exercise in the severe domain (a) and moderate domain (b). Density plots show the distribution of methylation at different methylation levels. Methylation levels on the *x*‐axis correspond to β‐values (0 to 1) where 0 corresponds to 0% methylation and 1 corresponds to 100% methylation. The density values on the *y*‐axis correspond to the relative density (probability density function) of β‐values, indicating the frequency of CpGs at each specific methylation level. The zoom boxes highlight the density between β‐values from 0.8 to 1.0 (i.e., 80–100% methylation). There were 2,421,531 CpG sites included in the analysis for each participant after filtering for pre‐ and post‐exercise conditions. (c, d) Median global methylation values for the severe domain trial (c) and moderate domain trial (d). Methylation values on the *y*‐axis correspond to percentage of methylation (%). Medians were calculated from all the included CpG sites. A paired *t*‐test was used to determine the difference between median methylation levels (severe domain: *P* = 0.030, moderate domain: *P* = 0.043). *Significance accepted at *P* < 0.05. Pre, pre‐exercise; Post, post‐exercise.

**FIGURE A4:**
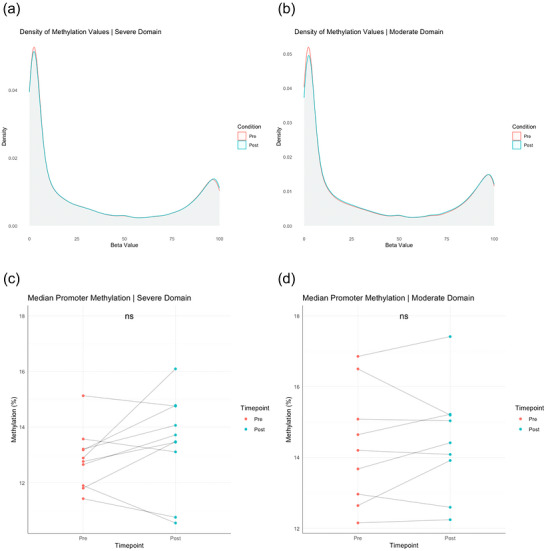
Gene feature DNA methylation patterns after different exercise sessions. Promoter methylation indicates the methylation of 22,048 CpGs, which includes those within 2000 bp upstream and 200 bp downstream of a transcription start site. Gene body methylation indicates 25,922 CpGs, which includes those from the transcription start site and final stop codon for sequenced genes. The red line indicates the medians. Paired *t*‐test of significance used to determine differences between pre‐ and post‐exercise. Significance value: *P* < 0.05. Pre, pre‐exercise; Post, post‐exercise.

Global DNA methylation patterns were determined for exercise performed in both the moderate and severe exercise intensity domains (Figure 2). Global methylation changes can indicate widespread shifts in gene regulatory activity in response to physiological stress such as exercise. To assess global shifts in DNA methylation, the distributions of pre‐ and post‐exercise methylation profiles were plotted as overlaying histograms (Figure 2a, b) allowing visualisation of whether methylation levels across the genome tended to increase or decrease after exercise. Median methylation levels were compared to determine the direction of global methylation changes (Figure 2c, d). Hypomethylation (a decrease in DNA methylation) was observed after exercise performed in both the severe and moderate domains (severe domain: −0.253% difference; 95% CI: 0.023, 0.372; *P*_adj_ = 0.030; moderate domain: −0.172% difference; 95% CI: 0.012, 0.306; *P*_adj_ = 0.037). Reductions in DNA methylation are often associated with increased accessibility of DNA and potential activation of gene expression, suggesting exercise induces genome‐wide regulatory changes in circulating blood cells. To better understand if this global hypomethylation was specific for a certain genomic region, we investigated the methylation distribution changes in promoters and gene bodies in response to exercise, as well as median methylation levels in the different genomic regions. Promoter regions regulate the initiation of gene transcription, while gene bodies often reflect transcriptional activity and regulatory fine tuning. Here, median methylation directionality patterns were inconsistent between participants, and no significant relationships were observed (Appendix Figure A4). Taken together, it appears that the global methylation shift observed after exercise was not driven by uniform changes in specific regulatory regions.

### 3.5 Differential DNA methylation after exercise was unique to intensity domain

**FIGURE 3:**
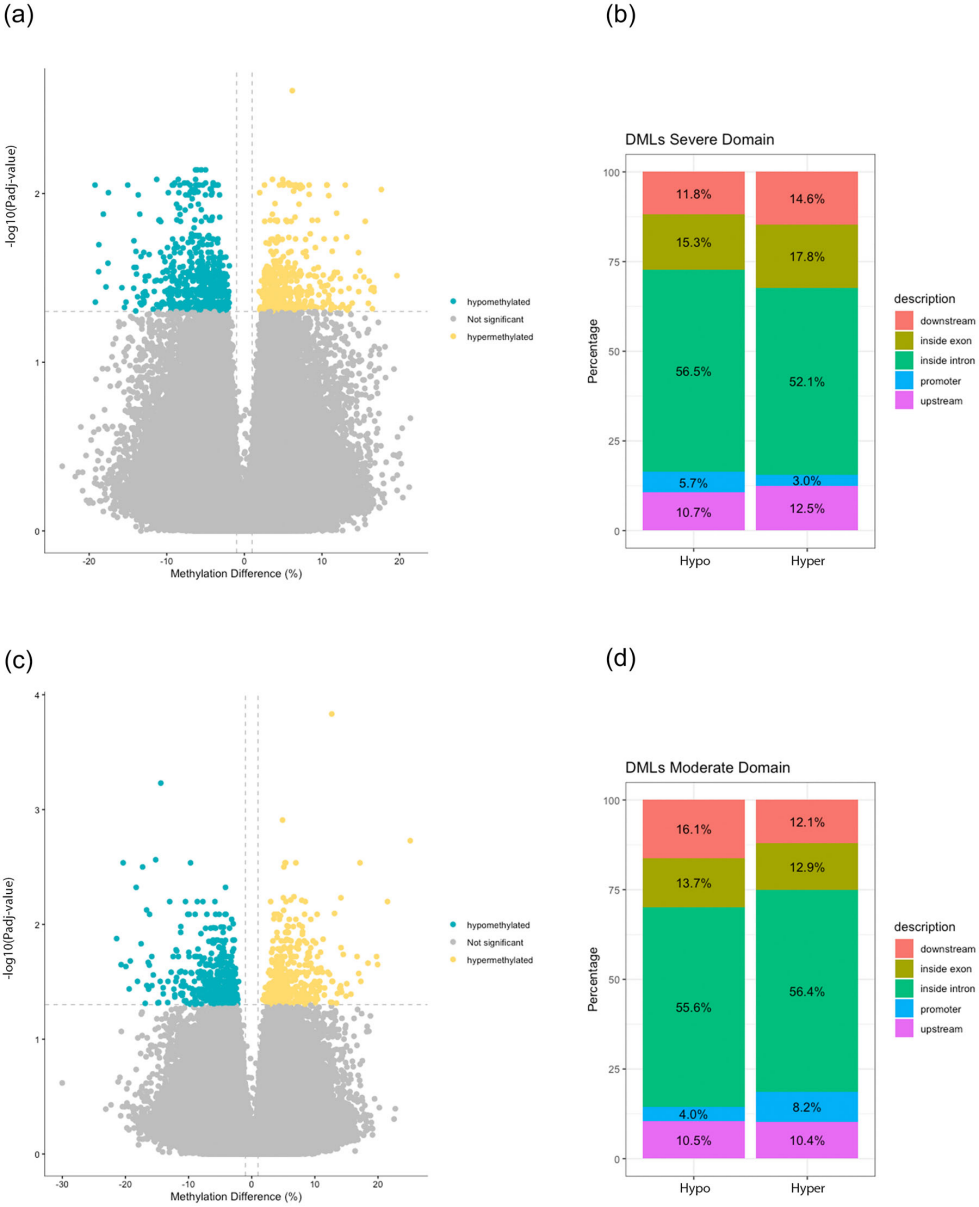
Differentially methylated loci (DMLs) in capillary blood from 10 (five males, five females) highly trained swimmers after exercise in the moderate and severe intensity domains. (a, c) Volcano plot representing all 2,421,531 CpG sites included in severe domain (a) and moderate domain (c) analysis after filtering. Methylation difference on the *x*‐axis indicates the percentage change (%) in methylation levels pre‐ compared post‐exercise. The *y*‐axis indicates the log_10_(*P*_adj_‐value). Methylation difference values >1 or <1 with significance level of −log_10_(*P*_adj_‐value) = *P*_adj_‐value < 0.05 are highlighted as a hyper‐ or hypo‐ methylation change, respectively. (b, d) Genomic distribution of DMLs in positive and negatively methylation detected in severe (b) and moderate (d) domain analysis. Percentage of significant DMLs falling in each genomic region. Total DMLs detected after exercise performed in the severe domain was 895 and moderate domain was 1032.

To determine the effects of exercise on the DNA methylation of individual and regions of CpGs, we identified DMLs comparing pre‐ and post‐exercise samples. Thus, DMLs represent genomic positions where methylation levels change significantly between conditions. A total of 895 significant DMLs were identified (*P*_adj_ < 0.05) when comparing pre‐exercise and post‐exercise samples in the severe intensity domain. Similarly, 1032 DMLs were identified in the moderate intensity domain trial (Figure 3a, c). This is evidence that exercise in both the severe and moderate intensity domains induced widespread locus‐specific changes in capillary blood cells. Importantly, the genomic location of methylation changes around and within a gene region is an important factor for influencing gene expression, as methylation observed in different genomic regions (i.e., promoters, gene bodies, etc.) will contribute differently to gene regulation. As such, we next sought to determine the distribution of DMLs throughout different genomic features including promoters, introns, down and upstream of genes (Figure 3b, d); the largest number of DMLs mapped to introns, followed by downstream and upstream of gene locations with promoters having the least. No significant differences were observed in the distribution of DMLs between hypo‐ and hyper‐methylated groups or between different domain intensity sessions, indicating that exercise did not preferentially affect one genomic feature or direction of methylation change across intensity conditions.

**FIGURE 4:**
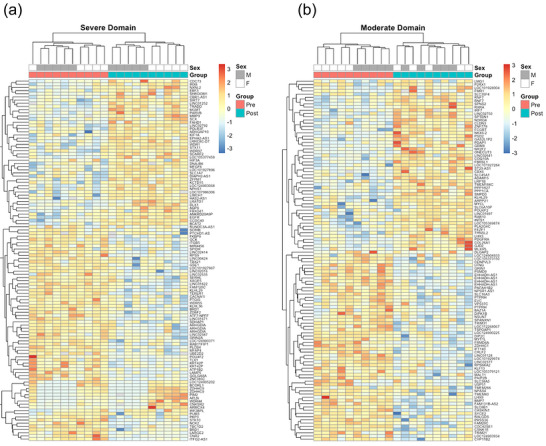
Differentially methylated loci (DMLs) greater than 10% in capillary blood from 10 swimmers (five males, five females) after exercise in the moderate and severe intensity domains. (a) Heatmap representing the 114 significant DMLs exhibiting greater than 10% change after the severe intensity domain session. (b) Heatmap representing the 112 significant DMLs exhibiting greater than 10% change after the moderate intensity domain session. Rows represent individual DMLs annotated with their closest gene, clustered on the left axis based on the similarity between methylation levels between CpG sites so that loci with similar patterns are grouped together. Columns represent individual participants, with dendrogram positions based on the overall methylation patterns across included DMLs, grouping individuals with similar responses. Darker red indicates higher DNA methylation, darker blue represents lower DNA methylation. Significance level: > ± 10% methylation change, *P*_adj_‐value < 0.05. Pre, pre‐exercise; Post, post‐exercise.

To identify DMLs with greater biological importance, we applied a larger threshold (> ± 10%) to prioritise methylation changes that are more likely to have functional effects. This approach identified 112 DMLs in the moderate and 114 DMLs in the severe domain trials (Figure 4a, b). To identify genes potentially affected by exercise, the closest genes were mapped to each of these DMLs, which are represented on the *y*‐axis of Figure 4a, b. Hierarchical clustering of DMLs separated samples into pre‐ and post‐exercise conditions at the first node (Figure 4a, b) indicating that the methylation changes induced by exercise were sufficiently consistent to distinguish samples collected before and after exercise based solely on their DNA methylation profiles.

**FIGURE A5:**
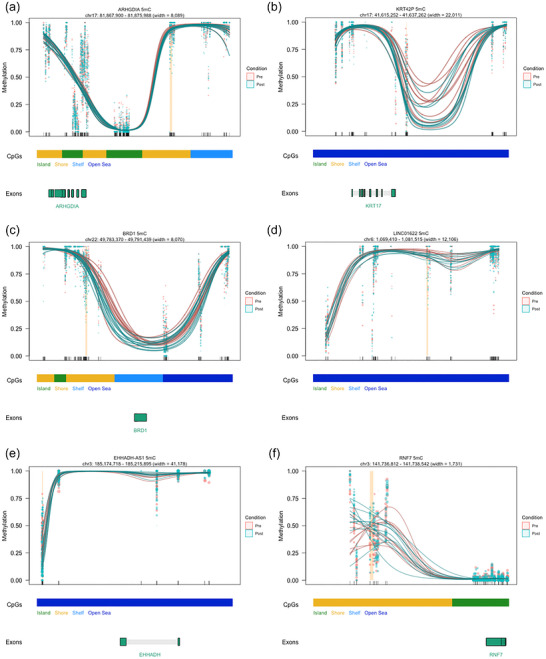
DNA methylation patterns of differentially methylated genes in capillary blood from 10 swimmers (five males, five females) after swimming trials in the moderate and severe intensity domains. (a–f) DMR plots are shown for significant DMRS with greater than 10% change in methylation in the severe intensity domain trial (a–d) and the moderate intensity domain trial (e–f). Each DMR plot depicts one DMR (highlighted orange). Methylation levels on the *y*‐axis correspond to β‐values (0–1) where 0 corresponds to 0% methylation and 1 corresponds to 100% methylation. The track below each plot provides information on the density of CPG sites and the overlap with annotated genes and exons. Exact genomic positions and annotations in the Supporting information. Pre, pre‐exercise; Post, post‐exercise.

To obtain a more robust measure of the DNA methylation variation after exercise, we searched for DMRs, which represent clusters of neighbouring CpG sites that change together. DMRs are therefore considered stronger indicators of biological meaning than changes at single CpG sites. We defined DMRs as genomic ranges of at least two CpG sites that were significantly different between pre‐ and post‐exercise, within 500 bp of one another. This analysis identified 10 DMRs in the severe and three DMRs in the moderate intensity domain trials (*q* < 0.05, Stouffer's *P* < 0.001) (Table 2 and Appendix Figure A5a–f), indicating that only a small subset of genomic regions exhibited coordinated methylation changes across multiple CpG sites following exercise irrespective of intensity. Identified DMRs mapped primarily to gene bodies and promoter regions (Appendix Figure A5a–f) which are commonly involved in regulating gene expression. Identified DMRs frequently overlapped with CpG island (CGI) regions (Appendix Figure A5a, c, f) CGIs are CpG rich regions often located near gene promoters and are key regulatory elements where DNA methylation can strongly influence gene activity. CpG shores and shelves are regions flanking CpG islands where methylation changes are often dynamic and responsive to environmental stimuli.

**TABLE 2:** Differentially methylated regions (DMRs) in capillary blood from 10 (five males, five females) highly trained swimmers detected after swimming trials performed in the moderate and severe intensity domains.

Intensity Domain	Name	DMR location	Chr	Start	End	Mean methylation difference	Number of CpGs	q‐value	Stouffer's P‐value
**Severe**	ARHGDIA	Promoter	chr17	81873400	81873488	−13.49	3	0.010	2.45×10^−17^
	BRD1	Inside intron	chr22	49785370	49785439	−14.09	2	0.022	3.83×10^−11^
	CEP192	Downstream	chr18	13137154	13137226	−4.52	2	0.038	8.27×10^−10^
	DDX10	Inside intron	chr11	108696269	108696368	4.93	2	0.029	2.45×10^−10^
	ELP4	Inside intron	chr11	31532917	31532929	−4.68	2	0.028	3.20×10^−10^
	KRT42P	Downstream	chr17	41625752	41625762	−18.71	2	0.009	6.04×10^−13^
	LINC01622	Inside intron	chr6	1076410	1076515	−12.88	2	0.010	1.48×10^−12^
	MAP7	Inside intron	chr6	136376319	136376378	−7.24	2	0.010	3.25×10^−11^
	PAX5	Inside intron	chr9	37027171	37027320	4.00	2	0.031	6.77×10^−10^
	ZDHHC9	Inside intron	chrX	129843490	129843527	16.03	2	0.033	7.45×10^−10^
**Moderate**	EHHADH‐AS1	Inside intron	chr3	185175718	185175895	−19.77	4	0.003	3.72×10^−22^
	PTPRH	Inside intron	chr19	55193689	55193694	−11.71	2	0.006	1.21×10^−11^
	RNF7	Promoter	chr3	141737312	141737342	20.64	2	0.006	9.73×10^−13^

DMRs were identified by clustering a minimum of two adjacent significant DMLs of the same directionality within 500 bp, indicating regions where nearby CpG sites showed coordinated increases or decreases in methylation. Start and end positions indicate the genomic coordinates of each DMR on their respective chromosome (Chr). Individual differentially methylation loci (DML) *P*‐values for each region were combined using Stouffer's *Z*‐score. Significance was accepted at *q* < 0.05.

DMRs were located within several genes associated with immune regulation, transcriptional control and cellular metabolism. In the severe domain trial, *DDX10* (DEAD‐box helicase 10), *PAX5* (paired box 5) and *ZDHHC9* (zinc finger DHHC‐type palmitoyltransferase 9) exhibited hypermethylation post‐exercise within intronic regions. Conversely, genes such as *ELP4* (elongator acetyltransferase complex subunit 4), *ARHGDIA* (Rho GDP dissociation inhibitor alpha) and *BRD1* (bromodomain containing 1) displayed hypomethylation on their promoters and introns (Table 2 and Appendix Figure A5c). *ARHGDIA* exhibited significant hypomethylation on a CGI shore on the promoter (Table 2 and Appendix Figure A5a). DMRs were also detected in the pseudogene *KRT42P* and the long non‐coding RNA *LINC01622* (Table 2 and Appendix Figure A5b, d). In the moderate intensity domain trial, *RNF7* (ring finger protein 7) showed hypermethylation post‐exercise in a CGI shelf on the promoter (Table 2 and Appendix Figure A5f). Conversely, hypomethylated DMRs were found within introns of *PTPRH* (protein tyrosine phosphatase receptor type H) and *EHHADH‐AS1* (EHHDH antisense RNA 1), with *EHHADH‐AS1* showing the largest decrease (Table 2 and Appendix Figure A5e).

A total of 77 DMLs (∼10%) were shared between the moderate and severe domain trials, leaving 818 DMLs unique to the severe and 955 unique to the moderate domain trials, with no DMRs shared between trials. These data further highlight that exercise conducted at different intensity domains has unique effects on CpG DNA methylation.

To better understand if the DMLs identified in this study represented a true underlying biological signal rather than technical or random variability, we examined the proportion of DMLs across different ICC stability categories (Appendix Table A1). Approximately 10% of DMLs from either swimming trial had a highly stable ICC and ∼25% had a moderate ICC. Compared with the distribution of ICC values across all CpG sites analysed, DMLs were enriched for highly stable CpG sites (+7%) and depleted in the moderate stability category (−10%). Thus, CpG loci identified as differentially methylated were more likely to occur at sites with reproducible methylation measurements across testing days, supporting the likelihood that these changes reflect true biological responses. The ICC of the remaining DMLs indicated low stability (66% and 55% for moderate and severe swimming trials, respectively), indicating these CpG sites may be more susceptible to variability and should be interpreted with more caution.

## 4 DISCUSSION

This study explored DNA methylation responses to swimming exercise performed in the moderate and severe exercise intensity domains in well‐trained male and female swimmers. Understanding how exercise alters epigenetic regulation provides insight into the effects on gene activity and immune function in athletes. Capillary blood samples (1.0 mL) were collected by finger prick obtained in an ecologically valid training environment. Capillary blood sampling provides a minimally invasive method that can be used directly during athlete training sessions making it attractive for real‐world monitoring of molecular responses to exercise. We first performed an analysis to determine the stability of individual baseline CpG site DNA methylation in capillary blood samples collected on different days using ICC. The number of highly stable CpGs was in accordance with similar DNA methylation studies (Flanagan et al., 2015; Jiang et al., 2020; Zaimi et al., 2018) indicating good reproducibility. This outcome supports the feasibility of using capillary blood for detecting DNA methylation, expanding opportunities for field‐based athlete monitoring research.

### 4.1 Global hypomethylation in response to swimming exercise

We observed global hypomethylation after exercise, irrespective of exercise intensity indicative of a broad relaxation of chromatin structure. This response is consistent with an earlier study examining the effect of acute aerobic exercise (45 min cycling at 70% *W*_max_) on global DNA methylation in trained cyclists (Hunter et al., 2019). Here the authors reported that acute exercise induced global DNA hypomethylation in peripheral blood leukocytes. It appears that methylation responses measured in capillary blood are comparable to those previously reported in venous blood leukocytes, supporting the use of capillary blood sampling for molecular studies. Our findings add to this by showing that global hypomethylation occurring after exercise is not intensity specific. Taking this further, DNA methylation responses to exercise in global promoter and gene body regions were inconsistent in terms of the directionality with both hypo‐ and hyper‐methylation observed in different participants irrespective of exercise intensity. Thus, global hypomethylation after acute exercise represents a significant but subtle shift in the epigenetic landscape that may relate to cumulative small changes across the genome rather than large scale changes in specific functional domains. Such distribution changes across many genomic loci may collectively influence regulatory networks. Nevertheless, these data indicate a role for exercise in chromatin remodelling of capillary blood cells.

### 4.2 Exercise conducted in different intensity domains elicits a specific effect on DNA methylation

We have demonstrated that a single bout of exercise may elicit intensity‐dependent changes in DNA methylation in capillary blood, with distinct DMLs and DMRs observed following exercise in the moderate and severe intensity domains. These data indicate that different exercise intensities activate distinct physiological signalling pathways that are reflected in the DNA methylation profiles of circulating blood cells. While formal ontology or pathway analysis was not performed due to the modest number of DMRs identified, the genes nearest to these regions are associated with plausible biological functions. Specifically, exercise in the severe domain induced methylation changes in genes related to transcriptional regulation (*DDX10*, *ELP4* and *BRD1*), immune function (*PAX5*, *ARHGDIA*) (Wei et al., 2018) and cellular motility (*ZDHHC9*). Exercise in the moderate domain influenced genes involved in immune function (*RNF7*) (Blondelle et al., 2020) and metabolism (*EHHADH‐AS1*) (Houten et al., 2012). Given that capillary blood is rich in immune cells, the enrichment of immune‐related genes is not unexpected. Moreover, exercise‐associated methylation changes observed in other tissues have also been linked to similar biological processes such as immune function and transcriptional regulation (Houten et al., 2012). Consistent with this paradigm, we have reported elsewhere the effects of acute exercise on metabolism in this cohort (Govus et al., 2025). Taken together, these findings suggest that the observed methylation changes reflect a coordinated biological response to acute exercise, involving both metabolic and immune regulatory processes.

To our knowledge, this is the first study to report acute exercise‐induced changes in the DNA methylation patterns of capillary blood in highly trained individuals, highlighting the potential for capillary blood epigenetic profiling to be used in athlete monitoring and exercise physiology research. While this context is novel, our findings are consistent with other human intervention studies demonstrating that acute exercise can induce detectable DNA methylation changes in circulating blood cells. Importantly, however, the reported effects appear to vary depending on exercise type, intensity and analytical methods used to measure DNA methylation. For example, one pilot study investigated the effects of acute aerobic exercise using an incremental step test performed on a bicycle ergometer in five healthy women (Schenk et al., 2019). This study collected peripheral NK cells and used Illumina EPIC methylation arrays to detect DNA methylation. A total of 33 DMLs and 19 DMRs were identified comparing pre‐ and post‐exercise samples; however, none of these methylation changes overlap with the present study. Similarly, another study investigated the effect of a 5 km treadmill time trial on DNA methylation by Illumina Infinium methylation array in peripheral blood from eight healthy young males (Robson‐Ansley et al., 2014). No direct changes were identified in methylation after the intervention but 11 DMLs were correlated with changes in cytokine level, specifically interleukin‐6, after exercise.

The differences between these studies and the present study likely reflect a combination of biological and methodological factors. Firstly, both studies were focused on participants from a single biological sex, while the present study enrolled both male and female participants. DNA methylation is known to vary between male and female participants, particularly in blood, and sex‐specific responses to physiological stressors such as exercise may therefore contribute to divergent results. However, longitudinal exercise‐induced DNA methylation changes in other tissues (muscle) have been shown to be minimally affected by biological sex after training interventions (Landen et al., 2023). Nevertheless, sex‐related differences in blood‐based methylation responses following acute exercise cannot be excluded. Secondly, the analytical resolution (i.e., number of CpGs tested) of DNA methylation profiling differed substantially between studies. Both prior studies employed methylation array‐based approaches, which assess approximately 25,000 CpG sites, while RRBS, used in the present study, interrogates methylation in roughly 3.5 million CpG sites across the genome. This substantially greater genomic coverage increases the potential for detecting exercise‐responsive methylation changes and provides a more comprehensive view of genome wide methylation dynamics. Moreover, training status may also contribute to the observed discrepancies. Participants in the present study were highly trained, while previous studies examined untrained individuals. Exercise induced DNA‐methylation responses have been shown to differ in other tissues, such as skeletal muscle (Geiger et al., 2024). Lastly, the effect of different exercise protocols and their potential to affect the DNA methylation landscape cannot be excluded. Previous studies employed different exercise protocols to the current study, which could play a role in disparate biological responses we observed. Taken together the differential methylation observed in the present study and differences between previous studies likely reflect meaningful biological variation combined with differences in study design, tissue type and analytical approach.

Characterising the dynamic nature of exercise‐induced epigenetic regulation provides useful insights into how exercise can modulate athlete phenomics through changes in DNA methylation. These findings extend the growing body of evidence that exercise can acutely remodel the human epigenome and provide novel insight into the blood‐based methylation signatures associated with exercise intensity.

### 4.3 Study limitations

We acknowledge several limitations in this study, which stem from efforts to maintain ecological validity when working with high‐performance athletes. Firstly, our sample size was limited to 10 athletes, a constraint that is not uncommon in high‐performance sport research. To address this limitation, we applied stringent statistical analyses to detect global patterns and differential methylation. Secondly, we used capillary blood sampling to minimise disruption to athletes and enable repeated measurements in a performance‐focused setting. However, capillary blood sampling yields small volumes (∼1 mL) which may increase sample biological variation. To address this limitation, we performed additional analysis to determine the daily reproducibility and stability of methylation levels in this cohort with this sampling method, finding that the percentage of stable CpGs was comparable to venous blood specimens. Nevertheless, a direct comparison between capillary and venous blood CpG DNA methylation is warranted and should be considered for future studies.

Thirdly, our study was conducted outside of a fully controlled laboratory environment. DNA methylation can be affected by environmental and lifestyle factors and while efforts were made to standardise conditions as much as possible, some uncontrolled variability is unavoidable in applied field‐based research. Fourthly, the RRBS method has its own limitations. While it offers more genomic coverage than other approaches like methylation arrays, it still only captures ∼10% of the human genome, resulting in lower resolution than whole genome bisulfite sequencing, long read sequencing methods like single‐molecule sequencing by Oxford Nanopore Technologies, or smart‐seq by PacBio platforms. Lastly, our study focused on high performance swimmers, performing swimming exercise, and thus considering the specificity of the cohort and DNA methylation changes observed in the different intensities, caution is warranted when extrapolating these findings to other sports, exercise modalities or untrained populations.

### 4.4 Conclusion and future directions

While global DNA methylation patterns exhibit similar trends after acute exercise performed in different exercise intensity domains, distinct epigenetic signatures emerge in highly trained swimmers. Specifically, hypermethylation in promoters and gene bodies occurred irrespective of intensity, whereas DMLs and DMRs were highly session‐specific, with ∼10% overlapping between exercise performed in the severe and moderate intensity domains. By leveraging minimally invasive capillary blood sampling, this research advances molecular profiling in sport science, providing a foundation for personalised monitoring of epigenetic responses to exercise. These methods pave the way for using molecular profiling to optimise training strategies to enhance athlete health and performance. Integrating information from additional ’omic layers (e.g., genome, transcriptome, proteome and gut microbiome) and from skeletal muscle and biofluids such as urine and saliva, will provide a more holistic understanding of the molecular responses to in high‐performance athletes in the future.

### 4.5 Perspective

The use of capillary blood from a 1.0 mL finger‐prick sample enables repeated, minimally invasive sampling of DNA methylation in athletes in ecologically valid training settings. Our findings provide proof‐of‐concept that epigenetic signals in small blood volumes can yield insights into systemic immune and metabolic responses to exercise. As technologies mature, these data support the development of biomarker platforms for real‐time monitoring of athlete immune health, training adaptation and recovery status. Such platforms could integrate methylation data with metabolomics and other molecular layers to deliver personalised training feedback, helping to reduce risk of illness or overtraining in highly trained athletes.

## AUTHOR CONTRIBUTIONS

A. D. Govus, L. J. G. Mitchell, N. G. Lawler, C. D. Goldsmith, D. B. Pyne and K. McGibbon designed the study. A. D. Govus, L. J. G. Mitchell, N. G. Lawler, C. D. Goldsmith and K. McGibbon collected the data. C. D. Goldsmith and M. Kozlovskaia conducted DNA extraction. C. D. Goldsmith performed epigenetic profiling and related bioinformatics. A. D. Govus and N. G. Lawler performed the bioinformatics for metabolomics data. A. D. Govus, L. J. G. Mitchell, N. G. Lawler, C. D. Goldsmith, D. B. Pyne, M. Kozlovskaia and K. McGibbon wrote the manuscript. All authors have read and approved the final version of this manuscript and agree to be accountable for all aspects of the work in ensuring that questions related to the accuracy or integrity of any part of the work are appropriately investigated and resolved. All persons designated as authors qualify for authorship, and all those who qualify for authorship are listed.

## CONFLICT OF INTEREST

The authors declare no conflicts of interest.

## Supporting information



Table S1. Table of all significant DMLs identified in the severe intensity domain trial.

## 1

**TABLE A1:** Percentage of DMLS falling into different intraclass correlation coefficient (ICC) categories.

ICC	All CpGs (counts)	Percentage of all CpGs with ICC values in each category	Percentage of DMLs with ICC values in each category: moderate	Percentage of DMLs with ICC values in each category: severe
Low (0.00–0.30)	2,082,445	64.0	66.4	54.8
Moderate (0.30–0.75)	1,050,012	32.3	23.6	26.1
High (>0.75)	122,034	3.7	10.0	10.4

Intraclass correlation (ICC) values were calculated on β‐values for each CPG site included in the analysis with greater than 10× coverage. DMLS included were greater than 10% difference in methylation between pre‐ and post‐exercise with a *q*‐value less than 0.05. Moderate domain session = 110 DMLS and severe domain session = 115 DMLS.
