# Supplementary material for: Acute capillary blood DNA methylation responses to swimming exercise in high‐performance male and female swimmers
Source: Exp Physiol. 2026 Jun 12;111(8):3648–66. doi: 10.1113/EP093387 (PMC13394561; doi:10.1113/EP093387)
Supplement: Supplementary file 1 — Table S1. Table of all significant DMLs identified in the severe intensity domain trial. [file EPH-111-3648-s001.docx]

# **Title: Acute capillary blood DNA methylation responses to swimming exercise in high-performance male and female swimmers**

Goldsmith, C.D.^1, 2^; Lawler, N.G.^3, 4^; Pyne, D.B.^5^; Kozlovskaia, M.^5^; McGibbon, K.^6^; Mitchell, L.J.G.^7^; Govus, A.D.^8^

# **Institutional affiliations:**

1. Translational Health Research Institute, Western Sydney University, Parramatta, NSW, Australia
2. School of Science, Western Sydney University, Parramatta, NSW, Australia
3. Australian National Phenome Centre, Health Futures Institute, Murdoch University, Perth, Western Australia, Australia
4. Centre for Computational and Systems Medicine, Health Futures Institute, Murdoch University, Perth, WA, Australia
5. Research Institute Sport and Exercise, University of Canberra, Canberra, Australia, Australian Capital Territory, Australia
6. Performance Science Unit, Queensland Academy of Sport, Nathan, Queensland, Australia
7. Victorian Institute of Sport, Albert Park, Victoria, Australia
8. Discipline of Sport and Exercise Science, La Trobe University, Bundoora, Victoria, Australia

# **Running title:** DNA methylation profiling of different swimming exercise intensities

**Table of Contents:** Endocrinology and metabolism

# **Co-Correspondence:**

Dr Andrew Govus/ Dr Chloe Goldsmith

**E:** [a.govus@latrobe.edu.au/](mailto:a.govus@latrobe.edu.au/) [C.Goldsmith@westernsydney.edu.au](mailto:C.Goldsmith@westernsydney.edu.au)

**ORCID:** 0000-0001-6224-0454

***Supplementary Table 1:*** *Table of all significant DMLs identified in the severe intensity domain trial.*

|  | **chr** | **start** | **end** | **strand** | **pvalue** | **qvalue** | **meth.diff** |
| --- | --- | --- | --- | --- | --- | --- | --- |
|  | chr1 | 1492864 | 1492864 | * | 1.99584884487142e-05 | 0.0487519477830495 | 2.83687943262412 |
|  | chr1 | 1523741 | 1523741 | * | 1.07438467183994e-06 | 0.0187079686720523 | -5.34591194968553 |
|  | chr1 | 1620637 | 1620637 | * | 1.07383588577324e-05 | 0.0399184503837769 | -4.98360180845671 |
|  | chr1 | 1623921 | 1623921 | * | 1.87953169590313e-06 | 0.0236445123895326 | -3.07951161405599 |
|  | chr1 | 1909409 | 1909409 | * | 6.31877457159864e-06 | 0.033269535448598 | 3.77358490566038 |
|  | chr1 | 2087335 | 2087335 | * | 3.56628213539009e-07 | 0.0115642986305898 | -8.74049517194201 |
|  | chr1 | 2265103 | 2265103 | * | 2.57319899882754e-06 | 0.0268072572724778 | -4.0506329113924 |
|  | chr1 | 2274011 | 2274011 | * | 1.05065317716384e-05 | 0.0398330233830921 | -5.01457426334573 |
|  | chr1 | 3065194 | 3065194 | * | 7.3141068339049e-06 | 0.0355183226808174 | 8.38328792007266 |
|  | chr1 | 3405502 | 3405502 | * | 6.77844646963146e-06 | 0.0343542095460106 | -7.82574095582226 |
|  | chr1 | 3595743 | 3595743 | * | 4.42832184358317e-06 | 0.030682714293622 | 11.4921223354958 |
|  | chr1 | 7639221 | 7639221 | * | 2.10074107891919e-05 | 0.0497198911538814 | -4.81215465966028 |
|  | chr1 | 9730374 | 9730374 | * | 1.71269922253849e-05 | 0.0468130072138101 | -7.86769756938862 |
|  | chr1 | 11719540 | 11719540 | * | 5.09964917186287e-06 | 0.0310952225336125 | 2.94906166219839 |
|  | chr1 | 12143788 | 12143788 | * | 2.75654248204385e-06 | 0.0274210684298071 | 6.39077913171915 |
|  | chr1 | 14599029 | 14599029 | * | 8.46675656484509e-06 | 0.0372521086012845 | -5.34853946969442 |
|  | chr1 | 16162819 | 16162819 | * | 8.66328843313117e-06 | 0.0372521086012845 | 11.380964643185 |
|  | chr1 | 16227779 | 16227779 | * | 2.267685076353e-06 | 0.0253583571596421 | -9.11244760342628 |
|  | chr1 | 17311825 | 17311825 | * | 1.34757672543747e-07 | 0.00890507499086869 | 5.94873181872821 |
|  | chr1 | 17603475 | 17603475 | * | 1.53988526979329e-05 | 0.045146389983925 | 3.40136054421769 |
|  | chr1 | 20581110 | 20581110 | * | 1.97988177243161e-05 | 0.0486224437851043 | 4.24310900304278 |
|  | chr1 | 21564110 | 21564110 | * | 6.7778455753661e-06 | 0.0343542095460106 | -8.68725868725868 |
|  | chr1 | 21855621 | 21855621 | * | 4.31198003785986e-06 | 0.0306440995731637 | -3.8266165717559 |
|  | chr1 | 23595542 | 23595542 | * | 2.08259181972677e-05 | 0.0494343218254836 | -9.48979591836735 |
|  | chr1 | 23903044 | 23903044 | * | 6.67863688024528e-07 | 0.0146676223163947 | -10.7784498586377 |
|  | chr1 | 24686026 | 24686026 | * | 1.85774681799301e-05 | 0.0474217984655563 | 7.27841401869798 |
|  | chr1 | 24737167 | 24737167 | * | 1.54081851726798e-05 | 0.045146389983925 | -3.25 |
|  | chr1 | 25592966 | 25592966 | * | 4.497363292856e-06 | 0.030682714293622 | 3.16196344611214 |
|  | chr1 | 26049429 | 26049429 | * | 1.51798123178896e-05 | 0.0451189213522599 | -5.02283105022832 |
|  | chr1 | 27489850 | 27489850 | * | 2.12600902272282e-06 | 0.0251589637253945 | -3.93358438890791 |
|  | chr1 | 28485683 | 28485683 | * | 1.56017488356296e-05 | 0.0454391445067938 | 4.53302743523564 |
|  | chr1 | 31635300 | 31635300 | * | 4.26015208251082e-06 | 0.0305450648941394 | -5.15021459227468 |
|  | chr1 | 33329008 | 33329008 | * | 1.94732423503053e-06 | 0.0238550350166895 | -3.87678692988427 |
|  | chr1 | 35522784 | 35522784 | * | 1.25592774901957e-05 | 0.0428806450960843 | 4.11859620814844 |
|  | chr1 | 36149608 | 36149608 | * | 2.03143248777146e-05 | 0.0488880499261265 | -3.2967032967033 |
|  | chr1 | 40374265 | 40374265 | * | 1.85114405330199e-05 | 0.047387114739599 | 9.35949003834121 |
|  | chr1 | 40523047 | 40523047 | * | 1.14435452004563e-05 | 0.0406067954961554 | -3.59795950900685 |
|  | chr1 | 41483655 | 41483655 | * | 1.57940421394493e-07 | 0.00890507499086869 | -9.13354472384043 |
|  | chr1 | 41661623 | 41661623 | * | 2.69864266219489e-06 | 0.0274112604430449 | -3.23232323232323 |
|  | chr1 | 41896644 | 41896644 | * | 1.51463183015453e-05 | 0.0451189213522599 | -9.26136220253867 |
|  | chr1 | 44030838 | 44030838 | * | 1.58925623867281e-06 | 0.0223141858609182 | -3.08529945553539 |
|  | chr1 | 47443945 | 47443945 | * | 4.64852971673756e-06 | 0.0308169634740055 | 7.1904030429376 |
|  | chr1 | 47726111 | 47726111 | * | 1.45750791952452e-05 | 0.0448462162021566 | 4.93475452196382 |
|  | chr1 | 53088931 | 53088931 | * | 1.84254834670835e-05 | 0.0473326884493546 | 4.96665305566898 |
|  | chr1 | 53093431 | 53093431 | * | 9.12444585270366e-07 | 0.0181038858912785 | 13.2003028336578 |
|  | chr1 | 56193326 | 56193326 | * | 1.6529773228289e-06 | 0.0224030206914996 | -8.25421396628827 |
|  | chr1 | 58546990 | 58546990 | * | 1.36269734504653e-05 | 0.0440049293460441 | -2.9023746701847 |
|  | chr1 | 75724839 | 75724839 | * | 2.52164386952147e-07 | 0.00987832060906468 | 1.97628458498024 |
|  | chr1 | 77682397 | 77682397 | * | 1.5305836838605e-05 | 0.045130930916365 | -2.35081374321881 |
|  | chr1 | 79507209 | 79507209 | * | 4.16127105161807e-06 | 0.0304426130789399 | 10.1684023512068 |
|  | chr1 | 89610025 | 89610025 | * | 9.13955863740463e-06 | 0.0380209070549651 | 10.3623234173351 |
|  | chr1 | 89948049 | 89948049 | * | 9.49419984362582e-07 | 0.0183688805422173 | -4.52961672473867 |
|  | chr1 | 93157951 | 93157951 | * | 1.79883963116507e-05 | 0.0472793408087894 | -4.55149590328005 |
|  | chr1 | 94542228 | 94542228 | * | 1.70145240455213e-05 | 0.0468130072138101 | -4.08632508796133 |
|  | chr1 | 100539440 | 100539440 | * | 7.93372259952829e-06 | 0.0366300873979608 | -2.76008492569002 |
|  | chr1 | 101933020 | 101933020 | * | 3.53731577876144e-07 | 0.0115642986305898 | -5.93103215802602 |
|  | chr1 | 111982059 | 111982059 | * | 1.19894578676281e-05 | 0.0414577086233507 | -4.97972116603296 |
|  | chr1 | 114537595 | 114537595 | * | 9.9641938960975e-06 | 0.03864959922597 | 2.48091603053435 |
|  | chr1 | 146735407 | 146735407 | * | 6.962553612596e-06 | 0.034843158469973 | -5.47063555913113 |
|  | chr1 | 150229774 | 150229774 | * | 5.54474733840468e-06 | 0.0321066158102395 | 3.56580990519778 |
|  | chr1 | 152312524 | 152312524 | * | 4.81030200747723e-06 | 0.0310406905817438 | -7.10695285268758 |
|  | chr1 | 154280050 | 154280050 | * | 1.75905223687829e-05 | 0.0471534938979854 | 3.78007655851258 |
|  | chr1 | 158348277 | 158348277 | * | 4.12747650177371e-06 | 0.0303068913296594 | 8.4193430637627 |
|  | chr1 | 164576351 | 164576351 | * | 1.84058239437934e-07 | 0.00934778881361428 | 7.2 |
|  | chr1 | 165829008 | 165829008 | * | 3.82390952866235e-06 | 0.0298414787713016 | 4.59770114942529 |
|  | chr1 | 165835955 | 165835955 | * | 2.03370459812722e-05 | 0.0488880499261265 | 3.14086541372243 |
|  | chr1 | 169521769 | 169521769 | * | 1.40860927168425e-07 | 0.00890507499086869 | 5.04807692307693 |
|  | chr1 | 171538996 | 171538996 | * | 1.53120408567864e-05 | 0.045130930916365 | 4.36363636363636 |
|  | chr1 | 171615683 | 171615683 | * | 1.59723510573588e-05 | 0.0455833711863926 | 4.68935779425941 |
|  | chr1 | 178499718 | 178499718 | * | 7.2233210719592e-06 | 0.0351971330669854 | -3.48824736185789 |
|  | chr1 | 179575828 | 179575828 | * | 7.15091166106283e-06 | 0.0351458946188086 | -2.48565965583174 |
|  | chr1 | 184965171 | 184965171 | * | 1.23192312258179e-05 | 0.0424175143165775 | -4.09556313993175 |
|  | chr1 | 193121983 | 193121983 | * | 3.63118312366654e-06 | 0.0293113670780731 | 11.625 |
|  | chr1 | 198195618 | 198195618 | * | 3.10451020467595e-06 | 0.0284515755480276 | 2.71844660194175 |
|  | chr1 | 204074946 | 204074946 | * | 2.92097051896512e-07 | 0.0101861931984575 | -3.20121951219512 |
|  | chr1 | 205380613 | 205380613 | * | 2.06744558627136e-06 | 0.0247057923342734 | 4.66846643177526 |
|  | chr1 | 218394198 | 218394198 | * | 1.16900439821998e-06 | 0.0195190265746677 | -7.2463768115942 |
|  | chr1 | 223847257 | 223847257 | * | 5.76785473455831e-06 | 0.0326804094071279 | -5.14186962462824 |
|  | chr1 | 228140809 | 228140809 | * | 1.41935285095371e-05 | 0.0445180990564497 | 2.64765784114053 |
|  | chr1 | 229317501 | 229317501 | * | 2.58542329413873e-06 | 0.02681999305886 | -1.93861066235864 |
|  | chr1 | 234772515 | 234772515 | * | 6.89609872140277e-06 | 0.0346622378005334 | -2.21843003412969 |
|  | chr1 | 235002332 | 235002332 | * | 1.46870200742547e-05 | 0.0449484016985218 | -3.55792400370714 |
|  | chr1 | 241848146 | 241848146 | * | 1.12222788744787e-05 | 0.0401362202903679 | -2.08333333333333 |
|  | chr1 | 244725926 | 244725926 | * | 1.14901915054202e-05 | 0.0406539652082238 | 3.19117214914176 |
|  | chr1 | 244832123 | 244832123 | * | 1.50296787643595e-05 | 0.0451189213522599 | -4.0650406504065 |
|  | chr1 | 248916196 | 248916196 | * | 1.69094521464016e-06 | 0.0224030206914996 | 5.69790588422645 |
|  | chr10 | 627542 | 627542 | * | 6.25513632854892e-06 | 0.0330331766726775 | -5.8862020614499 |
|  | chr10 | 2207803 | 2207803 | * | 1.48138064943977e-05 | 0.0449484016985218 | 4.01849058961065 |
|  | chr10 | 8034095 | 8034095 | * | 1.23843030215107e-05 | 0.0425712226932395 | -9.69836614997905 |
|  | chr10 | 18140249 | 18140249 | * | 6.60473850581654e-06 | 0.0339097303865037 | 3.59212954539547 |
|  | chr10 | 23172681 | 23172681 | * | 1.34046351178507e-05 | 0.0440049293460441 | -3.34285950960901 |
|  | chr10 | 29170917 | 29170917 | * | 1.37745585004346e-05 | 0.0441833825925084 | 3.08875252563048 |
|  | chr10 | 42358600 | 42358600 | * | 1.68637219824407e-06 | 0.0224030206914996 | 7.60115298387958 |
|  | chr10 | 42782843 | 42782843 | * | 1.09936834774221e-05 | 0.0401362202903679 | 3.19488817891374 |
|  | chr10 | 45554925 | 45554925 | * | 5.19229505372275e-06 | 0.0312632216132437 | -3.0690537084399 |
|  | chr10 | 52314263 | 52314263 | * | 1.17812310067081e-05 | 0.0412644725373489 | 5.95238095238095 |
|  | chr10 | 70609454 | 70609454 | * | 1.53153898356362e-05 | 0.045130930916365 | -3.46965010441703 |
|  | chr10 | 72297210 | 72297210 | * | 3.31242348047308e-06 | 0.0290437810662865 | -4.74452554744525 |
|  | chr10 | 72403115 | 72403115 | * | 1.31308637485536e-05 | 0.0437895804250019 | 2.66075388026608 |
|  | chr10 | 72640121 | 72640121 | * | 4.97166875721564e-06 | 0.0310952225336125 | -5.3555999567988 |
|  | chr10 | 73544186 | 73544186 | * | 1.841786434762e-05 | 0.0473326884493546 | -4.51100244498778 |
|  | chr10 | 73942548 | 73942548 | * | 2.0003067955407e-06 | 0.0241391516940067 | -4.5751633986928 |
|  | chr10 | 77567339 | 77567339 | * | 5.69248728839463e-06 | 0.0326306195840953 | 3.77422284687647 |
|  | chr10 | 78571469 | 78571469 | * | 9.04961670373822e-08 | 0.00890507499086869 | 2.59917920656635 |
|  | chr10 | 86910054 | 86910054 | * | 1.79068837468995e-05 | 0.0472518712534723 | 3.92962743938499 |
|  | chr10 | 89837968 | 89837968 | * | 4.00823746886599e-06 | 0.0300127101341861 | -5.40178571428571 |
|  | chr10 | 92250321 | 92250321 | * | 1.74334159930144e-05 | 0.0469912079857756 | -4.49005154887507 |
|  | chr10 | 94772907 | 94772907 | * | 1.64775308519072e-05 | 0.0460649404991101 | -9.49771689497717 |
|  | chr10 | 96056201 | 96056201 | * | 1.52094914103803e-05 | 0.0451189213522599 | -4.51905269655984 |
|  | chr10 | 97169753 | 97169753 | * | 4.54374283921945e-06 | 0.030682714293622 | -7.18526363082057 |
|  | chr10 | 97641007 | 97641007 | * | 1.02291283469004e-05 | 0.0392076628888773 | -2.26843100189036 |
|  | chr10 | 99970748 | 99970748 | * | 5.05876593630559e-06 | 0.0310952225336125 | 3.7982676582771 |
|  | chr10 | 101067680 | 101067680 | * | 5.86061881334767e-07 | 0.0144075326810445 | 5.4054054054054 |
|  | chr10 | 101146394 | 101146394 | * | 1.5923895260561e-05 | 0.0455833711863926 | -11.2829039573937 |
|  | chr10 | 108110366 | 108110366 | * | 1.6791780869971e-06 | 0.0224030206914996 | -6.2488174077578 |
|  | chr10 | 108567070 | 108567070 | * | 8.66441116429947e-06 | 0.0372521086012845 | -7.32658149509338 |
|  | chr10 | 116741519 | 116741519 | * | 6.017502081056e-06 | 0.0329166188805524 | 4.37929566328082 |
|  | chr10 | 121626628 | 121626628 | * | 2.8737841154036e-06 | 0.0274211735986337 | -4.85546522131888 |
|  | chr10 | 125079338 | 125079338 | * | 2.0499240192653e-05 | 0.0490792825012072 | -3.35751150788115 |
|  | chr10 | 129467322 | 129467322 | * | 1.33713392901188e-05 | 0.0440049293460441 | 10.9137186343069 |
|  | chr10 | 129808406 | 129808406 | * | 1.43060716558053e-05 | 0.0445180990564497 | -4.66245466245466 |
|  | chr10 | 129959741 | 129959741 | * | 4.83828154590194e-06 | 0.0310406905817438 | -6.20241554968673 |
|  | chr10 | 129973542 | 129973542 | * | 5.47737198962661e-06 | 0.0319441120660669 | 12.3735651708157 |
|  | chr10 | 130299547 | 130299547 | * | 1.61101226915352e-06 | 0.0223141858609182 | -4.26829268292683 |
|  | chr10 | 131769589 | 131769589 | * | 2.02007885502391e-05 | 0.0487725967640558 | 3.0304939516129 |
|  | chr10 | 132416425 | 132416425 | * | 1.41898870459524e-08 | 0.00724041545745036 | -5 |
|  | chr10 | 132714400 | 132714400 | * | 8.91417613612927e-06 | 0.0375965711582794 | 5.6615072784227 |
|  | chr10 | 132776335 | 132776335 | * | 6.11085315875106e-06 | 0.0329578049447931 | 6.76963341135706 |
|  | chr11 | 294027 | 294027 | * | 3.29932626627861e-06 | 0.0290437810662865 | -3.96114219643632 |
|  | chr11 | 400269 | 400269 | * | 1.6057344027967e-05 | 0.0455833711863926 | -13.2264212947749 |
|  | chr11 | 460587 | 460587 | * | 1.93276204377352e-05 | 0.0482366656690467 | -8.8492770845712 |
|  | chr11 | 2361070 | 2361070 | * | 1.70678834095172e-05 | 0.0468130072138101 | 8.94012944983819 |
|  | chr11 | 2405470 | 2405470 | * | 1.63458781907132e-07 | 0.00890507499086869 | -6.10159614396903 |
|  | chr11 | 2521459 | 2521459 | * | 1.08760259473094e-05 | 0.0401362202903679 | 8.12525967324729 |
|  | chr11 | 3591540 | 3591540 | * | 1.17119657682468e-05 | 0.0411400854963229 | 2.37623762376238 |
|  | chr11 | 6234810 | 6234810 | * | 5.14952647890105e-06 | 0.0312632216132437 | -2.40740740740741 |
|  | chr11 | 9405455 | 9405455 | * | 3.48980265974025e-06 | 0.0293113670780731 | 5.18637726609996 |
|  | chr11 | 18394918 | 18394918 | * | 2.43270644453009e-06 | 0.0262633459760735 | -9.32011906372615 |
|  | chr11 | 30791059 | 30791059 | * | 1.22122625374289e-05 | 0.0421683198201396 | -4.45888572280784 |
|  | chr11 | 31532917 | 31532917 | * | 1.26428148833422e-05 | 0.0430140569446174 | -4.26055426055426 |
|  | chr11 | 31532929 | 31532929 | * | 2.9772228982836e-06 | 0.0279786966436556 | -5.09806440561836 |
|  | chr11 | 45226916 | 45226916 | * | 1.91627056630551e-05 | 0.0480849487724047 | 2.37226277372263 |
|  | chr11 | 45923382 | 45923382 | * | 1.95992315458181e-05 | 0.0484431948255683 | -11.0298502227367 |
|  | chr11 | 59553519 | 59553519 | * | 5.71230519138263e-06 | 0.0326306195840953 | -2.70758122743683 |
|  | chr11 | 62318324 | 62318324 | * | 4.32424648847531e-06 | 0.0306440995731637 | 3.96485731358711 |
|  | chr11 | 62547131 | 62547131 | * | 4.25633749784763e-06 | 0.0305450648941394 | -6.22259448853056 |
|  | chr11 | 62881341 | 62881341 | * | 1.45591530058918e-05 | 0.0448462162021566 | 2.86396181384248 |
|  | chr11 | 63552415 | 63552415 | * | 5.50836882131782e-06 | 0.0320482156610258 | -5.80977546626401 |
|  | chr11 | 64879282 | 64879282 | * | 1.12286095467236e-05 | 0.0401362202903679 | -2.64650283553875 |
|  | chr11 | 65041038 | 65041038 | * | 9.04051911898703e-06 | 0.037972189934139 | -3.38164251207729 |
|  | chr11 | 65283716 | 65283716 | * | 8.4540610641032e-07 | 0.0177408848071462 | -4.57488577955455 |
|  | chr11 | 65787407 | 65787407 | * | 6.65206299327803e-06 | 0.033925270769622 | 3.21888412017167 |
|  | chr11 | 66518208 | 66518208 | * | 2.74808378825366e-06 | 0.0274210684298071 | 3.17796610169492 |
|  | chr11 | 66710724 | 66710724 | * | 9.92804425708098e-06 | 0.038600331076739 | 6.58130760190512 |
|  | chr11 | 66986012 | 66986012 | * | 1.4661622966531e-07 | 0.00890507499086869 | -6.29139072847682 |
|  | chr11 | 67189611 | 67189611 | * | 3.99665086320325e-06 | 0.0300127101341861 | 3.98773006134969 |
|  | chr11 | 69166663 | 69166663 | * | 9.04996523550292e-06 | 0.037972189934139 | -9.06622047750821 |
|  | chr11 | 69470973 | 69470973 | * | 9.23699632627297e-06 | 0.0380630407857723 | -7.53631961259079 |
|  | chr11 | 69765829 | 69765829 | * | 1.10913629859703e-05 | 0.0401362202903679 | -9.4439760882894 |
|  | chr11 | 71261761 | 71261761 | * | 3.72751867217708e-06 | 0.0294073719213501 | -7.91095890410959 |
|  | chr11 | 71407468 | 71407468 | * | 1.56199377535371e-05 | 0.0454391445067938 | -4.94090394587906 |
|  | chr11 | 71463561 | 71463561 | * | 1.86550259524046e-05 | 0.0475202575509978 | -6.3994506678156 |
|  | chr11 | 73264442 | 73264442 | * | 8.8400179813251e-06 | 0.0374335718834972 | -9.9080919447451 |
|  | chr11 | 73377236 | 73377236 | * | 4.30021388623996e-06 | 0.0306440995731637 | -3.41463414634146 |
|  | chr11 | 76160193 | 76160193 | * | 4.85620258833538e-06 | 0.0310406905817438 | -8.21648869997926 |
|  | chr11 | 93741182 | 93741182 | * | 1.51937582375611e-05 | 0.0451189213522599 | -4.77815699658703 |
|  | chr11 | 95632190 | 95632190 | * | 4.74972878987252e-06 | 0.0310406905817438 | -2.22222222222223 |
|  | chr11 | 101127489 | 101127489 | * | 1.16339161025682e-05 | 0.040924893599352 | -2.91941133228592 |
|  | chr11 | 108696269 | 108696269 | * | 8.45474789014348e-06 | 0.0372521086012845 | 2.79069767441861 |
|  | chr11 | 108696368 | 108696368 | * | 3.43372359781219e-06 | 0.0292680754535727 | 7.07780379911527 |
|  | chr11 | 109422382 | 109422382 | * | 6.66783182235828e-07 | 0.0146676223163947 | 5.51470588235294 |
|  | chr11 | 117328253 | 117328253 | * | 2.89705124222921e-07 | 0.0101861931984575 | -7.17388029724844 |
|  | chr11 | 119422096 | 119422096 | * | 1.40489234282804e-06 | 0.0210111734066504 | -7.8961874138723 |
|  | chr11 | 123356671 | 123356671 | * | 7.05997002577029e-06 | 0.0349354795451427 | -5.31541142166276 |
|  | chr11 | 123592960 | 123592960 | * | 1.94220771647513e-05 | 0.0484073443519774 | -4.18410041841004 |
|  | chr11 | 126531107 | 126531107 | * | 4.01822073413159e-06 | 0.0300127101341861 | -7.22460006678202 |
|  | chr11 | 126776139 | 126776139 | * | 1.34475781005919e-05 | 0.0440049293460441 | 5.87956989247311 |
|  | chr11 | 127577172 | 127577172 | * | 1.86152840254354e-05 | 0.0474686237678807 | 5.70646221248632 |
|  | chr11 | 129279132 | 129279132 | * | 1.4414845528794e-05 | 0.0446291578551067 | -4.31654676258993 |
|  | chr11 | 131911614 | 131911614 | * | 9.72010751485699e-06 | 0.0381570098837087 | 3.87373998449212 |
|  | chr11 | 133931515 | 133931515 | * | 1.06188728586727e-05 | 0.0399184503837769 | 4.84677471800912 |
|  | chr11 | 134276728 | 134276728 | * | 1.6832979121045e-05 | 0.0465779040949594 | -2.27272727272727 |
|  | chr11 | 134501031 | 134501031 | * | 4.83685238190738e-06 | 0.0310406905817438 | -5.08135275577136 |
|  | chr12 | 140080 | 140080 | * | 6.49932619673314e-07 | 0.0146149559616363 | 6.03767923355553 |
|  | chr12 | 991406 | 991406 | * | 5.88277266751454e-06 | 0.0328824397860396 | -3.24675324675325 |
|  | chr12 | 1001278 | 1001278 | * | 5.09912135866311e-07 | 0.0138117180710717 | -4.13386243866706 |
|  | chr12 | 1797367 | 1797367 | * | 2.01784338758112e-05 | 0.0487725967640558 | 2.66075388026608 |
|  | chr12 | 2753066 | 2753066 | * | 1.59491625589541e-07 | 0.00890507499086869 | -15.0283687943262 |
|  | chr12 | 3227713 | 3227713 | * | 1.68204148783093e-05 | 0.0465779040949594 | -5.88383838383839 |
|  | chr12 | 4126462 | 4126462 | * | 2.00886359249482e-05 | 0.0487725967640558 | -7.04687897499943 |
|  | chr12 | 6088351 | 6088351 | * | 1.96134983738644e-05 | 0.0484431948255683 | -4.29030804365925 |
|  | chr12 | 6123176 | 6123176 | * | 1.51508029390123e-05 | 0.0451189213522599 | -4.77380952380952 |
|  | chr12 | 7708861 | 7708861 | * | 5.72288200376417e-07 | 0.0144075326810445 | 3.33333333333333 |
|  | chr12 | 8005365 | 8005365 | * | 5.07284764068806e-06 | 0.0310952225336125 | 5.52314194412946 |
|  | chr12 | 11608613 | 11608613 | * | 1.83213026493682e-05 | 0.0473326884493546 | 12.3456157433506 |
|  | chr12 | 12310308 | 12310308 | * | 1.74642500580908e-05 | 0.0469912079857756 | 3.58155682730644 |
|  | chr12 | 31362461 | 31362461 | * | 1.16278056366926e-05 | 0.040924893599352 | -13.7622139156666 |
|  | chr12 | 32897077 | 32897077 | * | 2.61308091391203e-06 | 0.0269920407477894 | 2.91666666666667 |
|  | chr12 | 34338434 | 34338434 | * | 9.70097130807284e-06 | 0.0381570098837087 | 14.7366218371433 |
|  | chr12 | 42008243 | 42008243 | * | 2.78032772959238e-06 | 0.0274210684298071 | 5.75582213113107 |
|  | chr12 | 47816172 | 47816172 | * | 2.40435422309765e-07 | 0.00987832060906468 | -4.14937759336099 |
|  | chr12 | 48134470 | 48134470 | * | 1.79100219126273e-05 | 0.0472518712534723 | -5.51482225231816 |
|  | chr12 | 48995592 | 48995592 | * | 1.61475182755321e-05 | 0.0455833711863926 | 5.76131687242798 |
|  | chr12 | 49077690 | 49077690 | * | 1.2993309991596e-05 | 0.0435094192554022 | 3.73411808669656 |
|  | chr12 | 49539160 | 49539160 | * | 1.98611720057073e-05 | 0.0486605101652434 | 3.97727272727273 |
|  | chr12 | 49967791 | 49967791 | * | 6.53475249147938e-07 | 0.0146149559616363 | 15.5796083615632 |
|  | chr12 | 51218380 | 51218380 | * | 5.1022154620245e-06 | 0.0310952225336125 | 9.28193298435513 |
|  | chr12 | 51721792 | 51721792 | * | 1.05299565192658e-05 | 0.0398574319718915 | -5.23023793567707 |
|  | chr12 | 52899938 | 52899938 | * | 5.60689826236413e-07 | 0.0144075326810445 | 4.25872538014084 |
|  | chr12 | 53626280 | 53626280 | * | 6.06640652049111e-07 | 0.0144075326810445 | -11.0064100670546 |
|  | chr12 | 54254713 | 54254713 | * | 2.46092514065226e-06 | 0.0262633459760735 | -5.34351145038168 |
|  | chr12 | 55767740 | 55767740 | * | 1.96522336085943e-05 | 0.0484889732288157 | 5.66678252809639 |
|  | chr12 | 57236367 | 57236367 | * | 3.171710697355e-07 | 0.010738804510027 | -6.72665916760405 |
|  | chr12 | 57459954 | 57459954 | * | 9.80814379891566e-06 | 0.0383789990472042 | -2.96127562642369 |
|  | chr12 | 57464720 | 57464720 | * | 9.71074873725416e-06 | 0.0381570098837087 | -4.09460683478511 |
|  | chr12 | 62624863 | 62624863 | * | 6.04081032868428e-08 | 0.00866245804981213 | -8.9787113255748 |
|  | chr12 | 71686278 | 71686278 | * | 6.04215861912119e-06 | 0.0329166188805524 | 2.2181146025878 |
|  | chr12 | 76483823 | 76483823 | * | 1.93320085055426e-05 | 0.0482366656690467 | -2.85729264968019 |
|  | chr12 | 77976828 | 77976828 | * | 1.78566058833405e-05 | 0.0472518712534723 | -8.69323093731603 |
|  | chr12 | 78865145 | 78865145 | * | 2.65639259609018e-07 | 0.010118290712003 | 8.3499104386257 |
|  | chr12 | 83325610 | 83325610 | * | 4.69932384004917e-06 | 0.0308785096211395 | -5.74234092694422 |
|  | chr12 | 93421358 | 93421358 | * | 6.53476991572537e-06 | 0.0338141303613569 | -8.38219875979284 |
|  | chr12 | 96355702 | 96355702 | * | 2.03551304731179e-05 | 0.0488880499261265 | -3.43347639484979 |
|  | chr12 | 98895415 | 98895415 | * | 3.485513238368e-06 | 0.0293113670780731 | -5.45454545454545 |
|  | chr12 | 102060869 | 102060869 | * | 4.05236116658404e-06 | 0.0300127101341861 | 3.25814536340853 |
|  | chr12 | 107318259 | 107318259 | * | 1.78423341339592e-05 | 0.0472518712534723 | -2.321083172147 |
|  | chr12 | 111459686 | 111459686 | * | 3.5822430289545e-06 | 0.0293113670780731 | 3.47222222222222 |
|  | chr12 | 112109939 | 112109939 | * | 7.43125821176927e-06 | 0.035801951592892 | -7.44385393451055 |
|  | chr12 | 113476325 | 113476325 | * | 6.07954954964469e-07 | 0.0144075326810445 | 8.73362445414847 |
|  | chr12 | 117082419 | 117082419 | * | 9.13772932087305e-06 | 0.0380209070549651 | 3.00062098542625 |
|  | chr12 | 118136815 | 118136815 | * | 1.62045737620629e-05 | 0.0455833711863926 | 2.63157894736842 |
|  | chr12 | 118371644 | 118371644 | * | 1.6152791197393e-05 | 0.0455833711863926 | -2.27272727272727 |
|  | chr12 | 120738394 | 120738394 | * | 6.20882335693568e-06 | 0.0330331766726775 | -3.72199891028343 |
|  | chr12 | 121892528 | 121892528 | * | 1.72605194795913e-05 | 0.046856774719373 | -4.4520778608473 |
|  | chr12 | 123857500 | 123857500 | * | 7.49265303362465e-06 | 0.0360079652024686 | -3.19019952979407 |
|  | chr12 | 128932943 | 128932943 | * | 1.604702105031e-05 | 0.0455833711863926 | -4.39045277697799 |
|  | chr12 | 131622494 | 131622494 | * | 1.18035825549318e-05 | 0.0412834448894215 | -13.0687347931873 |
|  | chr12 | 131901902 | 131901902 | * | 9.86271222610641e-06 | 0.0384690277375714 | 8.4280303030303 |
|  | chr12 | 132060559 | 132060559 | * | 1.47745867905719e-05 | 0.0449484016985218 | -7.31773372332358 |
|  | chr12 | 132369368 | 132369368 | * | 1.28582097559786e-06 | 0.0201016113850057 | -5.36385164610152 |
|  | chr13 | 18665743 | 18665743 | * | 1.14257791097479e-07 | 0.00890507499086869 | 10.6288900103333 |
|  | chr13 | 20946220 | 20946220 | * | 2.56221811121694e-06 | 0.0268072572724778 | -9.17261917607199 |
|  | chr13 | 21818226 | 21818226 | * | 1.7796839008112e-05 | 0.0472518712534723 | -10.8254269449715 |
|  | chr13 | 25018903 | 25018903 | * | 9.45335933041593e-06 | 0.0380630407857723 | 5.2779916474184 |
|  | chr13 | 25953849 | 25953849 | * | 1.46807422303936e-05 | 0.0449484016985218 | -4.59719142645971 |
|  | chr13 | 26235177 | 26235177 | * | 1.05883056032748e-06 | 0.0187043339204075 | 4.88327822073679 |
|  | chr13 | 26747012 | 26747012 | * | 1.60251539061648e-06 | 0.0223141858609182 | -4.67332686066523 |
|  | chr13 | 27303703 | 27303703 | * | 1.3535974185197e-05 | 0.0440049293460441 | -4.1010757681294 |
|  | chr13 | 35475922 | 35475922 | * | 2.19139285851639e-06 | 0.0251778165859805 | -5.5784574844069 |
|  | chr13 | 44159152 | 44159152 | * | 8.16267758805401e-08 | 0.00890507499086869 | -5.81709956709956 |
|  | chr13 | 49997185 | 49997185 | * | 1.3595659788781e-05 | 0.0440049293460441 | 3.60824742268041 |
|  | chr13 | 52913205 | 52913205 | * | 1.95238869066362e-05 | 0.0484431948255683 | -5.11800296509997 |
|  | chr13 | 54082516 | 54082516 | * | 1.63678148393317e-07 | 0.00890507499086869 | -5.35474287331117 |
|  | chr13 | 57129399 | 57129399 | * | 7.32987259767806e-06 | 0.0355241182523117 | -8.47103646547882 |
|  | chr13 | 58290221 | 58290221 | * | 1.29894071637573e-05 | 0.0435094192554022 | 5.10768530868411 |
|  | chr13 | 67231018 | 67231018 | * | 6.44171644378369e-06 | 0.033554488025128 | -3.34190231362468 |
|  | chr13 | 78596011 | 78596011 | * | 4.5217272912907e-06 | 0.030682714293622 | -4.8951048951049 |
|  | chr13 | 79405693 | 79405693 | * | 1.2303332284972e-06 | 0.0198485517980604 | -8.86844963345099 |
|  | chr13 | 87749433 | 87749433 | * | 1.60258868853273e-05 | 0.0455833711863926 | -5.68612690590807 |
|  | chr13 | 98431251 | 98431251 | * | 7.02705663916794e-06 | 0.0348888639265664 | -7.37651555455996 |
|  | chr13 | 100494412 | 100494412 | * | 3.37480374109106e-06 | 0.0290437810662865 | 5.33980582524272 |
|  | chr13 | 108543427 | 108543427 | * | 1.48083880567322e-05 | 0.0449484016985218 | -6.17934446505876 |
|  | chr13 | 113084938 | 113084938 | * | 1.73854553771648e-05 | 0.0469912079857756 | 5.70682672131947 |
|  | chr13 | 113089967 | 113089967 | * | 2.71846799744402e-06 | 0.0274210684298071 | -5.3763440860215 |
|  | chr13 | 113522592 | 113522592 | * | 3.5845753014936e-06 | 0.0293113670780731 | -3.94736842105263 |
|  | chr13 | 113834850 | 113834850 | * | 1.43368975302519e-06 | 0.0211819580212682 | -6.39253393665159 |
|  | chr14 | 20422481 | 20422481 | * | 2.16486764327583e-06 | 0.0251778165859805 | 3.95622895622896 |
|  | chr14 | 20729332 | 20729332 | * | 1.90534058862905e-05 | 0.0479835226325341 | 5.80860787122297 |
|  | chr14 | 21419287 | 21419287 | * | 1.71205787311293e-05 | 0.0468130072138101 | -2.16216216216216 |
|  | chr14 | 22846219 | 22846219 | * | 3.5480031374855e-06 | 0.0293113670780731 | 4.51749514023883 |
|  | chr14 | 24271722 | 24271722 | * | 9.52480784002716e-06 | 0.0380645985342247 | 1.91780821917808 |
|  | chr14 | 28766694 | 28766694 | * | 3.12243152809061e-06 | 0.0285086418554748 | -6.12244897959184 |
|  | chr14 | 31110249 | 31110249 | * | 1.97678958306313e-06 | 0.023975035141188 | -2.84237726098191 |
|  | chr14 | 33950767 | 33950767 | * | 1.18634032728571e-05 | 0.0413534866384187 | 6.89655172413793 |
|  | chr14 | 36517289 | 36517289 | * | 1.98436841247199e-05 | 0.0486605101652434 | 2.98165137614679 |
|  | chr14 | 55564358 | 55564358 | * | 1.42509125717918e-05 | 0.0445180990564497 | 4.44632290786138 |
|  | chr14 | 56689415 | 56689415 | * | 1.16238548266225e-05 | 0.040924893599352 | 3.95396514770292 |
|  | chr14 | 64397589 | 64397589 | * | 1.00698592125906e-09 | 0.00245481215309876 | 6.17577197149644 |
|  | chr14 | 67099368 | 67099368 | * | 1.16963995680079e-07 | 0.00890507499086869 | -3.71819960861057 |
|  | chr14 | 67425416 | 67425416 | * | 2.63196076825402e-06 | 0.0270723484622609 | -7.90211867203484 |
|  | chr14 | 67515433 | 67515433 | * | 1.38701983494819e-05 | 0.0443733856598382 | -3.76712328767123 |
|  | chr14 | 72930458 | 72930458 | * | 1.28635430734204e-06 | 0.0201016113850057 | -4.76190476190477 |
|  | chr14 | 73786610 | 73786610 | * | 1.50340856525036e-05 | 0.0451189213522599 | -4.39054470709147 |
|  | chr14 | 75265475 | 75265475 | * | 3.62409053793353e-06 | 0.0293113670780731 | -5.5045871559633 |
|  | chr14 | 77026547 | 77026547 | * | 7.67281281429258e-06 | 0.0361593579075616 | 16.4451611006832 |
|  | chr14 | 93788420 | 93788420 | * | 1.89061670597632e-05 | 0.047909681649983 | -4.71158958001063 |
|  | chr14 | 95482876 | 95482876 | * | 3.08266220888256e-06 | 0.0283579563958269 | -5.36172446927596 |
|  | chr14 | 99733087 | 99733087 | * | 4.98937351966519e-07 | 0.0138117180710717 | 6.5282969375041 |
|  | chr14 | 101030927 | 101030927 | * | 8.85857972003184e-06 | 0.0374335718834972 | -4.20711974110033 |
|  | chr14 | 101035517 | 101035517 | * | 1.82002457152976e-05 | 0.0473326884493546 | -3.98503342051729 |
|  | chr14 | 101198398 | 101198398 | * | 1.43045398918223e-06 | 0.0211819580212682 | -9.03727908804051 |
|  | chr14 | 101202759 | 101202759 | * | 3.06173896033959e-06 | 0.0282721671447522 | 3.75939849624061 |
|  | chr14 | 102674921 | 102674921 | * | 7.60947683430836e-06 | 0.0361593579075616 | 4.1930306490941 |
|  | chr14 | 104137449 | 104137449 | * | 1.53473697523678e-05 | 0.045130930916365 | -8.908989789815 |
|  | chr14 | 104665947 | 104665947 | * | 1.72357140591006e-05 | 0.0468415980941163 | 3.6697247706422 |
|  | chr15 | 22859588 | 22859588 | * | 4.45914508632751e-06 | 0.030682714293622 | 3.98671096345515 |
|  | chr15 | 28107230 | 28107230 | * | 1.6436165046314e-05 | 0.0460020520079604 | 3.74331550802139 |
|  | chr15 | 29029955 | 29029955 | * | 1.36286680499993e-05 | 0.0440049293460441 | -4.2601194426012 |
|  | chr15 | 31453688 | 31453688 | * | 9.26203033181241e-07 | 0.0182087184083438 | 5.73573573573574 |
|  | chr15 | 34437322 | 34437322 | * | 1.11871633600212e-06 | 0.0190712345944889 | -14.2302378551958 |
|  | chr15 | 34446099 | 34446099 | * | 3.62063980182789e-06 | 0.0293113670780731 | -5.7110679651231 |
|  | chr15 | 40272297 | 40272297 | * | 8.60673807958173e-06 | 0.0372521086012845 | -5.34036710507299 |
|  | chr15 | 46343715 | 46343715 | * | 4.04575752423122e-06 | 0.0300127101341861 | -13.9000116175502 |
|  | chr15 | 49237767 | 49237767 | * | 6.61738778720924e-06 | 0.0339097303865037 | -3.50404312668463 |
|  | chr15 | 56540457 | 56540457 | * | 1.86178442751354e-06 | 0.0236445123895326 | -3.09050772626932 |
|  | chr15 | 59945229 | 59945229 | * | 5.46515964334865e-07 | 0.0143256643071847 | -6.38297872340425 |
|  | chr15 | 65376977 | 65376977 | * | 3.81094264932786e-06 | 0.0298414787713016 | -2.63653483992467 |
|  | chr15 | 66497070 | 66497070 | * | 5.781686910433e-06 | 0.0326804094071279 | -8.89988772026306 |
|  | chr15 | 66781395 | 66781395 | * | 1.88695569753361e-05 | 0.0479079694357851 | 2.99532553371978 |
|  | chr15 | 69111604 | 69111604 | * | 1.38222022678676e-06 | 0.0209048346176606 | 9.14582414226083 |
|  | chr15 | 70258698 | 70258698 | * | 7.0035580089962e-06 | 0.034843158469973 | -3.44224430788424 |
|  | chr15 | 70566607 | 70566607 | * | 2.45721643631507e-06 | 0.0262633459760735 | -5.55271659824779 |
|  | chr15 | 73828849 | 73828849 | * | 3.2215903042037e-06 | 0.0287221794777653 | -3.68190555456474 |
|  | chr15 | 75452673 | 75452673 | * | 1.94453626991133e-05 | 0.0484073443519774 | -2.65339966832504 |
|  | chr15 | 75717392 | 75717392 | * | 1.10773993897428e-05 | 0.0401362202903679 | 4.78504080043828 |
|  | chr15 | 77420032 | 77420032 | * | 1.42291185008536e-05 | 0.0445180990564497 | -1.94805194805195 |
|  | chr15 | 78340443 | 78340443 | * | 7.76470976030179e-06 | 0.036427241008796 | -9.56212837679667 |
|  | chr15 | 78832125 | 78832125 | * | 9.70833249582309e-06 | 0.0381570098837087 | 5.24475524475524 |
|  | chr15 | 81002184 | 81002184 | * | 1.10631804274992e-05 | 0.0401362202903679 | -5.46939131953541 |
|  | chr15 | 81865815 | 81865815 | * | 8.82600055057727e-06 | 0.0374335718834972 | -5.82895198037154 |
|  | chr15 | 84715978 | 84715978 | * | 1.2651286632394e-05 | 0.0430140569446174 | -2.18978102189781 |
|  | chr15 | 89406332 | 89406332 | * | 1.76551103445749e-07 | 0.00916830553644203 | 6.59379720963299 |
|  | chr15 | 90067354 | 90067354 | * | 1.13288070783756e-05 | 0.0403759678028314 | 6.86238532110092 |
|  | chr15 | 93234243 | 93234243 | * | 1.1836783182022e-05 | 0.0413402535516275 | 4.9079754601227 |
|  | chr15 | 94081366 | 94081366 | * | 1.53015725454146e-05 | 0.045130930916365 | -8.264236618667 |
|  | chr15 | 96330636 | 96330636 | * | 1.76735637266945e-05 | 0.0472318774384622 | 2.06766917293233 |
|  | chr15 | 97960648 | 97960648 | * | 1.87463855185146e-05 | 0.0476533901794531 | -2.26244343891403 |
|  | chr15 | 98665242 | 98665242 | * | 5.09628135825141e-07 | 0.0138117180710717 | -4.98533724340176 |
|  | chr15 | 100880180 | 100880180 | * | 4.63859976467095e-06 | 0.0308169634740055 | 9.02228976697062 |
|  | chr16 | 528207 | 528207 | * | 1.77494581832474e-05 | 0.0472518712534723 | -2.43478260869565 |
|  | chr16 | 636313 | 636313 | * | 1.19298238372008e-05 | 0.0413688617546217 | 2.82828282828283 |
|  | chr16 | 648887 | 648887 | * | 6.24093240657017e-06 | 0.0330331766726775 | 4.02476780185759 |
|  | chr16 | 723151 | 723151 | * | 1.27484778221078e-05 | 0.0432239356914237 | 5.01557541515532 |
|  | chr16 | 1195816 | 1195816 | * | 1.07416734958392e-05 | 0.0399184503837769 | -8.47457627118644 |
|  | chr16 | 1281584 | 1281584 | * | 1.55509801392747e-05 | 0.0454391445067938 | 5.16557324031509 |
|  | chr16 | 1524968 | 1524968 | * | 1.73875394171272e-05 | 0.0469912079857756 | 9.12394898070895 |
|  | chr16 | 1688286 | 1688286 | * | 2.25646230254108e-06 | 0.0253491391005217 | 4.59770114942529 |
|  | chr16 | 1819157 | 1819157 | * | 4.54431901946557e-06 | 0.030682714293622 | 3.05010893246187 |
|  | chr16 | 1828109 | 1828109 | * | 7.57872747487572e-06 | 0.0361551573799559 | 16.6962100109732 |
|  | chr16 | 1953552 | 1953552 | * | 9.3284999470534e-06 | 0.0380630407857723 | -6.8529304696032 |
|  | chr16 | 2000088 | 2000088 | * | 1.34593668387959e-05 | 0.0440049293460441 | -7.56229324388187 |
|  | chr16 | 2077125 | 2077125 | * | 1.19084264385289e-05 | 0.0413534866384187 | -2.12071778140294 |
|  | chr16 | 2182642 | 2182642 | * | 7.61469375846665e-06 | 0.0361593579075616 | 7.02168156239655 |
|  | chr16 | 3146855 | 3146855 | * | 5.17780756686582e-06 | 0.0312632216132437 | -2.97397769516728 |
|  | chr16 | 4115809 | 4115809 | * | 1.85475653809299e-05 | 0.0473950954187149 | 2.83975659229209 |
|  | chr16 | 4698545 | 4698545 | * | 1.84684157277426e-05 | 0.0473417154885465 | -2.69151138716356 |
|  | chr16 | 10058116 | 10058116 | * | 5.42637538979461e-06 | 0.0318323686811784 | -10.1684952978056 |
|  | chr16 | 10391769 | 10391769 | * | 1.9097475834545e-05 | 0.0480448739266138 | -3.39457010371315 |
|  | chr16 | 11606727 | 11606727 | * | 1.48990597702656e-05 | 0.0450070132898113 | 8.60943636263067 |
|  | chr16 | 11643857 | 11643857 | * | 2.46606747575887e-06 | 0.0262633459760735 | 3.90625 |
|  | chr16 | 12145008 | 12145008 | * | 6.6351057618615e-06 | 0.0339097303865037 | 4.59363957597173 |
|  | chr16 | 15055447 | 15055447 | * | 7.89624512339932e-06 | 0.0365262319343656 | -5.82417582417582 |
|  | chr16 | 15495180 | 15495180 | * | 6.81960388495887e-06 | 0.0344685260247189 | 4.09836065573771 |
|  | chr16 | 15554887 | 15554887 | * | 2.1446829074803e-06 | 0.0251778165859805 | -5.40617405243291 |
|  | chr16 | 17272456 | 17272456 | * | 1.8068290137869e-05 | 0.0473326884493546 | -2.0618556701031 |
|  | chr16 | 21278112 | 21278112 | * | 1.11396472175464e-05 | 0.0401362202903679 | -6.80981715067957 |
|  | chr16 | 23182734 | 23182734 | * | 2.9840490771326e-06 | 0.0279786966436556 | 2.74725274725275 |
|  | chr16 | 28584705 | 28584705 | * | 4.20742508563738e-06 | 0.0304426130789399 | 3.32594235033259 |
|  | chr16 | 29143562 | 29143562 | * | 5.45571555461598e-07 | 0.0143256643071847 | -8.70111399911725 |
|  | chr16 | 29233991 | 29233991 | * | 1.10471161754261e-05 | 0.0401362202903679 | -4.70071912013537 |
|  | chr16 | 30417994 | 30417994 | * | 1.09938954483123e-05 | 0.0401362202903679 | -2.42914979757085 |
|  | chr16 | 30679832 | 30679832 | * | 5.41250667129849e-06 | 0.0318323686811784 | 3.27510917030568 |
|  | chr16 | 31044697 | 31044697 | * | 8.08756021859503e-06 | 0.0370596028661786 | -5.63421375921376 |
|  | chr16 | 46643022 | 46643022 | * | 8.53370667554669e-06 | 0.0372521086012845 | 3.77936936018243 |
|  | chr16 | 48644650 | 48644650 | * | 4.88031515740045e-06 | 0.0310406905817438 | 3.12513635655292 |
|  | chr16 | 49011044 | 49011044 | * | 1.01314673896304e-05 | 0.038905815221874 | 8.65719340295612 |
|  | chr16 | 55327763 | 55327763 | * | 5.21315154057315e-06 | 0.0312632216132437 | 15.4069582378406 |
|  | chr16 | 56491119 | 56491119 | * | 8.12297050805583e-06 | 0.0370703970092214 | -2.734375 |
|  | chr16 | 57753491 | 57753491 | * | 8.62757015048641e-06 | 0.0372521086012845 | 8.61391694725028 |
|  | chr16 | 65121423 | 65121423 | * | 1.33836891994042e-06 | 0.0207718892560112 | -5.99316192698312 |
|  | chr16 | 67155075 | 67155075 | * | 1.92341394100264e-05 | 0.0481402862826006 | 16.502024291498 |
|  | chr16 | 69350197 | 69350197 | * | 1.5957735945888e-05 | 0.0455833711863926 | -3.96796091686312 |
|  | chr16 | 69782054 | 69782054 | * | 4.05383377853349e-06 | 0.0300127101341861 | 6.12787048923121 |
|  | chr16 | 71565258 | 71565258 | * | 1.34657461902562e-05 | 0.0440049293460441 | 2.80373831775701 |
|  | chr16 | 71812742 | 71812742 | * | 8.62417616260912e-06 | 0.0372521086012845 | -4.73545340924872 |
|  | chr16 | 72934109 | 72934109 | * | 8.86017329555222e-06 | 0.0374335718834972 | -7.67656765676568 |
|  | chr16 | 73555375 | 73555375 | * | 3.68044888302104e-06 | 0.0293699201736525 | 5.28886684849987 |
|  | chr16 | 75297543 | 75297543 | * | 1.34628875857225e-06 | 0.0207718892560112 | -8.56848037464777 |
|  | chr16 | 83500831 | 83500831 | * | 8.54546316214799e-06 | 0.0372521086012845 | 5.40583386176284 |
|  | chr16 | 84666928 | 84666928 | * | 9.47052910927902e-06 | 0.0380630407857723 | -10.236623841275 |
|  | chr16 | 85605796 | 85605796 | * | 2.7249257593712e-08 | 0.00824603504446799 | -11.2956682595961 |
|  | chr16 | 85753446 | 85753446 | * | 3.54744131636938e-06 | 0.0293113670780731 | 6.13373064498162 |
|  | chr16 | 86682101 | 86682101 | * | 3.84843083843574e-06 | 0.0298414787713016 | 4.57487845392286 |
|  | chr16 | 86987746 | 86987746 | * | 9.35698741298534e-06 | 0.0380630407857723 | 4.37956204379562 |
|  | chr16 | 87058046 | 87058046 | * | 6.04920590048145e-06 | 0.0329166188805524 | -2.81425891181989 |
|  | chr16 | 87434146 | 87434146 | * | 2.26989375803759e-07 | 0.0097869679172815 | -6.94980694980695 |
|  | chr16 | 88184579 | 88184579 | * | 2.19789392425501e-06 | 0.0251778165859805 | -5.86714421624042 |
|  | chr16 | 88433486 | 88433486 | * | 8.51463962912233e-07 | 0.0177408848071462 | -6.3605640765569 |
|  | chr16 | 88473624 | 88473624 | * | 1.11741870431092e-05 | 0.0401362202903679 | 5.48210638238339 |
|  | chr16 | 88475446 | 88475446 | * | 5.07576494618458e-06 | 0.0310952225336125 | 14.3390336590662 |
|  | chr16 | 88656316 | 88656316 | * | 1.88208250572911e-06 | 0.0236445123895326 | -5.50847457627118 |
|  | chr16 | 88733365 | 88733365 | * | 5.25271079496041e-06 | 0.0313079800175066 | -9.14368965143132 |
|  | chr16 | 88740150 | 88740150 | * | 1.76523088214318e-05 | 0.0472318774384622 | -3.88059701492537 |
|  | chr16 | 89255519 | 89255519 | * | 8.62597762046775e-06 | 0.0372521086012845 | -3.62148906191342 |
|  | chr16 | 89636622 | 89636622 | * | 4.61466699448904e-06 | 0.0308169634740055 | -5.01359106010268 |
|  | chr16 | 89935620 | 89935620 | * | 3.05760557790092e-06 | 0.0282721671447522 | -3.94366197183098 |
|  | chr17 | 979310 | 979310 | * | 8.58366880181788e-06 | 0.0372521086012845 | -2.55972696245734 |
|  | chr17 | 1647242 | 1647242 | * | 6.62848570784339e-06 | 0.0339097303865037 | 3.30330330330331 |
|  | chr17 | 2057234 | 2057234 | * | 8.1811554892772e-06 | 0.0370703970092214 | -5.95023359512419 |
|  | chr17 | 2562012 | 2562012 | * | 7.80472997318835e-06 | 0.0364370944808255 | -5.1063829787234 |
|  | chr17 | 2755364 | 2755364 | * | 6.9590512196401e-06 | 0.034843158469973 | -2.5974025974026 |
|  | chr17 | 3550775 | 3550775 | * | 1.10309681987029e-05 | 0.0401362202903679 | -3.67647058823529 |
|  | chr17 | 3910119 | 3910119 | * | 2.87371439381913e-07 | 0.0101861931984575 | -4.25 |
|  | chr17 | 3956007 | 3956007 | * | 1.3596006575634e-05 | 0.0440049293460441 | -6.7734289745675 |
|  | chr17 | 4694698 | 4694698 | * | 8.17450387245036e-06 | 0.0370703970092214 | 3.44350350110718 |
|  | chr17 | 5868421 | 5868421 | * | 1.61064055817842e-05 | 0.0455833711863926 | 7.26487361181798 |
|  | chr17 | 6473870 | 6473870 | * | 1.59089631910141e-07 | 0.00890507499086869 | -6.98370993168681 |
|  | chr17 | 6713353 | 6713353 | * | 1.27179494257887e-07 | 0.00890507499086869 | 8.34667474362858 |
|  | chr17 | 7330535 | 7330535 | * | 1.37231372157035e-05 | 0.044134586923446 | -4.03305053952004 |
|  | chr17 | 7650554 | 7650554 | * | 2.09466332661193e-05 | 0.0496724956583141 | -14.0022264169208 |
|  | chr17 | 8942827 | 8942827 | * | 2.28838005730228e-07 | 0.0097869679172815 | -6.95047692045648 |
|  | chr17 | 14219812 | 14219812 | * | 1.709534338928e-05 | 0.0468130072138101 | 3.58126721763086 |
|  | chr17 | 16920704 | 16920704 | * | 7.67709662240533e-06 | 0.0361593579075616 | 5.19502276915579 |
|  | chr17 | 17261440 | 17261440 | * | 4.09265217216265e-07 | 0.0124712422469488 | -5.55555555555556 |
|  | chr17 | 18971379 | 18971379 | * | 1.6198221806695e-05 | 0.0455833711863926 | -5.88235294117647 |
|  | chr17 | 19384491 | 19384491 | * | 1.93459722575754e-06 | 0.0238188196676852 | -10.4084625052367 |
|  | chr17 | 21172624 | 21172624 | * | 1.25656884211065e-06 | 0.0200211823860012 | -4.2713567839196 |
|  | chr17 | 27893449 | 27893449 | * | 4.81638574533175e-06 | 0.0310406905817438 | -2.65957446808511 |
|  | chr17 | 29640319 | 29640319 | * | 3.48279590062638e-06 | 0.0293113670780731 | -3.4965034965035 |
|  | chr17 | 30083612 | 30083612 | * | 1.95439701320841e-05 | 0.0484431948255683 | -3.26633165829145 |
|  | chr17 | 32415088 | 32415088 | * | 2.30827846354502e-06 | 0.0255776349518986 | -6.0077519379845 |
|  | chr17 | 36943467 | 36943467 | * | 5.56567036933874e-06 | 0.0321514005789273 | 3.8860103626943 |
|  | chr17 | 38510034 | 38510034 | * | 4.88545383202894e-06 | 0.0310406905817438 | -2.97029702970297 |
|  | chr17 | 38719720 | 38719720 | * | 8.13658826835299e-06 | 0.0370703970092214 | -9.23084611924634 |
|  | chr17 | 41625752 | 41625752 | * | 1.39130893768451e-07 | 0.00890507499086869 | -19.2470837751856 |
|  | chr17 | 41625762 | 41625762 | * | 4.56509506351869e-07 | 0.0132484602073032 | -18.1743970315399 |
|  | chr17 | 42050158 | 42050158 | * | 1.63067516105198e-06 | 0.0223327559295485 | -12.960102960103 |
|  | chr17 | 43818546 | 43818546 | * | 5.73556467190143e-07 | 0.0144075326810445 | 3.95010022909507 |
|  | chr17 | 44297946 | 44297946 | * | 1.43354523740486e-05 | 0.0445180990564497 | -12.9502035703101 |
|  | chr17 | 44382946 | 44382946 | * | 4.36011871899615e-06 | 0.030682714293622 | -3.15765884116657 |
|  | chr17 | 45013881 | 45013881 | * | 3.52063631453378e-06 | 0.0293113670780731 | -5.53057397221118 |
|  | chr17 | 47738852 | 47738852 | * | 5.96214033527946e-06 | 0.0328824397860396 | -12.7690038181187 |
|  | chr17 | 58486139 | 58486139 | * | 1.59198973821673e-05 | 0.0455833711863926 | 5.03875968992248 |
|  | chr17 | 58531991 | 58531991 | * | 7.83213491114159e-06 | 0.0364370944808255 | 2.66666666666667 |
|  | chr17 | 60686059 | 60686059 | * | 1.3914391299053e-05 | 0.0444564254912033 | 4.4280442804428 |
|  | chr17 | 64511366 | 64511366 | * | 5.36404914399734e-06 | 0.0316619429790608 | -2.73972602739726 |
|  | chr17 | 64780197 | 64780197 | * | 1.72123207562002e-05 | 0.0468415980941163 | 8.58070500927644 |
|  | chr17 | 66660553 | 66660553 | * | 1.28398259149301e-05 | 0.0433070426297305 | 7.82378158844765 |
|  | chr17 | 72121241 | 72121241 | * | 1.92173048282734e-05 | 0.0481402862826006 | -8.18021384823004 |
|  | chr17 | 74431133 | 74431133 | * | 8.82083871407401e-06 | 0.0374335718834972 | -3.51648351648352 |
|  | chr17 | 74466822 | 74466822 | * | 1.86765111144588e-05 | 0.0475253263232022 | -4.50101552549141 |
|  | chr17 | 74791607 | 74791607 | * | 6.11024188621341e-06 | 0.0329578049447931 | -3.15126050420168 |
|  | chr17 | 75616940 | 75616940 | * | 4.04822754044944e-06 | 0.0300127101341861 | 6.0377358490566 |
|  | chr17 | 76298422 | 76298422 | * | 1.7204809710542e-05 | 0.0468415980941163 | 3.64295544645975 |
|  | chr17 | 77460796 | 77460796 | * | 3.0369097467117e-06 | 0.0282569615120547 | -4.86111111111111 |
|  | chr17 | 78841126 | 78841126 | * | 1.8931218575154e-05 | 0.0479130796637049 | 2.62008733624454 |
|  | chr17 | 79111796 | 79111796 | * | 8.51736430148738e-06 | 0.0372521086012845 | 5.27625733777566 |
|  | chr17 | 79183777 | 79183777 | * | 1.54603198471501e-06 | 0.0220402862208335 | 6.01165360659032 |
|  | chr17 | 79841683 | 79841683 | * | 1.48854689190666e-05 | 0.0450070132898113 | -3.11850311850312 |
|  | chr17 | 80005379 | 80005379 | * | 9.38638139031487e-06 | 0.0380630407857723 | -9.7646958885491 |
|  | chr17 | 80083166 | 80083166 | * | 1.99992891384134e-05 | 0.0487725967640558 | 11.1368461686933 |
|  | chr17 | 80109975 | 80109975 | * | 1.50032939939982e-06 | 0.0219905340672504 | 3.43511450381679 |
|  | chr17 | 80116467 | 80116467 | * | 8.6566128590113e-06 | 0.0372521086012845 | 6.70103092783505 |
|  | chr17 | 81231840 | 81231840 | * | 1.18568721492146e-06 | 0.0195973483183763 | 4.12147505422994 |
|  | chr17 | 81354387 | 81354387 | * | 9.68652178253284e-06 | 0.0381570098837087 | -8.28788520597268 |
|  | chr17 | 81873400 | 81873400 | * | 2.92492734744956e-07 | 0.0101861931984575 | -13.6752136752137 |
|  | chr17 | 81873410 | 81873410 | * | 4.52677390094083e-07 | 0.0132484602073032 | -13.4821428571429 |
|  | chr17 | 81873488 | 81873488 | * | 1.89134217742147e-06 | 0.0236445123895326 | -13.315549519837 |
|  | chr17 | 81883700 | 81883700 | * | 1.72056966558778e-05 | 0.0468415980941163 | 3.62735900119078 |
|  | chr17 | 81911993 | 81911993 | * | 3.22096653479158e-06 | 0.0287221794777653 | 14.9084168983579 |
|  | chr17 | 82436997 | 82436997 | * | 1.11027676493504e-06 | 0.019060652905471 | 3.89016018306636 |
|  | chr17 | 82779846 | 82779846 | * | 1.26953844995973e-07 | 0.00890507499086869 | 7.23443223443223 |
|  | chr18 | 2693907 | 2693907 | * | 7.88145542632507e-06 | 0.0365262319343656 | 5.8085194375517 |
|  | chr18 | 5629057 | 5629057 | * | 2.24639275529577e-06 | 0.0253491391005217 | -3.2258064516129 |
|  | chr18 | 5894706 | 5894706 | * | 1.12158899465854e-05 | 0.0401362202903679 | -9.60711050724638 |
|  | chr18 | 7116993 | 7116993 | * | 1.90251831325306e-05 | 0.0479835226325341 | 4.03560467014335 |
|  | chr18 | 8478290 | 8478290 | * | 1.96718141832611e-05 | 0.0484889732288157 | 3.92825250721253 |
|  | chr18 | 13137154 | 13137154 | * | 1.0545669638167e-05 | 0.0398574319718915 | -3.43007915567282 |
|  | chr18 | 13137226 | 13137226 | * | 9.65255779404633e-06 | 0.0381570098837087 | -5.62323396770149 |
|  | chr18 | 13640788 | 13640788 | * | 6.54852330015663e-06 | 0.0338141303613569 | 4.50639166398109 |
|  | chr18 | 21047522 | 21047522 | * | 1.20373666426184e-06 | 0.0195973483183763 | 2.8052805280528 |
|  | chr18 | 29803709 | 29803709 | * | 9.40343571427071e-07 | 0.018338821057925 | -6.89103502097356 |
|  | chr18 | 31034061 | 31034061 | * | 1.56549227533641e-05 | 0.0454698725410608 | -7.16331142507202 |
|  | chr18 | 48357153 | 48357153 | * | 1.4133563761186e-05 | 0.0445180990564497 | -8.28115300531699 |
|  | chr18 | 54909702 | 54909702 | * | 5.80700556009105e-06 | 0.0326933340145263 | -2.55905511811024 |
|  | chr18 | 59347099 | 59347099 | * | 3.34991922100796e-06 | 0.0290437810662865 | 2.96610169491526 |
|  | chr18 | 59409666 | 59409666 | * | 4.28806110926169e-06 | 0.0306440995731637 | 5.93681917211329 |
|  | chr18 | 75238092 | 75238092 | * | 1.08184066901372e-05 | 0.0400638933557008 | 2.93453724604966 |
|  | chr18 | 76807193 | 76807193 | * | 1.45882812525116e-05 | 0.0448462162021566 | -3.6782957762867 |
|  | chr18 | 79807606 | 79807606 | * | 1.18867560018193e-05 | 0.0413534866384187 | -6.41162946779191 |
|  | chr19 | 503310 | 503310 | * | 1.48243423594117e-05 | 0.0449484016985218 | -7.01166810717373 |
|  | chr19 | 869252 | 869252 | * | 1.95909299290831e-05 | 0.0484431948255683 | 3.88330805073649 |
|  | chr19 | 1276284 | 1276284 | * | 2.68068970782506e-06 | 0.0274112604430449 | 4.01002506265664 |
|  | chr19 | 2131707 | 2131707 | * | 1.59397309036056e-06 | 0.0223141858609182 | -7.02166863207547 |
|  | chr19 | 2513495 | 2513495 | * | 1.35036652253201e-05 | 0.0440049293460441 | 3.76068376068376 |
|  | chr19 | 2776058 | 2776058 | * | 9.44529655789387e-06 | 0.0380630407857723 | -2.97872340425532 |
|  | chr19 | 2776076 | 2776076 | * | 1.02450839575544e-05 | 0.0392076628888773 | 2.17729393468118 |
|  | chr19 | 3712729 | 3712729 | * | 1.37723165266587e-05 | 0.0441833825925084 | -4.70588235294118 |
|  | chr19 | 3770676 | 3770676 | * | 1.5054744772536e-05 | 0.0451189213522599 | -4.27350427350427 |
|  | chr19 | 4544447 | 4544447 | * | 3.37719954240298e-06 | 0.0290437810662865 | -9.89071038251367 |
|  | chr19 | 5409826 | 5409826 | * | 1.11909653923411e-05 | 0.0401362202903679 | 7.30447140029438 |
|  | chr19 | 5734331 | 5734331 | * | 7.09319338943809e-06 | 0.0349354795451427 | -4.33222918143289 |
|  | chr19 | 7204852 | 7204852 | * | 1.03697755693545e-05 | 0.0394372109625776 | 5.9500357945148 |
|  | chr19 | 7557027 | 7557027 | * | 1.51568352244114e-05 | 0.0451189213522599 | -2.8322440087146 |
|  | chr19 | 7612451 | 7612451 | * | 9.24868480766366e-06 | 0.0380630407857723 | -3.85339941403575 |
|  | chr19 | 12063404 | 12063404 | * | 1.57024277726076e-05 | 0.0455161662073281 | 3.84615384615384 |
|  | chr19 | 12147203 | 12147203 | * | 1.41560900543695e-05 | 0.0445180990564497 | -3.97444874744558 |
|  | chr19 | 12550421 | 12550421 | * | 1.63479351815869e-05 | 0.0458077035894703 | -3.33818976507011 |
|  | chr19 | 13173929 | 13173929 | * | 6.13313161448906e-06 | 0.0330049400738021 | -3.01934224535668 |
|  | chr19 | 13750388 | 13750388 | * | 1.38914969687984e-06 | 0.0209048346176606 | -6.06060606060606 |
|  | chr19 | 15238894 | 15238894 | * | 2.00531780361329e-05 | 0.0487725967640558 | -5.0407373350726 |
|  | chr19 | 15346877 | 15346877 | * | 1.40252435554679e-05 | 0.0445180990564497 | -2.90556900726392 |
|  | chr19 | 15449266 | 15449266 | * | 1.07223585593818e-05 | 0.0399184503837769 | -3.89972144846797 |
|  | chr19 | 16129661 | 16129661 | * | 1.52330081110754e-05 | 0.0451212065358854 | 6.7256162915327 |
|  | chr19 | 17332059 | 17332059 | * | 2.92078622217121e-07 | 0.0101861931984575 | -5.01474926253688 |
|  | chr19 | 17517406 | 17517406 | * | 1.3430074134477e-05 | 0.0440049293460441 | -2.68041237113402 |
|  | chr19 | 18142571 | 18142571 | * | 6.02566438344252e-07 | 0.0144075326810445 | -4.23447429181286 |
|  | chr19 | 18157995 | 18157995 | * | 5.84818832436114e-07 | 0.0144075326810445 | 5.36086587859743 |
|  | chr19 | 19821433 | 19821433 | * | 8.48667550263402e-06 | 0.0372521086012845 | -5.24193548387097 |
|  | chr19 | 20080509 | 20080509 | * | 1.77691261373533e-05 | 0.0472518712534723 | -3.72734330171802 |
|  | chr19 | 21310185 | 21310185 | * | 2.54087982434205e-06 | 0.0266987547411388 | -2.78372591006424 |
|  | chr19 | 23768061 | 23768061 | * | 9.13444255732159e-07 | 0.0181038858912785 | -4.72636815920398 |
|  | chr19 | 31980460 | 31980460 | * | 8.85844694576312e-06 | 0.0374335718834972 | 4.03738207023208 |
|  | chr19 | 32883098 | 32883098 | * | 6.09193650607898e-06 | 0.0329578049447931 | 4.14201183431953 |
|  | chr19 | 32889776 | 32889776 | * | 1.47029137660833e-05 | 0.0449484016985218 | -2.74296901576634 |
|  | chr19 | 33080004 | 33080004 | * | 9.63125122012023e-06 | 0.0381570098837087 | -4.71380471380471 |
|  | chr19 | 33903218 | 33903218 | * | 1.62459699221235e-05 | 0.0456268813925046 | 12.6520376175549 |
|  | chr19 | 34384570 | 34384570 | * | 8.74180331396284e-07 | 0.0179080762742176 | -6.47773279352226 |
|  | chr19 | 34980047 | 34980047 | * | 1.19589065869655e-05 | 0.0414108057065142 | 4.36485874975758 |
|  | chr19 | 35820536 | 35820536 | * | 9.10651571280731e-06 | 0.0380209070549651 | 12.4280677117519 |
|  | chr19 | 35864054 | 35864054 | * | 1.01228578721321e-05 | 0.038905815221874 | -5.8023993467747 |
|  | chr19 | 35977168 | 35977168 | * | 1.29651953533199e-05 | 0.0435094192554022 | 8.64762840235547 |
|  | chr19 | 37551642 | 37551642 | * | 8.62717646003238e-06 | 0.0372521086012845 | -7.58107374076956 |
|  | chr19 | 37769154 | 37769154 | * | 2.14441106412612e-07 | 0.00968075313468055 | 6.34328358208955 |
|  | chr19 | 38129392 | 38129392 | * | 8.76438017052516e-06 | 0.0374335718834972 | -3.63636363636364 |
|  | chr19 | 38418320 | 38418320 | * | 7.09376899773877e-06 | 0.0349354795451427 | -3.91644908616188 |
|  | chr19 | 38869437 | 38869437 | * | 2.18161623627985e-06 | 0.0251778165859805 | -7.03656238220882 |
|  | chr19 | 39795543 | 39795543 | * | 1.07137855037403e-05 | 0.0399184503837769 | -6.93858759991024 |
|  | chr19 | 39833531 | 39833531 | * | 1.25511071716094e-05 | 0.0428806450960843 | 7.00350813248895 |
|  | chr19 | 41652782 | 41652782 | * | 3.22053878299194e-06 | 0.0287221794777653 | 4.92467772788522 |
|  | chr19 | 41777473 | 41777473 | * | 2.71698058032977e-07 | 0.0101861931984575 | -7.55689900426743 |
|  | chr19 | 42797859 | 42797859 | * | 3.99668563165043e-08 | 0.00863652823139316 | -4.0241448692153 |
|  | chr19 | 44668867 | 44668867 | * | 1.74265200610954e-05 | 0.0469912079857756 | -2.41684566816377 |
|  | chr19 | 45081326 | 45081326 | * | 5.24928872797147e-06 | 0.0313079800175066 | 3.77833753148614 |
|  | chr19 | 45104182 | 45104182 | * | 4.17860993303431e-06 | 0.0304426130789399 | 6.63788278564004 |
|  | chr19 | 46281358 | 46281358 | * | 7.85997566308641e-06 | 0.0364969660798288 | 13.485563289916 |
|  | chr19 | 46485969 | 46485969 | * | 7.55390997047322e-06 | 0.0361074230502748 | -5.3156146179402 |
|  | chr19 | 46809485 | 46809485 | * | 6.43203260950201e-06 | 0.033554488025128 | 3.54609929078015 |
|  | chr19 | 47288195 | 47288195 | * | 3.21332482962088e-06 | 0.0287221794777653 | 7.19164740409838 |
|  | chr19 | 48500194 | 48500194 | * | 1.48504161927735e-08 | 0.00724041545745036 | -6.2944056981748 |
|  | chr19 | 48782372 | 48782372 | * | 1.63403933388894e-07 | 0.00890507499086869 | 13.0068866048008 |
|  | chr19 | 48851383 | 48851383 | * | 5.93245730121458e-06 | 0.0328824397860396 | -6.62867718683031 |
|  | chr19 | 49056582 | 49056582 | * | 1.82833520496533e-06 | 0.0236445123895326 | -9.59547767753875 |
|  | chr19 | 50556192 | 50556192 | * | 1.03006801850446e-05 | 0.0393467973402204 | -3.59897172236504 |
|  | chr19 | 53753214 | 53753214 | * | 1.76360456818626e-06 | 0.0229908207028997 | 3.72222222222222 |
|  | chr19 | 54200465 | 54200465 | * | 6.25087124732261e-06 | 0.0330331766726775 | -11.5153724632398 |
|  | chr19 | 55335787 | 55335787 | * | 2.55289614565807e-08 | 0.00824603504446799 | -6.28547045018135 |
|  | chr19 | 56507727 | 56507727 | * | 1.32201138417753e-05 | 0.0439669243675726 | 5.07913436692506 |
|  | chr2 | 3060306 | 3060306 | * | 6.3357868445665e-06 | 0.0332872136325884 | 5.34015080340151 |
|  | chr2 | 3518790 | 3518790 | * | 9.27174510750642e-06 | 0.0380630407857723 | -2 |
|  | chr2 | 7179324 | 7179324 | * | 4.25133743610864e-08 | 0.00863652823139316 | 4.98494727647095 |
|  | chr2 | 9387032 | 9387032 | * | 1.03298614468977e-05 | 0.0393467973402204 | 6.87749212847333 |
|  | chr2 | 10567437 | 10567437 | * | 1.30428374897604e-05 | 0.0436153559142155 | 3.03030303030303 |
|  | chr2 | 14633714 | 14633714 | * | 1.50645922778609e-06 | 0.0219905340672504 | 11.3234319210058 |
|  | chr2 | 15694273 | 15694273 | * | 1.0464793302663e-05 | 0.0397365806027297 | -9.79475281099411 |
|  | chr2 | 20506836 | 20506836 | * | 8.3932675976247e-06 | 0.0372521086012845 | -5.46625626994984 |
|  | chr2 | 23547143 | 23547143 | * | 1.62441207209869e-06 | 0.0223327559295485 | -10.7977062223682 |
|  | chr2 | 26185120 | 26185120 | * | 8.22003887258543e-06 | 0.0371086348201649 | 3.26340326340326 |
|  | chr2 | 26881570 | 26881570 | * | 9.26993540080694e-06 | 0.0380630407857723 | -4.84429065743944 |
|  | chr2 | 31169035 | 31169035 | * | 2.84991049921304e-08 | 0.00824603504446799 | 4.94505494505495 |
|  | chr2 | 37196119 | 37196119 | * | 2.05850218177872e-05 | 0.0491534773469436 | 3.11175641268439 |
|  | chr2 | 38510819 | 38510819 | * | 1.97731861157386e-05 | 0.0486224437851043 | 5.23609281588814 |
|  | chr2 | 41578272 | 41578272 | * | 1.42730483707372e-05 | 0.0445180990564497 | 7.73681005370094 |
|  | chr2 | 42076589 | 42076589 | * | 1.85536375776509e-06 | 0.0236445123895326 | 5.025931279074 |
|  | chr2 | 42594506 | 42594506 | * | 4.22088735608093e-06 | 0.0304426130789399 | 2.65486725663717 |
|  | chr2 | 54330758 | 54330758 | * | 1.58709109262172e-05 | 0.0455833711863926 | -2.11538461538462 |
|  | chr2 | 55280921 | 55280921 | * | 1.38516512850369e-05 | 0.0443722814361888 | 4.79680908825126 |
|  | chr2 | 65171356 | 65171356 | * | 1.511623207943e-05 | 0.0451189213522599 | 4.53053499802272 |
|  | chr2 | 69632815 | 69632815 | * | 1.10801810557548e-05 | 0.0401362202903679 | 2.80561122244489 |
|  | chr2 | 70586102 | 70586102 | * | 1.05185591883869e-06 | 0.0187043339204075 | -3.95073344914109 |
|  | chr2 | 70840447 | 70840447 | * | 1.10734485377707e-06 | 0.019060652905471 | 4.32964061043313 |
|  | chr2 | 71270340 | 71270340 | * | 1.23759215274589e-06 | 0.0198485517980604 | 3.19361277445109 |
|  | chr2 | 73177414 | 73177414 | * | 1.67004654543697e-05 | 0.0465779040949594 | -3.75586768238483 |
|  | chr2 | 73269133 | 73269133 | * | 8.21972050531201e-06 | 0.0371086348201649 | 16.322235464158 |
|  | chr2 | 78074918 | 78074918 | * | 1.75915541418018e-06 | 0.0229908207028997 | -12.272304214865 |
|  | chr2 | 85592524 | 85592524 | * | 6.28209160045668e-07 | 0.0146149559616363 | 2.62345679012346 |
|  | chr2 | 87825825 | 87825825 | * | 7.36002479871658e-06 | 0.0355994761386208 | 9.97453676499392 |
|  | chr2 | 88627498 | 88627498 | * | 5.36192788411314e-06 | 0.0316619429790608 | -5.55555555555556 |
|  | chr2 | 96790253 | 96790253 | * | 3.40722356523081e-06 | 0.0291440992185807 | -3.18725099601593 |
|  | chr2 | 99077783 | 99077783 | * | 1.5213728443133e-05 | 0.0451189213522599 | -9.69531928328992 |
|  | chr2 | 105745225 | 105745225 | * | 3.55177527653744e-06 | 0.0293113670780731 | -10.4872342589767 |
|  | chr2 | 127068639 | 127068639 | * | 3.74615498547587e-06 | 0.0294590619122689 | 4.87588652482269 |
|  | chr2 | 127873992 | 127873992 | * | 1.32786576617068e-05 | 0.0439956890457087 | -4.5590627432861 |
|  | chr2 | 128516089 | 128516089 | * | 2.03333831023186e-05 | 0.0488880499261265 | -8.12004164123918 |
|  | chr2 | 139255961 | 139255961 | * | 8.77368630862543e-06 | 0.0374335718834972 | -6.93014257860102 |
|  | chr2 | 144053926 | 144053926 | * | 1.9293338362478e-06 | 0.0238188196676852 | -2.38532110091743 |
|  | chr2 | 159213862 | 159213862 | * | 3.31867698445475e-06 | 0.0290437810662865 | -3.59213523792763 |
|  | chr2 | 172090716 | 172090716 | * | 1.56678049696368e-05 | 0.0454698725410608 | 10.3960259035811 |
|  | chr2 | 177225839 | 177225839 | * | 1.6203219549711e-05 | 0.0455833711863926 | -4.88214683866858 |
|  | chr2 | 180555705 | 180555705 | * | 1.59523265153219e-05 | 0.0455833711863926 | 6.14825395081398 |
|  | chr2 | 186645694 | 186645694 | * | 1.04928679745252e-06 | 0.0187043339204075 | 3.42741935483871 |
|  | chr2 | 188292739 | 188292739 | * | 5.2119818586128e-06 | 0.0312632216132437 | 3.60163008706779 |
|  | chr2 | 199486626 | 199486626 | * | 1.82334475486978e-05 | 0.0473326884493546 | -6.22529644268774 |
|  | chr2 | 199912789 | 199912789 | * | 8.27793038908127e-06 | 0.0372521086012845 | 5.03909952606635 |
|  | chr2 | 200502801 | 200502801 | * | 1.79980114179843e-05 | 0.0472793408087894 | 4.02664692820133 |
|  | chr2 | 203784447 | 203784447 | * | 1.60167413335125e-05 | 0.0455833711863926 | 6.31642512077295 |
|  | chr2 | 206270815 | 206270815 | * | 7.82661418497281e-06 | 0.0364370944808255 | -14.2554222706895 |
|  | chr2 | 207882410 | 207882410 | * | 1.14390039548005e-06 | 0.0193474967675106 | -5.51531654871801 |
|  | chr2 | 210272556 | 210272556 | * | 3.96430539125759e-06 | 0.0300127101341861 | 5.86786438713417 |
|  | chr2 | 218031936 | 218031936 | * | 1.26001476900141e-05 | 0.0429600185119693 | -9.23278792818942 |
|  | chr2 | 218446908 | 218446908 | * | 5.2195525262678e-06 | 0.0312632216132437 | -4.61269365317342 |
|  | chr2 | 223863350 | 223863350 | * | 8.63045782699174e-06 | 0.0372521086012845 | -4.61968622171595 |
|  | chr2 | 224858408 | 224858408 | * | 6.34297372911658e-07 | 0.0146149559616363 | -5.53814601087884 |
|  | chr2 | 230725168 | 230725168 | * | 3.3435837800574e-06 | 0.0290437810662865 | 3.09145556032632 |
|  | chr2 | 230929092 | 230929092 | * | 6.56091630052311e-06 | 0.0338141303613569 | -4.48386333149214 |
|  | chr2 | 231379155 | 231379155 | * | 1.88858391061176e-05 | 0.0479079694357851 | 2.74398729514236 |
|  | chr2 | 232366383 | 232366383 | * | 1.06968945330462e-06 | 0.0187079686720523 | -3.470715835141 |
|  | chr2 | 232486584 | 232486584 | * | 4.48480502514252e-06 | 0.030682714293622 | 6.39569892473118 |
|  | chr2 | 234793613 | 234793613 | * | 1.22976761034148e-05 | 0.0424031873362585 | 10.8605776279138 |
|  | chr2 | 234951370 | 234951370 | * | 4.02881758287213e-06 | 0.0300127101341861 | -2.40963855421687 |
|  | chr2 | 237037291 | 237037291 | * | 7.78518857124334e-06 | 0.036427241008796 | -4.29387054786415 |
|  | chr2 | 239066839 | 239066839 | * | 2.86032576951199e-06 | 0.0274211735986337 | 4.69179082676016 |
|  | chr2 | 240568661 | 240568661 | * | 1.83248334080095e-05 | 0.0473326884493546 | -2.21130221130221 |
|  | chr2 | 240791310 | 240791310 | * | 3.69377384092875e-06 | 0.0293699201736525 | 11.9944237746614 |
|  | chr2 | 241730565 | 241730565 | * | 9.4499561434546e-06 | 0.0380630407857723 | -8.94260082826142 |
|  | chr20 | 5784952 | 5784952 | * | 1.58433174920647e-05 | 0.0455833711863926 | -3.06259622244752 |
|  | chr20 | 9067864 | 9067864 | * | 3.13573221214555e-06 | 0.0285232520283156 | -10.04394385426 |
|  | chr20 | 9516246 | 9516246 | * | 4.84323327572387e-06 | 0.0310406905817438 | -12.7661184542428 |
|  | chr20 | 16702950 | 16702950 | * | 1.70439676182775e-05 | 0.0468130072138101 | -8.57285328492509 |
|  | chr20 | 18468321 | 18468321 | * | 4.39914355571061e-07 | 0.0130782353360089 | 11.8971733951452 |
|  | chr20 | 21101464 | 21101464 | * | 1.06101381698409e-05 | 0.0399184503837769 | 9.59760932599562 |
|  | chr20 | 22583098 | 22583098 | * | 6.10355175810551e-06 | 0.0329578049447931 | 4.07224958949097 |
|  | chr20 | 24798065 | 24798065 | * | 1.79758343775408e-05 | 0.0472793408087894 | 2.72368267196752 |
|  | chr20 | 34591914 | 34591914 | * | 6.19793518887861e-06 | 0.0330331766726775 | 3.420523138833 |
|  | chr20 | 35539102 | 35539102 | * | 1.17760598350228e-05 | 0.0412644725373489 | 3.25941854944564 |
|  | chr20 | 35946959 | 35946959 | * | 1.47710524407847e-05 | 0.0449484016985218 | 4.09704065780001 |
|  | chr20 | 41137361 | 41137361 | * | 1.96052899622684e-06 | 0.0238967114873993 | -2.78481012658228 |
|  | chr20 | 44318999 | 44318999 | * | 1.47807629308555e-05 | 0.0449484016985218 | -4.2204323866045 |
|  | chr20 | 46012443 | 46012443 | * | 4.46344400867106e-06 | 0.030682714293622 | 19.6653528093361 |
|  | chr20 | 49567696 | 49567696 | * | 7.68343822216954e-06 | 0.0361593579075616 | -15.7831790833318 |
|  | chr20 | 54237048 | 54237048 | * | 6.23912938186434e-06 | 0.0330331766726775 | -9.29090426942456 |
|  | chr20 | 55286538 | 55286538 | * | 1.5373427301389e-06 | 0.0220402862208335 | 7.91471506635442 |
|  | chr20 | 56170101 | 56170101 | * | 7.2114642121125e-06 | 0.0351971330669854 | 5.24352432013436 |
|  | chr20 | 57368107 | 57368107 | * | 5.96383687644184e-06 | 0.0328824397860396 | 4.82094982765224 |
|  | chr20 | 61936912 | 61936912 | * | 4.48998040460529e-06 | 0.030682714293622 | -5.96514343894112 |
|  | chr20 | 62170156 | 62170156 | * | 1.41471713666901e-05 | 0.0445180990564497 | 3.87596899224806 |
|  | chr20 | 62596647 | 62596647 | * | 2.90464449714508e-06 | 0.0275521014456783 | -6.77521008403361 |
|  | chr20 | 63540757 | 63540757 | * | 8.43768122529892e-06 | 0.0372521086012845 | -7.51008434176751 |
|  | chr20 | 63856606 | 63856606 | * | 8.57108654720792e-06 | 0.0372521086012845 | -6.4099055066797 |
|  | chr20 | 63969303 | 63969303 | * | 2.11522711880503e-06 | 0.0251534760787062 | 3.77697841726619 |
|  | chr21 | 6121064 | 6121064 | * | 1.84158949731979e-05 | 0.0473326884493546 | 2.12014134275619 |
|  | chr21 | 28142063 | 28142063 | * | 3.6004231520944e-06 | 0.0293113670780731 | -5.02673796791444 |
|  | chr21 | 32392775 | 32392775 | * | 1.02320790337473e-06 | 0.0186146105157064 | -9.24182850788355 |
|  | chr21 | 33493140 | 33493140 | * | 2.2653719835721e-07 | 0.0097869679172815 | -5.97569953023139 |
|  | chr21 | 36113148 | 36113148 | * | 1.90424403702992e-05 | 0.0479835226325341 | 13.0744323135041 |
|  | chr21 | 42625955 | 42625955 | * | 7.40759852388238e-06 | 0.0357586343460338 | -17.835737337812 |
|  | chr21 | 44797512 | 44797512 | * | 1.8277875454657e-05 | 0.0473326884493546 | 2.84360189573459 |
|  | chr21 | 45405983 | 45405983 | * | 1.11948476559435e-05 | 0.0401362202903679 | -2.88659793814433 |
|  | chr21 | 46151255 | 46151255 | * | 6.03715405751714e-06 | 0.0329166188805524 | -8.35916028302438 |
|  | chr22 | 17809069 | 17809069 | * | 2.54036615907902e-06 | 0.0266987547411388 | -3.35195530726257 |
|  | chr22 | 19788690 | 19788690 | * | 4.9571794659257e-06 | 0.0310952225336125 | 4.82854935520295 |
|  | chr22 | 19873657 | 19873657 | * | 2.21022747292409e-06 | 0.0251778165859805 | 4.17857110464115 |
|  | chr22 | 20177246 | 20177246 | * | 1.12924711542286e-05 | 0.0403053922625148 | 5.1296973727968 |
|  | chr22 | 20426147 | 20426147 | * | 2.0604280292774e-05 | 0.0491534773469436 | 13.3109292811221 |
|  | chr22 | 21632415 | 21632415 | * | 1.35492552348341e-05 | 0.0440049293460441 | -4.17001392459297 |
|  | chr22 | 24647034 | 24647034 | * | 8.05592123800035e-06 | 0.037053924127198 | -3.27102803738317 |
|  | chr22 | 28622925 | 28622925 | * | 1.27990837876711e-05 | 0.0433070426297305 | -4.70457079152732 |
|  | chr22 | 29453520 | 29453520 | * | 1.44261367053428e-05 | 0.0446291578551067 | -7.19997594997595 |
|  | chr22 | 30251343 | 30251343 | * | 4.21138705524499e-06 | 0.0304426130789399 | 6.98767898767898 |
|  | chr22 | 31008222 | 31008222 | * | 6.54233670239934e-06 | 0.0338141303613569 | -4.97105334732098 |
|  | chr22 | 33481824 | 33481824 | * | 8.08312114795054e-06 | 0.0370596028661786 | -4.01389865206506 |
|  | chr22 | 33704581 | 33704581 | * | 3.88220264453483e-06 | 0.0298414787713016 | -7.34924483395244 |
|  | chr22 | 34931015 | 34931015 | * | 2.69257270476581e-06 | 0.0274112604430449 | -9.43232544428535 |
|  | chr22 | 35823068 | 35823068 | * | 6.40993558852204e-07 | 0.0146149559616363 | 4.24403183023873 |
|  | chr22 | 36083788 | 36083788 | * | 3.50474436434424e-06 | 0.0293113670780731 | -3.69689187136684 |
|  | chr22 | 37994588 | 37994588 | * | 4.86572403661414e-06 | 0.0310406905817438 | -9.55973530809258 |
|  | chr22 | 38569741 | 38569741 | * | 1.47452186436006e-05 | 0.0449484016985218 | -5.84543530613734 |
|  | chr22 | 39201059 | 39201059 | * | 4.99140182155982e-06 | 0.0310952225336125 | -4.72195648441142 |
|  | chr22 | 39230415 | 39230415 | * | 1.65764850637164e-06 | 0.0224030206914996 | -9.00817412793461 |
|  | chr22 | 39708624 | 39708624 | * | 1.11504712655464e-05 | 0.0401362202903679 | -12.5632481796865 |
|  | chr22 | 41227274 | 41227274 | * | 9.09305001774538e-07 | 0.0181038858912785 | 4.31916528491123 |
|  | chr22 | 42127941 | 42127941 | * | 1.32899077798431e-07 | 0.00890507499086869 | -9.28919843105703 |
|  | chr22 | 42213523 | 42213523 | * | 1.17646992436554e-07 | 0.00890507499086869 | -3.37711069418386 |
|  | chr22 | 42481842 | 42481842 | * | 9.16198873916144e-06 | 0.0380492865971558 | -11.611030478955 |
|  | chr22 | 43059237 | 43059237 | * | 2.3681127894151e-06 | 0.025878361638228 | -4.66666666666666 |
|  | chr22 | 44081296 | 44081296 | * | 5.44515372582594e-06 | 0.0318323686811784 | 11.2433145386557 |
|  | chr22 | 45586859 | 45586859 | * | 1.53933261359085e-06 | 0.0220402862208335 | 4.09724048353295 |
|  | chr22 | 46317682 | 46317682 | * | 1.8257413052357e-05 | 0.0473326884493546 | 5.82239382239381 |
|  | chr22 | 46662092 | 46662092 | * | 2.07977142224067e-05 | 0.049415490616498 | 2.50399678082416 |
|  | chr22 | 46997799 | 46997799 | * | 9.131186746318e-06 | 0.0380209070549651 | -3.58056265984654 |
|  | chr22 | 49023275 | 49023275 | * | 9.47757664834828e-06 | 0.0380630407857723 | 7.48381225054748 |
|  | chr22 | 49785370 | 49785370 | * | 2.85334891007522e-06 | 0.0274211735986337 | -14.2111160914388 |
|  | chr22 | 49785439 | 49785439 | * | 1.53915999245527e-06 | 0.0220402862208335 | -13.9699073216627 |
|  | chr22 | 49818626 | 49818626 | * | 9.39334407196994e-06 | 0.0380630407857723 | -5.15021459227468 |
|  | chr22 | 50287721 | 50287721 | * | 1.28747594077168e-05 | 0.0433070426297305 | 3.46829671770662 |
|  | chr22 | 50287948 | 50287948 | * | 4.47930863881071e-06 | 0.030682714293622 | -5.20591059426011 |
|  | chr22 | 50452489 | 50452489 | * | 7.33273638501887e-08 | 0.00890507499086869 | -4.01891252955082 |
|  | chr22 | 50617385 | 50617385 | * | 6.00623909910068e-08 | 0.00866245804981213 | -7.33478693908315 |
|  | chr3 | 1102890 | 1102890 | * | 1.01258684064912e-05 | 0.038905815221874 | -5.95486255871481 |
|  | chr3 | 2835152 | 2835152 | * | 1.92954697750822e-05 | 0.0482366656690467 | -4.75845802841864 |
|  | chr3 | 6861507 | 6861507 | * | 2.37787997735773e-06 | 0.025878361638228 | -6.76157609620103 |
|  | chr3 | 7661516 | 7661516 | * | 6.83063118101316e-06 | 0.0344685260247189 | -2.65848670756647 |
|  | chr3 | 9704382 | 9704382 | * | 5.73399705522267e-06 | 0.0326306195840953 | 4.78125647936969 |
|  | chr3 | 9883155 | 9883155 | * | 5.64144565427569e-06 | 0.0324354119574799 | -5.46739012184508 |
|  | chr3 | 10145472 | 10145472 | * | 1.11052960508181e-05 | 0.0401362202903679 | -3.17310855263158 |
|  | chr3 | 13450122 | 13450122 | * | 2.02270323303972e-05 | 0.0487725967640558 | 1.98221841977478 |
|  | chr3 | 14130826 | 14130826 | * | 1.78258033079461e-05 | 0.0472518712534723 | 2.71077284912645 |
|  | chr3 | 14340113 | 14340113 | * | 3.20578060953285e-08 | 0.00824603504446799 | -8.5158371040724 |
|  | chr3 | 14514326 | 14514326 | * | 1.5171751047288e-05 | 0.0451189213522599 | 6.15535321821036 |
|  | chr3 | 15288586 | 15288586 | * | 9.71535736221827e-07 | 0.0185826556422598 | -3.41523951352692 |
|  | chr3 | 27728582 | 27728582 | * | 8.57497213324551e-06 | 0.0372521086012845 | 4.01310401310401 |
|  | chr3 | 29930213 | 29930213 | * | 1.82280734167428e-05 | 0.0473326884493546 | 6.81488833746898 |
|  | chr3 | 33761724 | 33761724 | * | 1.77086942059439e-05 | 0.0472318774384622 | -3.39805825242718 |
|  | chr3 | 35639855 | 35639855 | * | 4.91678205068907e-06 | 0.0310952225336125 | -3.19410319410319 |
|  | chr3 | 39527023 | 39527023 | * | 1.55445805837863e-07 | 0.00890507499086869 | 6.67062590021367 |
|  | chr3 | 45065069 | 45065069 | * | 4.4611612883255e-06 | 0.030682714293622 | -6.89116226200625 |
|  | chr3 | 47075028 | 47075028 | * | 1.30781341599428e-05 | 0.0436734795187585 | 4.9947242866318 |
|  | chr3 | 48964299 | 48964299 | * | 9.42063259007722e-06 | 0.0380630407857723 | 3.50877192982456 |
|  | chr3 | 49852979 | 49852979 | * | 1.71050428878769e-05 | 0.0468130072138101 | -3.28027881727511 |
|  | chr3 | 49921566 | 49921566 | * | 1.73031283675869e-05 | 0.0469201945252421 | -6.13859180035651 |
|  | chr3 | 51497501 | 51497501 | * | 4.97709964653017e-07 | 0.0138117180710717 | -3.1772066535196 |
|  | chr3 | 57775647 | 57775647 | * | 5.83682957901816e-06 | 0.0327855255410093 | -4.38247011952191 |
|  | chr3 | 62852461 | 62852461 | * | 7.50488034221988e-06 | 0.0360079652024686 | 7.05741626794258 |
|  | chr3 | 93971023 | 93971023 | * | 1.85250036085417e-05 | 0.047387114739599 | -9.40577249575552 |
|  | chr3 | 95501548 | 95501548 | * | 4.41289948488997e-06 | 0.030682714293622 | -6.07232441471572 |
|  | chr3 | 98593268 | 98593268 | * | 3.57868610167914e-06 | 0.0293113670780731 | 3.64077669902913 |
|  | chr3 | 113532886 | 113532886 | * | 7.34084843293941e-07 | 0.0159780251558463 | -6.48460648698325 |
|  | chr3 | 119498668 | 119498668 | * | 3.88203809381565e-06 | 0.0298414787713016 | -4.53172205438066 |
|  | chr3 | 124785366 | 124785366 | * | 4.67731589017477e-06 | 0.0308169634740055 | -12.3409333584574 |
|  | chr3 | 124887677 | 124887677 | * | 1.81870671825943e-05 | 0.0473326884493546 | 3.35917312661499 |
|  | chr3 | 124914747 | 124914747 | * | 8.48584169081229e-06 | 0.0372521086012845 | -6.08218933856303 |
|  | chr3 | 127408673 | 127408673 | * | 2.24816261011015e-06 | 0.0253491391005217 | -10.2268628001122 |
|  | chr3 | 129404157 | 129404157 | * | 1.62117789115689e-05 | 0.0455833711863926 | 3.34128878281623 |
|  | chr3 | 129886661 | 129886661 | * | 1.79507922306056e-05 | 0.0472793408087894 | 3.43980343980343 |
|  | chr3 | 147410168 | 147410168 | * | 2.81174852277388e-06 | 0.0274210684298071 | -8.31572163016922 |
|  | chr3 | 147410730 | 147410730 | * | 2.09959416018118e-05 | 0.0497198911538814 | -2.65486725663717 |
|  | chr3 | 147991644 | 147991644 | * | 1.67748191983403e-05 | 0.0465779040949594 | -4.75380972618542 |
|  | chr3 | 148987652 | 148987652 | * | 6.97912394769781e-06 | 0.034843158469973 | 2.58267622867242 |
|  | chr3 | 156099734 | 156099734 | * | 8.59159308579968e-07 | 0.0177495177761754 | -6.06812632590161 |
|  | chr3 | 176264816 | 176264816 | * | 3.69867588377932e-06 | 0.0293699201736525 | -9.18793211350953 |
|  | chr3 | 183101241 | 183101241 | * | 2.06067867629576e-05 | 0.0491534773469436 | -4.37710437710438 |
|  | chr3 | 183725038 | 183725038 | * | 5.95715449756191e-06 | 0.0328824397860396 | -4.27631578947368 |
|  | chr3 | 185618675 | 185618675 | * | 3.73518276944404e-07 | 0.0118254043143647 | -6.91080634179554 |
|  | chr3 | 187038409 | 187038409 | * | 6.84342020572962e-06 | 0.0344685260247189 | 3.23755138692071 |
|  | chr3 | 194037208 | 194037208 | * | 1.13510506539632e-05 | 0.0403771468111895 | -3.71734031527846 |
|  | chr3 | 195752184 | 195752184 | * | 1.00620586450236e-06 | 0.0185826556422598 | -8.00451013796296 |
|  | chr3 | 196000518 | 196000518 | * | 2.37463670562637e-06 | 0.025878361638228 | -17.5735294117647 |
|  | chr4 | 2450576 | 2450576 | * | 5.74232470400425e-06 | 0.0326306195840953 | -5.11111111111111 |
|  | chr4 | 3632812 | 3632812 | * | 1.41927132446684e-05 | 0.0445180990564497 | -5.60540184453229 |
|  | chr4 | 3974783 | 3974783 | * | 2.00196325673458e-05 | 0.0487725967640558 | -8.79506110099427 |
|  | chr4 | 7042711 | 7042711 | * | 5.44007306061874e-06 | 0.0318323686811784 | -2.88132994852634 |
|  | chr4 | 7250842 | 7250842 | * | 1.66441963535253e-05 | 0.0464775741982698 | -7.6944971537002 |
|  | chr4 | 9101137 | 9101137 | * | 9.9163046333601e-06 | 0.038600331076739 | -3.70619570496947 |
|  | chr4 | 10099777 | 10099777 | * | 1.68309200751904e-06 | 0.0224030206914996 | 14.7130425774371 |
|  | chr4 | 34039349 | 34039349 | * | 1.97571809833578e-07 | 0.00944386277881804 | -4.26252276342177 |
|  | chr4 | 35302480 | 35302480 | * | 1.31717117840733e-05 | 0.0438657949404395 | -5.21555732695629 |
|  | chr4 | 38001036 | 38001036 | * | 1.19099683433329e-06 | 0.0195973483183763 | -3.46153846153846 |
|  | chr4 | 39980036 | 39980036 | * | 1.94649038400719e-05 | 0.0484073443519774 | 4.25770308123249 |
|  | chr4 | 40148074 | 40148074 | * | 1.26731237596773e-05 | 0.0430282910656179 | -2.30414746543779 |
|  | chr4 | 40878074 | 40878074 | * | 8.29091160007926e-06 | 0.0372521086012845 | 4.8625185392255 |
|  | chr4 | 41316842 | 41316842 | * | 2.05893330537927e-07 | 0.00947024632274357 | 17.6508590232774 |
|  | chr4 | 43994927 | 43994927 | * | 5.71713727181363e-06 | 0.0326306195840953 | -5.85514085514086 |
|  | chr4 | 47520729 | 47520729 | * | 6.67399369080332e-06 | 0.0339660578028265 | 3.00566052778443 |
|  | chr4 | 56269187 | 56269187 | * | 4.69355246500136e-07 | 0.0133044857153906 | -8.29597168380246 |
|  | chr4 | 58172342 | 58172342 | * | 9.69184673898045e-06 | 0.0381570098837087 | 3.36493480940736 |
|  | chr4 | 66100010 | 66100010 | * | 6.39529011526386e-06 | 0.0335275766188563 | 9.7398076839403 |
|  | chr4 | 77571091 | 77571091 | * | 1.09285713387145e-05 | 0.0401362202903679 | -7.62057546797522 |
|  | chr4 | 87361594 | 87361594 | * | 2.07751470595641e-05 | 0.0494100288284471 | -6.19984264358773 |
|  | chr4 | 90127877 | 90127877 | * | 1.83623361348594e-05 | 0.0473326884493546 | 3.00925925925926 |
|  | chr4 | 95271518 | 95271518 | * | 2.20420617989365e-06 | 0.0251778165859805 | 4.13823659725299 |
|  | chr4 | 98199880 | 98199880 | * | 4.64780496045414e-06 | 0.0308169634740055 | -5.6270182274292 |
|  | chr4 | 116981229 | 116981229 | * | 1.81694317508504e-05 | 0.0473326884493546 | 10.0351935646053 |
|  | chr4 | 119054265 | 119054265 | * | 1.40054505501823e-05 | 0.0445180990564497 | -6.23809523809524 |
|  | chr4 | 121870249 | 121870249 | * | 1.49536875062988e-05 | 0.0451161265302971 | 2.91060291060291 |
|  | chr4 | 122228156 | 122228156 | * | 3.33682910274524e-06 | 0.0290437810662865 | 2.59067357512953 |
|  | chr4 | 132522654 | 132522654 | * | 9.74024332439727e-06 | 0.0381745817553631 | 6.00816336846361 |
|  | chr4 | 139636942 | 139636942 | * | 1.4849326661254e-05 | 0.0449682251514597 | -4.4422198264798 |
|  | chr4 | 143912071 | 143912071 | * | 9.4476263062725e-06 | 0.0380630407857723 | 16.7145135566188 |
|  | chr4 | 146640038 | 146640038 | * | 5.36117076357088e-07 | 0.0143256643071847 | -10.9482259531883 |
|  | chr4 | 147712510 | 147712510 | * | 1.44113901660986e-05 | 0.0446291578551067 | 13.8972255208896 |
|  | chr4 | 150514602 | 150514602 | * | 1.32551304510322e-08 | 0.00724041545745036 | -6.06185262536126 |
|  | chr4 | 158769501 | 158769501 | * | 1.96317655645623e-07 | 0.00944386277881804 | -6.35593220338983 |
|  | chr4 | 177066165 | 177066165 | * | 4.75792484655076e-06 | 0.0310406905817438 | 6.53061224489796 |
|  | chr4 | 180242149 | 180242149 | * | 1.83502383395295e-05 | 0.0473326884493546 | -7.1461031189618 |
|  | chr4 | 181917794 | 181917794 | * | 8.11634317184426e-06 | 0.0370703970092214 | 6.69925970843124 |
|  | chr4 | 186120520 | 186120520 | * | 1.94798406130203e-05 | 0.0484073443519774 | 3.09613919463835 |
|  | chr5 | 111734 | 111734 | * | 3.22829407096602e-06 | 0.0287221794777653 | -9.04543568917708 |
|  | chr5 | 171093 | 171093 | * | 1.84423852619323e-05 | 0.0473326884493546 | -4.99437751004016 |
|  | chr5 | 271657 | 271657 | * | 1.41511879159022e-05 | 0.0445180990564497 | 3.93939393939394 |
|  | chr5 | 476429 | 476429 | * | 3.68296596880991e-06 | 0.0293699201736525 | -7.7671913835957 |
|  | chr5 | 565920 | 565920 | * | 1.40660957481367e-05 | 0.0445180990564497 | -13.4341607759329 |
|  | chr5 | 633586 | 633586 | * | 5.03549567634941e-06 | 0.0310952225336125 | -8.11878427363139 |
|  | chr5 | 847398 | 847398 | * | 1.11107442017301e-05 | 0.0401362202903679 | 3.70992532268967 |
|  | chr5 | 1186822 | 1186822 | * | 1.3579916842679e-05 | 0.0440049293460441 | -19.2008185073609 |
|  | chr5 | 1343478 | 1343478 | * | 2.04634574692788e-07 | 0.00947024632274357 | -6.75847881888187 |
|  | chr5 | 1445154 | 1445154 | * | 4.45795274867395e-06 | 0.030682714293622 | 8.23311405540628 |
|  | chr5 | 2124567 | 2124567 | * | 1.70885707864129e-06 | 0.022517951496672 | -7.02401223912006 |
|  | chr5 | 2417453 | 2417453 | * | 1.70393832366718e-05 | 0.0468130072138101 | -7.25444814118328 |
|  | chr5 | 2739089 | 2739089 | * | 5.68851507538367e-08 | 0.00866245804981213 | -7.73827275036725 |
|  | chr5 | 7394405 | 7394405 | * | 3.9412136537688e-07 | 0.0121617970927997 | -8.51940671476361 |
|  | chr5 | 10333050 | 10333050 | * | 1.43012498163998e-05 | 0.0445180990564497 | -7.68892466866437 |
|  | chr5 | 14219588 | 14219588 | * | 9.61024178592992e-06 | 0.0381570098837087 | 8.1384174223049 |
|  | chr5 | 17138886 | 17138886 | * | 3.99749509448409e-06 | 0.0300127101341861 | 7.32856866911339 |
|  | chr5 | 21577284 | 21577284 | * | 4.95101869571018e-06 | 0.0310952225336125 | -5.36821412369575 |
|  | chr5 | 22806555 | 22806555 | * | 4.11952968514078e-06 | 0.0303068913296594 | 5.87100623336884 |
|  | chr5 | 24672785 | 24672785 | * | 1.42904643133164e-05 | 0.0445180990564497 | -6.89561385006495 |
|  | chr5 | 36912594 | 36912594 | * | 9.67575951300402e-06 | 0.0381570098837087 | 2.82608695652173 |
|  | chr5 | 43746039 | 43746039 | * | 9.22814435669283e-06 | 0.0380630407857723 | -7.20192307692308 |
|  | chr5 | 43749447 | 43749447 | * | 1.38920674943904e-06 | 0.0209048346176606 | -9.91135526562207 |
|  | chr5 | 55226603 | 55226603 | * | 2.00912217052534e-05 | 0.0487725967640558 | -2.31660231660232 |
|  | chr5 | 58656006 | 58656006 | * | 7.77750077721757e-06 | 0.036427241008796 | 3.54962363581726 |
|  | chr5 | 68351000 | 68351000 | * | 1.15079487472179e-06 | 0.0193474967675106 | -6.69634340222575 |
|  | chr5 | 77351122 | 77351122 | * | 1.08051005346512e-05 | 0.0400638933557008 | -8.63210696306455 |
|  | chr5 | 82973387 | 82973387 | * | 1.01858545414852e-08 | 0.00724041545745036 | -5.55555555555556 |
|  | chr5 | 83080499 | 83080499 | * | 4.67540692348461e-06 | 0.0308169634740055 | -3.73697032839245 |
|  | chr5 | 96618554 | 96618554 | * | 1.67960748763288e-05 | 0.0465779040949594 | -3.0373831775701 |
|  | chr5 | 119818688 | 119818688 | * | 1.01136782465282e-05 | 0.038905815221874 | 3.36814265989926 |
|  | chr5 | 121851253 | 121851253 | * | 1.68280725216478e-05 | 0.0465779040949594 | -5.07265334765573 |
|  | chr5 | 126834621 | 126834621 | * | 1.55717775677138e-05 | 0.0454391445067938 | -3.86970859411805 |
|  | chr5 | 132823020 | 132823020 | * | 2.99618803369938e-07 | 0.0102873989537574 | 10.8765652951699 |
|  | chr5 | 138484299 | 138484299 | * | 8.94458909151898e-06 | 0.0376596861566517 | -3.1055900621118 |
|  | chr5 | 139224132 | 139224132 | * | 3.43605134616813e-07 | 0.0114744440037869 | 3.94736842105263 |
|  | chr5 | 139520107 | 139520107 | * | 3.38358139604992e-06 | 0.0290437810662865 | -18.7891573967523 |
|  | chr5 | 140665768 | 140665768 | * | 5.97548132901775e-06 | 0.0328824397860396 | -10.7685627442466 |
|  | chr5 | 141758785 | 141758785 | * | 1.17428718943358e-07 | 0.00890507499086869 | 6.20353304201959 |
|  | chr5 | 150714525 | 150714525 | * | 2.52673087176424e-06 | 0.0266987547411388 | 4.04917662982178 |
|  | chr5 | 154687442 | 154687442 | * | 2.05152835112943e-05 | 0.0490792825012072 | 4.2397501543378 |
|  | chr5 | 161213729 | 161213729 | * | 1.58936743488021e-05 | 0.0455833711863926 | 5.85914756221725 |
|  | chr5 | 170642169 | 170642169 | * | 5.00652448492326e-06 | 0.0310952225336125 | -6.97894520905599 |
|  | chr5 | 171373835 | 171373835 | * | 7.64634364538508e-08 | 0.00890507499086869 | 6.5625 |
|  | chr5 | 172112662 | 172112662 | * | 9.58938754948849e-06 | 0.0381570098837087 | -12.7928159626224 |
|  | chr5 | 174904394 | 174904394 | * | 2.77659498218349e-06 | 0.0274210684298071 | 9.51840753424658 |
|  | chr5 | 178287585 | 178287585 | * | 8.65947962155877e-06 | 0.0372521086012845 | 4.77798584938924 |
|  | chr5 | 180644996 | 180644996 | * | 4.38959480729511e-06 | 0.030682714293622 | -4.63768115942029 |
|  | chr6 | 1076410 | 1076410 | * | 6.51719154859814e-07 | 0.0146149559616363 | -8.23470992303237 |
|  | chr6 | 1076515 | 1076515 | * | 2.42857734399372e-07 | 0.00987832060906468 | -17.5225106880986 |
|  | chr6 | 5297579 | 5297579 | * | 6.00821903464483e-06 | 0.0329166188805524 | 2.70039778145609 |
|  | chr6 | 5393452 | 5393452 | * | 1.83189943415665e-05 | 0.0473326884493546 | -4.33709316015344 |
|  | chr6 | 7150972 | 7150972 | * | 8.35953001642411e-07 | 0.0177206189586943 | -5.8195606201425 |
|  | chr6 | 7345789 | 7345789 | * | 1.24770168523035e-05 | 0.0427795317809314 | 3.82513661202186 |
|  | chr6 | 7541356 | 7541356 | * | 5.93110503016461e-06 | 0.0328824397860396 | -5.1283970951561 |
|  | chr6 | 20345745 | 20345745 | * | 5.18351793926734e-06 | 0.0312632216132437 | -5.49443244141599 |
|  | chr6 | 31631854 | 31631854 | * | 1.23987986260462e-05 | 0.0425712226932395 | 5.01320757774645 |
|  | chr6 | 31932898 | 31932898 | * | 1.61210609670443e-05 | 0.0455833711863926 | -7.63739884100086 |
|  | chr6 | 32049395 | 32049395 | * | 1.85737062000458e-06 | 0.0236445123895326 | 8.00150840095531 |
|  | chr6 | 33552828 | 33552828 | * | 9.52392872602966e-06 | 0.0380645985342247 | 3.2258064516129 |
|  | chr6 | 37305800 | 37305800 | * | 4.02692610255135e-06 | 0.0300127101341861 | -4.56592476897045 |
|  | chr6 | 41547513 | 41547513 | * | 1.32829051726699e-05 | 0.0439956890457087 | 3.34261838440111 |
|  | chr6 | 42542444 | 42542444 | * | 1.42413015165083e-05 | 0.0445180990564497 | 3.75467922478869 |
|  | chr6 | 52459926 | 52459926 | * | 2.04500280542204e-05 | 0.0490676282382614 | 4.27412962624231 |
|  | chr6 | 55038611 | 55038611 | * | 1.89664711099988e-05 | 0.0479130796637049 | 7.58868648130393 |
|  | chr6 | 85325575 | 85325575 | * | 1.60739909363947e-05 | 0.0455833711863926 | -13.2635797319528 |
|  | chr6 | 98948124 | 98948124 | * | 1.78485590760476e-05 | 0.0472518712534723 | -3.24825986078886 |
|  | chr6 | 103082893 | 103082893 | * | 6.24666355235746e-06 | 0.0330331766726775 | -6.62849326825081 |
|  | chr6 | 107848574 | 107848574 | * | 1.32581290420215e-05 | 0.0439956890457087 | 2.81385281385281 |
|  | chr6 | 110414444 | 110414444 | * | 6.17531624637393e-06 | 0.0330331766726775 | 6.0273920739037 |
|  | chr6 | 110874821 | 110874821 | * | 1.44691611418559e-05 | 0.0447055267258755 | -2.26480836236934 |
|  | chr6 | 118894791 | 118894791 | * | 6.66360036320256e-08 | 0.00890507499086869 | 4.52127659574468 |
|  | chr6 | 129209778 | 129209778 | * | 1.68283774576295e-05 | 0.0465779040949594 | -4.79196729196729 |
|  | chr6 | 135140741 | 135140741 | * | 1.988270633491e-05 | 0.0486643610587646 | -3.7771139213916 |
|  | chr6 | 135279229 | 135279229 | * | 1.14728041898179e-05 | 0.040651447010847 | -3.44642009927842 |
|  | chr6 | 136376319 | 136376319 | * | 1.28224679543002e-05 | 0.0433070426297305 | -6.04156095760875 |
|  | chr6 | 136376378 | 136376378 | * | 2.4772400898767e-07 | 0.00987832060906468 | -8.44033555897963 |
|  | chr6 | 138482307 | 138482307 | * | 4.88953695752517e-06 | 0.0310406905817438 | 4.82632431459596 |
|  | chr6 | 144196458 | 144196458 | * | 8.35814895765487e-08 | 0.00890507499086869 | 10.6651742884554 |
|  | chr6 | 147488127 | 147488127 | * | 1.07214200542469e-05 | 0.0399184503837769 | -4.09652257643417 |
|  | chr6 | 151323277 | 151323277 | * | 1.37809462368368e-06 | 0.0209048346176606 | -8.73939393939393 |
|  | chr6 | 158214994 | 158214994 | * | 3.89271487330447e-06 | 0.0298414787713016 | 3.30411656644487 |
|  | chr6 | 163714463 | 163714463 | * | 2.46712225642852e-06 | 0.0262633459760735 | -3.73327622776962 |
|  | chr6 | 166307307 | 166307307 | * | 1.25096753953162e-05 | 0.0428312661580684 | 3.58694310749105 |
|  | chr6 | 167157736 | 167157736 | * | 3.72507928206624e-06 | 0.0294073719213501 | 6.3064205557166 |
|  | chr7 | 195971 | 195971 | * | 2.06931190460258e-05 | 0.0492630011076747 | 4.67625899280576 |
|  | chr7 | 1369199 | 1369199 | * | 2.01449768537478e-05 | 0.0487725967640558 | 2.82817869415808 |
|  | chr7 | 1477647 | 1477647 | * | 3.31299024914281e-06 | 0.0290437810662865 | -7.28070175438597 |
|  | chr7 | 1496261 | 1496261 | * | 8.62967969733719e-06 | 0.0372521086012845 | 2.4822695035461 |
|  | chr7 | 1745125 | 1745125 | * | 5.00762375671061e-06 | 0.0310952225336125 | -4.10298920937219 |
|  | chr7 | 2679572 | 2679572 | * | 5.61252643752414e-06 | 0.032345427716124 | -7.56218905472636 |
|  | chr7 | 3988502 | 3988502 | * | 3.89826857887936e-07 | 0.0121617970927997 | -8.34183037927217 |
|  | chr7 | 4771548 | 4771548 | * | 2.0494163226066e-05 | 0.0490792825012072 | -6.65784752550604 |
|  | chr7 | 5240563 | 5240563 | * | 9.12532271040846e-06 | 0.0380209070549651 | 2.5440313111546 |
|  | chr7 | 6061159 | 6061159 | * | 1.43186479470912e-05 | 0.0445180990564497 | -5.23753623535612 |
|  | chr7 | 24735523 | 24735523 | * | 9.21113977217398e-06 | 0.0380630407857723 | 4.38287648463236 |
|  | chr7 | 27340536 | 27340536 | * | 2.87959318809074e-06 | 0.0274211735986337 | 5.45921544209215 |
|  | chr7 | 29645833 | 29645833 | * | 2.77057842287881e-06 | 0.0274210684298071 | -6.83507421660253 |
|  | chr7 | 31507074 | 31507074 | * | 1.89518976045923e-05 | 0.0479130796637049 | 3.48484150483506 |
|  | chr7 | 32956789 | 32956789 | * | 1.81481372079926e-05 | 0.0473326884493546 | -15.4554491707215 |
|  | chr7 | 40044660 | 40044660 | * | 4.06278918471029e-06 | 0.0300127101341861 | 2.59816620452937 |
|  | chr7 | 40118749 | 40118749 | * | 1.10454986184654e-05 | 0.0401362202903679 | 2.41635687732342 |
|  | chr7 | 44080830 | 44080830 | * | 1.63039670589931e-05 | 0.0457370741369463 | 8.49856796651245 |
|  | chr7 | 44145930 | 44145930 | * | 1.35828661187998e-05 | 0.0440049293460441 | -15.2565609087348 |
|  | chr7 | 44152510 | 44152510 | * | 1.42852821067316e-05 | 0.0445180990564497 | -5.52763819095478 |
|  | chr7 | 44706439 | 44706439 | * | 4.85988099562327e-08 | 0.00866245804981213 | 5.70047542842213 |
|  | chr7 | 48008120 | 48008120 | * | 2.16775074551843e-06 | 0.0251778165859805 | -3.98259287663415 |
|  | chr7 | 49670342 | 49670342 | * | 1.13622640221628e-05 | 0.0403771468111895 | 4.41447563777824 |
|  | chr7 | 50899049 | 50899049 | * | 4.16201266959098e-07 | 0.0125260241601245 | -7.634805143028 |
|  | chr7 | 55004699 | 55004699 | * | 7.07854824441033e-06 | 0.0349354795451427 | 10.7496214033317 |
|  | chr7 | 56078387 | 56078387 | * | 8.84110344992603e-06 | 0.0374335718834972 | 7.26159521745894 |
|  | chr7 | 64499628 | 64499628 | * | 1.53828546090889e-05 | 0.045146389983925 | -2.70833333333333 |
|  | chr7 | 66123774 | 66123774 | * | 1.74541946768827e-05 | 0.0469912079857756 | 3.52783417299547 |
|  | chr7 | 66376791 | 66376791 | * | 8.11233067032639e-07 | 0.0173474506019032 | -6.80272108843537 |
|  | chr7 | 66960967 | 66960967 | * | 1.88702203990769e-07 | 0.00938805788263315 | -4.3975893789144 |
|  | chr7 | 68748043 | 68748043 | * | 3.65797276005563e-06 | 0.0293699201736525 | 4.47692957632366 |
|  | chr7 | 71968671 | 71968671 | * | 1.70215694396514e-05 | 0.0468130072138101 | -7.7922077922078 |
|  | chr7 | 72823350 | 72823350 | * | 2.80723541634338e-06 | 0.0274210684298071 | -5.65353304023671 |
|  | chr7 | 73724494 | 73724494 | * | 1.33503590998169e-05 | 0.0440049293460441 | -7.08589405911618 |
|  | chr7 | 73956359 | 73956359 | * | 3.86157288154778e-06 | 0.0298414787713016 | 3.75335120643432 |
|  | chr7 | 75538820 | 75538820 | * | 1.5945377617293e-05 | 0.0455833711863926 | -5.25676536370773 |
|  | chr7 | 98193641 | 98193641 | * | 2.01012551322611e-06 | 0.0241391516940067 | -3.26086956521739 |
|  | chr7 | 100018623 | 100018623 | * | 4.52212243278697e-06 | 0.030682714293622 | -4.43760718248765 |
|  | chr7 | 100081898 | 100081898 | * | 1.71291782590562e-05 | 0.0468130072138101 | -3.0690537084399 |
|  | chr7 | 100421796 | 100421796 | * | 1.99367765607684e-05 | 0.0487477583128015 | -2.81995661605207 |
|  | chr7 | 100569690 | 100569690 | * | 1.93116317394487e-06 | 0.0238188196676852 | -3.99423868312757 |
|  | chr7 | 101207165 | 101207165 | * | 1.0972928259603e-05 | 0.0401362202903679 | -7.27534859700465 |
|  | chr7 | 102283109 | 102283109 | * | 2.01983635758897e-05 | 0.0487725967640558 | 4.55287943558197 |
|  | chr7 | 102308905 | 102308905 | * | 1.40770422360214e-05 | 0.0445180990564497 | -4 |
|  | chr7 | 105877414 | 105877414 | * | 1.19073415495062e-05 | 0.0413534866384187 | -7.3202614379085 |
|  | chr7 | 122307026 | 122307026 | * | 1.07224340940385e-05 | 0.0399184503837769 | -9.38545943894276 |
|  | chr7 | 129331614 | 129331614 | * | 1.47813902295198e-05 | 0.0449484016985218 | 3.43915343915344 |
|  | chr7 | 131923277 | 131923277 | * | 9.93595442425545e-07 | 0.0185826556422598 | 6.59496198717725 |
|  | chr7 | 141735809 | 141735809 | * | 1.60888226319879e-05 | 0.0455833711863926 | 3.70946455505279 |
|  | chr7 | 149029052 | 149029052 | * | 9.49285537354208e-08 | 0.00890507499086869 | -3.44234079173838 |
|  | chr7 | 149945789 | 149945789 | * | 5.34348952842944e-06 | 0.0316619429790608 | -6.45693361630069 |
|  | chr7 | 155097133 | 155097133 | * | 5.00343556220754e-06 | 0.0310952225336125 | -4.07488220598932 |
|  | chr7 | 157303135 | 157303135 | * | 1.73650452607013e-05 | 0.0469912079857756 | 11.401020986954 |
|  | chr7 | 157416926 | 157416926 | * | 1.42187812781842e-05 | 0.0445180990564497 | -3.51750101750101 |
|  | chr7 | 157467764 | 157467764 | * | 2.01043260330232e-05 | 0.0487725967640558 | 3.49222111883482 |
|  | chr8 | 230786 | 230786 | * | 4.6258415349718e-07 | 0.0132668155633019 | 3.60934182590233 |
|  | chr8 | 1247088 | 1247088 | * | 2.53072118805581e-07 | 0.00987832060906468 | -9.80570310315907 |
|  | chr8 | 1842431 | 1842431 | * | 8.85714310810133e-07 | 0.0179931533669612 | -5.91645984818798 |
|  | chr8 | 2561820 | 2561820 | * | 1.35897064785254e-05 | 0.0440049293460441 | -7.49914000687995 |
|  | chr8 | 10404591 | 10404591 | * | 3.38259739569329e-08 | 0.00824603504446799 | 3.61663652802894 |
|  | chr8 | 10914189 | 10914189 | * | 5.09828293550158e-06 | 0.0310952225336125 | -5.83333333333333 |
|  | chr8 | 11700742 | 11700742 | * | 4.6268645278936e-06 | 0.0308169634740055 | -5.4179258619668 |
|  | chr8 | 13154412 | 13154412 | * | 4.44316524637223e-06 | 0.030682714293622 | 5.26315789473685 |
|  | chr8 | 17043182 | 17043182 | * | 8.53662190073412e-06 | 0.0372521086012845 | 2.6 |
|  | chr8 | 22141829 | 22141829 | * | 1.78848342676745e-05 | 0.0472518712534723 | -2.99539170506912 |
|  | chr8 | 26448522 | 26448522 | * | 1.72301271431623e-05 | 0.0468415980941163 | 4.31893687707641 |
|  | chr8 | 34019045 | 34019045 | * | 3.60412589015908e-06 | 0.0293113670780731 | -7.07834858081163 |
|  | chr8 | 35533803 | 35533803 | * | 3.00240736374512e-06 | 0.028042967923392 | 8.97056920764783 |
|  | chr8 | 37874515 | 37874515 | * | 2.8345886729459e-06 | 0.0274210684298071 | -13.9556301180225 |
|  | chr8 | 41767188 | 41767188 | * | 1.75013010808077e-05 | 0.0470389820853072 | 2.64900662251656 |
|  | chr8 | 47574711 | 47574711 | * | 1.28795790216494e-05 | 0.0433070426297305 | -12.9534350814994 |
|  | chr8 | 47578201 | 47578201 | * | 5.30694389647217e-06 | 0.0315540787947066 | 2.61865793780688 |
|  | chr8 | 50962666 | 50962666 | * | 9.41911715483724e-06 | 0.0380630407857723 | -4.07066052227342 |
|  | chr8 | 56314122 | 56314122 | * | 1.34488848338254e-05 | 0.0440049293460441 | -3.31632653061225 |
|  | chr8 | 66961788 | 66961788 | * | 1.28447492596599e-05 | 0.0433070426297305 | 4.07279292400589 |
|  | chr8 | 71842297 | 71842297 | * | 1.67937543452531e-05 | 0.0465779040949594 | -4.66404058736182 |
|  | chr8 | 80218969 | 80218969 | * | 7.23206967470753e-06 | 0.0351971330669854 | -3.53469825933594 |
|  | chr8 | 86636400 | 86636400 | * | 7.23352771763827e-06 | 0.0351971330669854 | -5.18047489248743 |
|  | chr8 | 87912493 | 87912493 | * | 5.89034855809602e-06 | 0.0328824397860396 | 3.97262047914162 |
|  | chr8 | 95133875 | 95133875 | * | 1.15146630363893e-05 | 0.0406815047625729 | 2.4896265560166 |
|  | chr8 | 96312734 | 96312734 | * | 1.07419381436722e-05 | 0.0399184503837769 | -8.47955345225094 |
|  | chr8 | 102509642 | 102509642 | * | 2.81379303759428e-06 | 0.0274210684298071 | 4.37017994858612 |
|  | chr8 | 108365444 | 108365444 | * | 2.06655219696314e-05 | 0.0492453934292982 | -3.15457413249212 |
|  | chr8 | 113372791 | 113372791 | * | 9.84562535631169e-06 | 0.0384639235133978 | -6.20073568050447 |
|  | chr8 | 118477741 | 118477741 | * | 6.08740185196045e-07 | 0.0144075326810445 | -4.49932502183753 |
|  | chr8 | 121948302 | 121948302 | * | 1.08303801248048e-05 | 0.0400638933557008 | 10.8965346125217 |
|  | chr8 | 129965524 | 129965524 | * | 1.00427738098367e-05 | 0.0388604654344305 | -2.86885245901639 |
|  | chr8 | 133029916 | 133029916 | * | 2.10994815306586e-05 | 0.0498410235317558 | 3.05826030509575 |
|  | chr8 | 135249602 | 135249602 | * | 1.2358015278757e-07 | 0.00890507499086869 | -5.9760978666471 |
|  | chr8 | 140651760 | 140651760 | * | 9.99624499313239e-07 | 0.0185826556422598 | -4.09638554216868 |
|  | chr8 | 141157078 | 141157078 | * | 1.06887892675583e-05 | 0.0399184503837769 | -7.73193868209353 |
|  | chr8 | 143470512 | 143470512 | * | 4.65853288503133e-06 | 0.0308169634740055 | 3.85542168674698 |
|  | chr8 | 143808857 | 143808857 | * | 1.55902696589931e-05 | 0.0454391445067938 | -10.5931105931106 |
|  | chr8 | 144108684 | 144108684 | * | 1.60472634741944e-05 | 0.0455833711863926 | 11.2458596003847 |
|  | chr8 | 144267016 | 144267016 | * | 1.76019346468087e-05 | 0.0471534938979854 | 13.4934368063202 |
|  | chr8 | 144675767 | 144675767 | * | 7.67310534043788e-06 | 0.0361593579075616 | -3.59712230215827 |
|  | chr9 | 2748953 | 2748953 | * | 1.50099860381693e-05 | 0.0451189213522599 | -10.2439493461152 |
|  | chr9 | 8253902 | 8253902 | * | 3.15751486610667e-06 | 0.0286146204659006 | -5.93001751995328 |
|  | chr9 | 10613180 | 10613180 | * | 2.82517692368728e-06 | 0.0274210684298071 | -4.79117415287628 |
|  | chr9 | 12776290 | 12776290 | * | 1.03264108965378e-05 | 0.0393467973402204 | -2.57352941176471 |
|  | chr9 | 13506300 | 13506300 | * | 1.76763287370559e-07 | 0.00916830553644203 | 7.48187470815208 |
|  | chr9 | 23824579 | 23824579 | * | 8.16980913683262e-06 | 0.0370703970092214 | -4.6875 |
|  | chr9 | 33897227 | 33897227 | * | 1.98057441882041e-05 | 0.0486224437851043 | -4.20420420420421 |
|  | chr9 | 35184315 | 35184315 | * | 2.55287059454486e-07 | 0.00987832060906468 | -6.45994832041343 |
|  | chr9 | 35853601 | 35853601 | * | 1.06955019003407e-05 | 0.0399184503837769 | -5.7941513761468 |
|  | chr9 | 37027171 | 37027171 | * | 1.60784063166984e-05 | 0.0455833711863926 | 2.81925262328398 |
|  | chr9 | 37027320 | 37027320 | * | 5.07726504626878e-06 | 0.0310952225336125 | 5.18211300815007 |
|  | chr9 | 71708014 | 71708014 | * | 1.59575836335139e-05 | 0.0455833711863926 | 3.16626219602794 |
|  | chr9 | 81695594 | 81695594 | * | 1.91611639251863e-05 | 0.0480849487724047 | 2.57425742574258 |
|  | chr9 | 82141307 | 82141307 | * | 1.04925903690297e-07 | 0.00890507499086869 | -8.31530674050359 |
|  | chr9 | 87473998 | 87473998 | * | 1.88001867704436e-06 | 0.0236445123895326 | -6.80272108843537 |
|  | chr9 | 87657965 | 87657965 | * | 1.56009695789758e-05 | 0.0454391445067938 | 4.39076409495549 |
|  | chr9 | 88535990 | 88535990 | * | 1.36666120600885e-05 | 0.0440616206749897 | 10.1797430289776 |
|  | chr9 | 93826475 | 93826475 | * | 1.53435725533358e-05 | 0.045130930916365 | -4.15335266177287 |
|  | chr9 | 95508664 | 95508664 | * | 1.75866208385959e-05 | 0.0471534938979854 | -3.10262529832936 |
|  | chr9 | 95654305 | 95654305 | * | 2.01623058084304e-05 | 0.0487725967640558 | -5.6673899616854 |
|  | chr9 | 98217183 | 98217183 | * | 5.79130408866718e-06 | 0.0326804094071279 | -11.3012798858987 |
|  | chr9 | 100685704 | 100685704 | * | 6.43425393714543e-06 | 0.033554488025128 | -5.42698222270694 |
|  | chr9 | 108063188 | 108063188 | * | 7.0005344690645e-06 | 0.034843158469973 | -3.13136672325977 |
|  | chr9 | 112716717 | 112716717 | * | 2.10451955081377e-05 | 0.0497610075618031 | 3.67239352220579 |
|  | chr9 | 113410554 | 113410554 | * | 9.41792249184192e-06 | 0.0380630407857723 | -3.33333333333333 |
|  | chr9 | 114121968 | 114121968 | * | 2.3059293514756e-06 | 0.0255776349518986 | 5.60928433268859 |
|  | chr9 | 117505733 | 117505733 | * | 7.51833194602984e-06 | 0.0360079652024686 | -5.72426867888909 |
|  | chr9 | 119369749 | 119369749 | * | 2.82577522917479e-06 | 0.0274210684298071 | -5.79071320608809 |
|  | chr9 | 120953213 | 120953213 | * | 1.84454779085607e-05 | 0.0473326884493546 | 7.86094477711243 |
|  | chr9 | 124870463 | 124870463 | * | 1.91725799135351e-05 | 0.0480849487724047 | -2.79569892473118 |
|  | chr9 | 130252958 | 130252958 | * | 1.36823747369401e-05 | 0.0440616206749897 | 4.4669195111673 |
|  | chr9 | 130547098 | 130547098 | * | 3.6052719066956e-07 | 0.0115642986305898 | 7.0414673046252 |
|  | chr9 | 132526316 | 132526316 | * | 8.81090605427437e-06 | 0.0374335718834972 | 3.5346687211094 |
|  | chr9 | 132846843 | 132846843 | * | 8.0420152972027e-06 | 0.037053924127198 | -6.7124223047568 |
|  | chr9 | 133161376 | 133161376 | * | 9.41685313910245e-06 | 0.0380630407857723 | 7.39060136650498 |
|  | chr9 | 133609229 | 133609229 | * | 6.45670071121249e-06 | 0.0335608289406844 | -7.63901418107026 |
|  | chr9 | 133652284 | 133652284 | * | 1.76901175498977e-05 | 0.0472318774384622 | -4.35815196985393 |
|  | chr9 | 134741186 | 134741186 | * | 1.02179888637505e-06 | 0.0186146105157064 | 3.6388384754991 |
|  | chr9 | 135590504 | 135590504 | * | 7.64123831619245e-06 | 0.0361593579075616 | 4.25591376384868 |
|  | chr9 | 135661041 | 135661041 | * | 1.20585115804303e-06 | 0.0195973483183763 | -5.75757575757576 |
|  | chr9 | 136008951 | 136008951 | * | 1.80892731131544e-05 | 0.0473326884493546 | 3.81528760137334 |
|  | chr9 | 136588323 | 136588323 | * | 1.64382366671462e-07 | 0.00890507499086869 | 5.80287410184317 |
|  | chr9 | 137044804 | 137044804 | * | 9.51954310260385e-06 | 0.0380645985342247 | -5.49132947976879 |
|  | chr9 | 137495925 | 137495925 | * | 1.10447961993812e-05 | 0.0401362202903679 | 2.91970802919708 |
|  | chr9 | 137811515 | 137811515 | * | 4.79531558514993e-06 | 0.0310406905817438 | -4.48113207547169 |
|  | chrX | 347195 | 347195 | * | 2.96012832567104e-06 | 0.0279695641473294 | 3.8235294117647 |
|  | chrX | 620392 | 620392 | * | 3.84503799824171e-06 | 0.0298414787713016 | -4.29184549356223 |
|  | chrX | 632968 | 632968 | * | 8.03456773581773e-07 | 0.0173332076142984 | 8.49324865058149 |
|  | chrX | 9715594 | 9715594 | * | 4.21470725780763e-06 | 0.0304426130789399 | -4.46361940298508 |
|  | chrX | 19658675 | 19658675 | * | 1.60239724923052e-06 | 0.0223141858609182 | -5.60225506515961 |
|  | chrX | 19731219 | 19731219 | * | 4.55624931773684e-06 | 0.030682714293622 | -6.66700625695099 |
|  | chrX | 21374886 | 21374886 | * | 9.98775034716204e-07 | 0.0185826556422598 | 11.8171088129496 |
|  | chrX | 22784204 | 22784204 | * | 1.01342911982655e-05 | 0.038905815221874 | -13.0360623781676 |
|  | chrX | 33053323 | 33053323 | * | 1.0442170059282e-06 | 0.0187043339204075 | 7.88905420504478 |
|  | chrX | 41505860 | 41505860 | * | 5.94668556555343e-07 | 0.0144075326810445 | 9.45267659097665 |
|  | chrX | 48919434 | 48919434 | * | 1.82491408749785e-05 | 0.0473326884493546 | 11.2230957770248 |
|  | chrX | 49270580 | 49270580 | * | 2.02108607779648e-05 | 0.0487725967640558 | 6.4569039203456 |
|  | chrX | 52816163 | 52816163 | * | 1.27629829972712e-06 | 0.0201016113850057 | -18.7491386201384 |
|  | chrX | 54098292 | 54098292 | * | 1.36175449527285e-05 | 0.0440049293460441 | -10.5222116013483 |
|  | chrX | 63438661 | 63438661 | * | 1.96925689096056e-05 | 0.0484911010319152 | -7.40721649484536 |
|  | chrX | 79245361 | 79245361 | * | 4.99487501689488e-08 | 0.00866245804981213 | -5 |
|  | chrX | 80810063 | 80810063 | * | 1.42776891166821e-05 | 0.0445180990564497 | -10.6637685097134 |
|  | chrX | 97089974 | 97089974 | * | 5.97260036419352e-06 | 0.0328824397860396 | 5.35446819426859 |
|  | chrX | 98380346 | 98380346 | * | 8.39969572320804e-06 | 0.0372521086012845 | -8.78453472838107 |
|  | chrX | 98693085 | 98693085 | * | 1.82459997663047e-05 | 0.0473326884493546 | 11.6753025367517 |
|  | chrX | 101418299 | 101418299 | * | 9.91734556065606e-07 | 0.0185826556422598 | 10.2729521357384 |
|  | chrX | 110423056 | 110423056 | * | 9.97242489818004e-06 | 0.03864959922597 | 6.72092279417364 |
|  | chrX | 117913583 | 117913583 | * | 1.68729234797885e-05 | 0.0466354978984192 | -7.92385884158036 |
|  | chrX | 118346226 | 118346226 | * | 7.21526101141511e-06 | 0.0351971330669854 | 10.4368932038835 |
|  | chrX | 119889177 | 119889177 | * | 1.50836129002909e-05 | 0.0451189213522599 | 4.3918918918919 |
|  | chrX | 129654069 | 129654069 | * | 5.52936343121862e-06 | 0.0320937682001976 | 11.0778022987333 |
|  | chrX | 129843490 | 129843490 | * | 1.45056851864791e-05 | 0.0447616433484372 | 15.9802102659246 |
|  | chrX | 129843527 | 129843527 | * | 6.26033321387104e-06 | 0.0330331766726775 | 16.091693541358 |
|  | chrX | 129984473 | 129984473 | * | 1.95879766088276e-05 | 0.0484431948255683 | -11.7459982780736 |
|  | chrX | 140826667 | 140826667 | * | 2.34883525101819e-06 | 0.025878361638228 | 6.55838546710992 |

***Supplementary table 3****. Table of significant DMLs identified in the moderate domain trial.*

|  | **chr** | **start** | **end** | **strand** | **pvalue** | **qvalue** | **meth.diff** |
| --- | --- | --- | --- | --- | --- | --- | --- |
|  | chr1 | 858514 | 858514 | * | 6.51207450708339e-06 | 0.0341324465221042 | -15.8008363648727 |
|  | chr1 | 930256 | 930256 | * | 1.6408899891584e-05 | 0.0478268132767551 | -5.08819650511045 |
|  | chr1 | 1040556 | 1040556 | * | 1.61843234173421e-06 | 0.0210703445532903 | -4.61095100864553 |
|  | chr1 | 1736268 | 1736268 | * | 1.52254745776303e-05 | 0.046434456775118 | 2.75228585144282 |
|  | chr1 | 1866676 | 1866676 | * | 7.01837860441855e-06 | 0.0351867937067003 | 3.2258064516129 |
|  | chr1 | 3018188 | 3018188 | * | 8.31276581226098e-06 | 0.0370029781436216 | -8.44424392811489 |
|  | chr1 | 3121986 | 3121986 | * | 7.15841006074118e-06 | 0.0354684397187285 | -3.91951418869622 |
|  | chr1 | 3205768 | 3205768 | * | 1.10874703862728e-05 | 0.0408887682689852 | 6.28045157456922 |
|  | chr1 | 6126253 | 6126253 | * | 2.24879613684098e-06 | 0.0242023535912919 | 9.62209692576185 |
|  | chr1 | 6419193 | 6419193 | * | 1.11951590311919e-06 | 0.0189576396111616 | 9.09519174615534 |
|  | chr1 | 6461809 | 6461809 | * | 8.80469276860303e-06 | 0.0381410312784402 | -4.28770740943744 |
|  | chr1 | 6703615 | 6703615 | * | 2.10572943870549e-06 | 0.023273252720545 | -4.87258687258687 |
|  | chr1 | 7690087 | 7690087 | * | 1.30681003999439e-05 | 0.0436480141097609 | -8.97199300484213 |
|  | chr1 | 8332027 | 8332027 | * | 1.39609603625688e-05 | 0.0448427563112155 | 10.4365079365079 |
|  | chr1 | 11736181 | 11736181 | * | 1.77253156937857e-05 | 0.049054173071187 | 4.38871473354232 |
|  | chr1 | 11806109 | 11806109 | * | 7.42514040619362e-06 | 0.0358886380697614 | 2.3728813559322 |
|  | chr1 | 15941962 | 15941962 | * | 1.21624017986506e-05 | 0.0424375115128073 | 5.17324119397256 |
|  | chr1 | 16182583 | 16182583 | * | 6.46494430037891e-06 | 0.0341068911473657 | -4.08098021817422 |
|  | chr1 | 16223075 | 16223075 | * | 8.9638322415666e-06 | 0.0384844115216733 | 3.95212973504588 |
|  | chr1 | 18633091 | 18633091 | * | 5.87466131113245e-06 | 0.0328689369263055 | -7.24778693528694 |
|  | chr1 | 21283034 | 21283034 | * | 1.57830849649423e-05 | 0.0471449739477541 | -4.43037974683544 |
|  | chr1 | 23926199 | 23926199 | * | 8.58558609570189e-07 | 0.0163972973909036 | 7.3862107321624 |
|  | chr1 | 28593501 | 28593501 | * | 5.86072846455805e-06 | 0.0328689369263055 | -2.57731958762887 |
|  | chr1 | 34745813 | 34745813 | * | 5.20568262010823e-06 | 0.0313575170167993 | 5.40719980265886 |
|  | chr1 | 37515022 | 37515022 | * | 9.82100499776006e-06 | 0.0397414035646381 | 2.52918287937743 |
|  | chr1 | 39922639 | 39922639 | * | 2.80021780058192e-06 | 0.0261031234884416 | 11.220946256316 |
|  | chr1 | 43411935 | 43411935 | * | 1.0429511053204e-05 | 0.0402474126567004 | 3.91705069124424 |
|  | chr1 | 46142420 | 46142420 | * | 8.0638222393328e-07 | 0.0163328485347103 | 4.17633410672854 |
|  | chr1 | 52475993 | 52475993 | * | 1.07308680259811e-05 | 0.0405187716477507 | -2.7113772722952 |
|  | chr1 | 53946270 | 53946270 | * | 1.05849929101872e-05 | 0.0402474126567004 | 2.8169014084507 |
|  | chr1 | 55039578 | 55039578 | * | 1.7871666860964e-05 | 0.0491802621659287 | -8.7398528184508 |
|  | chr1 | 58450492 | 58450492 | * | 2.90121171249008e-06 | 0.0261571757711947 | -8.50078247261347 |
|  | chr1 | 60818073 | 60818073 | * | 9.02025734021844e-06 | 0.0384844115216733 | -4.07944748308148 |
|  | chr1 | 62189093 | 62189093 | * | 5.05500358991742e-07 | 0.0137537616832543 | -4.15512465373962 |
|  | chr1 | 65241738 | 65241738 | * | 6.41623837018166e-06 | 0.034035603327331 | 3.8659793814433 |
|  | chr1 | 75615591 | 75615591 | * | 1.42709172363315e-05 | 0.0450629053763874 | 3.48027842227378 |
|  | chr1 | 77219450 | 77219450 | * | 6.57785040418083e-06 | 0.0342703714790988 | 3.61636386108021 |
|  | chr1 | 86791528 | 86791528 | * | 1.11280804717376e-05 | 0.0408887682689852 | -4.78079439493071 |
|  | chr1 | 86795000 | 86795000 | * | 9.34530874439055e-06 | 0.0389962445826831 | 4.67845659163988 |
|  | chr1 | 88666539 | 88666539 | * | 1.01555747205044e-05 | 0.0402474126567004 | -2.95275590551181 |
|  | chr1 | 89995598 | 89995598 | * | 7.85433479441395e-06 | 0.0365550330541403 | 8.26951906324703 |
|  | chr1 | 91910936 | 91910936 | * | 9.05466593331856e-06 | 0.0385345417437167 | -5.83696231084934 |
|  | chr1 | 92105175 | 92105175 | * | 8.6159313167372e-06 | 0.037585439420415 | -3.39943342776204 |
|  | chr1 | 99079322 | 99079322 | * | 1.66541317609083e-05 | 0.0479530277492557 | 4.21686746987952 |
|  | chr1 | 99971497 | 99971497 | * | 8.40862796191595e-07 | 0.0163328485347103 | 4.47638676663943 |
|  | chr1 | 109077055 | 109077055 | * | 4.89783408184636e-06 | 0.0313575170167993 | 4.24836601307189 |
|  | chr1 | 109656109 | 109656109 | * | 7.76720252752915e-06 | 0.0364983966694477 | 9.45285837579241 |
|  | chr1 | 112466354 | 112466354 | * | 9.66944688218465e-08 | 0.00755318238002048 | 5.0531914893617 |
|  | chr1 | 118194438 | 118194438 | * | 1.37711700443245e-05 | 0.0446273176508244 | 9.77240684793554 |
|  | chr1 | 151059826 | 151059826 | * | 9.67614108540317e-06 | 0.0396464900146826 | 8.12980827939919 |
|  | chr1 | 151069616 | 151069616 | * | 1.20363396132735e-06 | 0.0190538219820252 | -11.6152959602825 |
|  | chr1 | 151709034 | 151709034 | * | 3.5224771880922e-06 | 0.0277638687256849 | -7.70456146800232 |
|  | chr1 | 153886628 | 153886628 | * | 1.18495704296424e-05 | 0.0418825275394198 | 4.5016077170418 |
|  | chr1 | 155055753 | 155055753 | * | 8.20703699817831e-06 | 0.0367265471261412 | 4.61538461538461 |
|  | chr1 | 155062403 | 155062403 | * | 8.23549857073673e-06 | 0.0367265471261412 | 14.1100259118995 |
|  | chr1 | 156003206 | 156003206 | * | 9.82449971813537e-06 | 0.0397414035646381 | -3.65448504983389 |
|  | chr1 | 156030226 | 156030226 | * | 5.79911871903121e-06 | 0.0328689369263055 | 3.03030303030303 |
|  | chr1 | 156456079 | 156456079 | * | 1.22098650078633e-05 | 0.0424495632726324 | -3.74622898251092 |
|  | chr1 | 160205533 | 160205533 | * | 3.18938224588899e-06 | 0.0268166249280202 | -2.99295774647887 |
|  | chr1 | 162563652 | 162563652 | * | 7.79704668659597e-06 | 0.0364983966694477 | -4.45824571698697 |
|  | chr1 | 165769055 | 165769055 | * | 1.79179436565884e-05 | 0.0491944892733664 | 2.91970802919708 |
|  | chr1 | 167143024 | 167143024 | * | 1.76576654919121e-05 | 0.0489228654191022 | 3.65853658536586 |
|  | chr1 | 178915525 | 178915525 | * | 4.1873974518135e-06 | 0.0298232727614335 | 3.83693045563549 |
|  | chr1 | 185014830 | 185014830 | * | 9.79645021383048e-06 | 0.0397414035646381 | 2.87958115183246 |
|  | chr1 | 185354151 | 185354151 | * | 2.71173764352245e-07 | 0.0105912205929944 | -4.45989450296993 |
|  | chr1 | 197910588 | 197910588 | * | 2.90691934362575e-06 | 0.0261571757711947 | -10.5373728438744 |
|  | chr1 | 198984108 | 198984108 | * | 6.28691365142313e-06 | 0.0339063614727045 | 2.66666666666666 |
|  | chr1 | 205828904 | 205828904 | * | 1.29924781324745e-05 | 0.0435154751930969 | 3.07749576988156 |
|  | chr1 | 210047483 | 210047483 | * | 1.28667845385596e-05 | 0.0433342387071528 | -6.88172043010753 |
|  | chr1 | 217413025 | 217413025 | * | 6.19295439514044e-06 | 0.0335490627503777 | 5.26034756597569 |
|  | chr1 | 228160178 | 228160178 | * | 9.7455179366087e-07 | 0.0174807954033733 | 9.08167036215817 |
|  | chr1 | 237864615 | 237864615 | * | 1.46757961210295e-05 | 0.0457373169327578 | 9.11694883932506 |
|  | chr1 | 239431862 | 239431862 | * | 1.3074904072024e-06 | 0.0190538219820252 | -8.19880394375303 |
|  | chr1 | 240084128 | 240084128 | * | 1.49417019652886e-05 | 0.0462742867132491 | 7.92776921382955 |
|  | chr1 | 247426785 | 247426785 | * | 5.2617391358173e-06 | 0.0314205302796785 | -4.05348792445567 |
|  | chr10 | 291682 | 291682 | * | 1.24543110617729e-07 | 0.00816944669016107 | -9.89973821523338 |
|  | chr10 | 452831 | 452831 | * | 3.64291469747625e-07 | 0.0117501795376771 | -11.2538900221862 |
|  | chr10 | 5199612 | 5199612 | * | 3.68780595773278e-07 | 0.0117501795376771 | -4.53603889241175 |
|  | chr10 | 11852523 | 11852523 | * | 4.97543210324326e-06 | 0.0313575170167993 | 5.65849433209054 |
|  | chr10 | 13312335 | 13312335 | * | 5.49520094307944e-06 | 0.0322510100560176 | 3.52941176470588 |
|  | chr10 | 13892395 | 13892395 | * | 4.00905129026978e-06 | 0.0290040143512226 | -11.8980156791588 |
|  | chr10 | 15202450 | 15202450 | * | 1.63205479420026e-05 | 0.0476727536532516 | -3.49854227405247 |
|  | chr10 | 21393599 | 21393599 | * | 1.68517812585668e-05 | 0.0480106905549777 | 7.41350906095553 |
|  | chr10 | 21516316 | 21516316 | * | 1.93214116035162e-06 | 0.0226026073244803 | 8.43344438367063 |
|  | chr10 | 27325135 | 27325135 | * | 8.03761264456401e-06 | 0.0366541020429449 | 3.44827586206896 |
|  | chr10 | 43877868 | 43877868 | * | 5.91411170944244e-06 | 0.0329223099813284 | -6.49555330406394 |
|  | chr10 | 44865038 | 44865038 | * | 1.69783125963737e-05 | 0.0482254425991749 | 8.35275994165201 |
|  | chr10 | 47312432 | 47312432 | * | 6.52109451088777e-07 | 0.014757974310322 | 7.4980574980575 |
|  | chr10 | 49472467 | 49472467 | * | 4.0095449068895e-06 | 0.0290040143512226 | 4.31034482758621 |
|  | chr10 | 50683755 | 50683755 | * | 2.9435206055983e-06 | 0.0262052441014524 | 8.99908172635445 |
|  | chr10 | 54920275 | 54920275 | * | 4.62830018539578e-06 | 0.0313575170167993 | -5.95585337012219 |
|  | chr10 | 58924439 | 58924439 | * | 1.45630973285836e-05 | 0.045444576852039 | 4.67783000391696 |
|  | chr10 | 60752590 | 60752590 | * | 1.74003467036711e-05 | 0.0485151792386732 | -6.43939393939394 |
|  | chr10 | 63618494 | 63618494 | * | 1.0568188209469e-05 | 0.0402474126567004 | -5.1633829614093 |
|  | chr10 | 63661594 | 63661594 | * | 5.10437509720906e-06 | 0.0313575170167993 | 5.21134593993325 |
|  | chr10 | 64799790 | 64799790 | * | 1.0084868717924e-05 | 0.0402474126567004 | 3.34190231362468 |
|  | chr10 | 68528956 | 68528956 | * | 9.83432060137362e-06 | 0.0397414035646381 | -5.93752620545073 |
|  | chr10 | 68723388 | 68723388 | * | 1.22576950745301e-05 | 0.0424495632726324 | 4.87804878048781 |
|  | chr10 | 71297276 | 71297276 | * | 1.21217606627237e-05 | 0.0423567376902827 | 4.77323096039539 |
|  | chr10 | 72058073 | 72058073 | * | 7.22150205561993e-07 | 0.0157541361209436 | 5.13573353869851 |
|  | chr10 | 75111358 | 75111358 | * | 1.65691953420522e-05 | 0.0479364637584648 | -2.72108843537415 |
|  | chr10 | 78061347 | 78061347 | * | 8.34569748176183e-06 | 0.0370814039792811 | 7.94151104370082 |
|  | chr10 | 78271975 | 78271975 | * | 1.06039729720474e-05 | 0.0402474126567004 | -6.77914005669205 |
|  | chr10 | 80241266 | 80241266 | * | 4.04903979867407e-06 | 0.0291811767640566 | 5.57889822595705 |
|  | chr10 | 84348138 | 84348138 | * | 2.70045771433455e-06 | 0.0261031234884416 | 5.82191780821918 |
|  | chr10 | 86971805 | 86971805 | * | 1.44834582992592e-05 | 0.0452579147051184 | 4.85013183549355 |
|  | chr10 | 92790968 | 92790968 | * | 3.68313748630492e-06 | 0.0280466402526712 | -7.736718169049 |
|  | chr10 | 97321386 | 97321386 | * | 8.59975267153201e-07 | 0.0163972973909036 | -8.52150871527346 |
|  | chr10 | 98134622 | 98134622 | * | 8.01995185115009e-06 | 0.0366425698605044 | -2.87610619469027 |
|  | chr10 | 98409804 | 98409804 | * | 1.56838474830324e-06 | 0.0208624867957783 | 6.17247343711391 |
|  | chr10 | 99589064 | 99589064 | * | 2.99060741466154e-07 | 0.0108087291991534 | -7.31458265933355 |
|  | chr10 | 103622397 | 103622397 | * | 1.36865397835551e-05 | 0.0444864166021639 | 3.70163158822922 |
|  | chr10 | 110359977 | 110359977 | * | 1.07589665289533e-05 | 0.0405187716477507 | -3.23840631485829 |
|  | chr10 | 110490015 | 110490015 | * | 6.08070746353667e-06 | 0.033300446843227 | 3.58851674641149 |
|  | chr10 | 117344667 | 117344667 | * | 1.58523577456708e-06 | 0.0208624867957783 | 3.55374461979914 |
|  | chr10 | 123559042 | 123559042 | * | 1.74103452561239e-05 | 0.0485151792386732 | 13.426652712367 |
|  | chr10 | 125867474 | 125867474 | * | 1.70272440535849e-05 | 0.0482254425991749 | 5.16610984635701 |
|  | chr10 | 129555313 | 129555313 | * | 4.57505443303526e-06 | 0.0311197644277593 | -7.0507842832738 |
|  | chr10 | 129808288 | 129808288 | * | 1.43549614870554e-05 | 0.0451834658383908 | 4.97170548497682 |
|  | chr10 | 130368570 | 130368570 | * | 3.58087512744474e-07 | 0.0117501795376771 | 5.8129839189994 |
|  | chr10 | 132363646 | 132363646 | * | 1.24411856662424e-05 | 0.0425789195721407 | 4.31065467036691 |
|  | chr10 | 132424211 | 132424211 | * | 3.80510765170283e-06 | 0.0280920309052914 | 8.68702081051478 |
|  | chr10 | 132785115 | 132785115 | * | 7.07454787450018e-06 | 0.0353951177460461 | 10.8600125549278 |
|  | chr10 | 133031006 | 133031006 | * | 1.75498927109273e-05 | 0.0487357904199364 | -16.8619705946895 |
|  | chr11 | 476325 | 476325 | * | 1.2846173665326e-05 | 0.043325080448427 | 3.02267002518891 |
|  | chr11 | 615246 | 615246 | * | 3.08176330115638e-09 | 0.00186564634210313 | 25.1050420168067 |
|  | chr11 | 691425 | 691425 | * | 5.51383325804678e-06 | 0.0322510100560176 | 7.83672266430888 |
|  | chr11 | 762207 | 762207 | * | 7.52644886771968e-06 | 0.0360517060018553 | -3.87144432652891 |
|  | chr11 | 1195586 | 1195586 | * | 6.4226952971394e-06 | 0.034035603327331 | -6.00858369098712 |
|  | chr11 | 4394016 | 4394016 | * | 4.84073153589861e-06 | 0.0313575170167993 | -14.4972735736135 |
|  | chr11 | 8243013 | 8243013 | * | 4.97771101448717e-06 | 0.0313575170167993 | 12.7194267389841 |
|  | chr11 | 18388161 | 18388161 | * | 5.97998956722758e-06 | 0.0331366821892864 | -2.68595041322314 |
|  | chr11 | 32333592 | 32333592 | * | 9.40743011104015e-06 | 0.039029663844326 | -3.50467289719626 |
|  | chr11 | 33829171 | 33829171 | * | 2.61051604952553e-06 | 0.0258271394599251 | -3.17124735729387 |
|  | chr11 | 35525812 | 35525812 | * | 6.19007840144583e-07 | 0.0145628956625912 | 7.28722610450366 |
|  | chr11 | 35928493 | 35928493 | * | 7.3649699344453e-06 | 0.0357736833443673 | -6.94726585315125 |
|  | chr11 | 36397725 | 36397725 | * | 3.63357952836963e-06 | 0.0278443843952925 | 7.09422078474764 |
|  | chr11 | 41716381 | 41716381 | * | 6.31601379405131e-06 | 0.033987607108273 | -6.23809185499028 |
|  | chr11 | 44250702 | 44250702 | * | 2.51611257511453e-06 | 0.0256001873955027 | -5.39811066126855 |
|  | chr11 | 47329523 | 47329523 | * | 9.7448308364234e-06 | 0.0397414035646381 | 4.42163066525312 |
|  | chr11 | 50170761 | 50170761 | * | 1.38866136808623e-05 | 0.0448358206843097 | 4.24242424242425 |
|  | chr11 | 50317371 | 50317371 | * | 1.07153933937878e-06 | 0.0182729980846848 | 14.5027214250371 |
|  | chr11 | 57182186 | 57182186 | * | 2.13241963906402e-06 | 0.023273252720545 | -3.81861575178998 |
|  | chr11 | 61147486 | 61147486 | * | 1.72620262325092e-05 | 0.048365839378543 | -14.8665832494074 |
|  | chr11 | 61294098 | 61294098 | * | 7.30622173308442e-08 | 0.00632913995781165 | 8.48481779668169 |
|  | chr11 | 62773435 | 62773435 | * | 1.78361211844962e-05 | 0.0491802621659287 | -2.22672064777328 |
|  | chr11 | 62775805 | 62775805 | * | 8.4242919461169e-07 | 0.0163328485347103 | -2.868068833652 |
|  | chr11 | 64074051 | 64074051 | * | 1.48326330256204e-06 | 0.0201784722939121 | 8.68598429574039 |
|  | chr11 | 65087073 | 65087073 | * | 1.52858587875713e-09 | 0.00123383936385754 | 4.88353567088412 |
|  | chr11 | 66400134 | 66400134 | * | 9.91901440763186e-06 | 0.0398990047799455 | -10.9401919763721 |
|  | chr11 | 66596581 | 66596581 | * | 4.69116531976059e-06 | 0.0313575170167993 | 4.50109186393901 |
|  | chr11 | 67400553 | 67400553 | * | 3.15036204774438e-06 | 0.0267673661748649 | 16.9434598483943 |
|  | chr11 | 73204829 | 73204829 | * | 1.24247844532101e-06 | 0.0190538219820252 | -9.02326582596373 |
|  | chr11 | 76596284 | 76596284 | * | 6.2660512942237e-08 | 0.00632913995781165 | -5.82601839317861 |
|  | chr11 | 85305606 | 85305606 | * | 3.52959132861299e-06 | 0.0277638687256849 | -3.94707628307002 |
|  | chr11 | 85630921 | 85630921 | * | 2.77576198227311e-08 | 0.00475876970870058 | -4.15752313710525 |
|  | chr11 | 102217782 | 102217782 | * | 5.87737662209993e-06 | 0.0328689369263055 | -6.41025641025641 |
|  | chr11 | 117139203 | 117139203 | * | 7.80044584695089e-06 | 0.0364983966694477 | -19.4035947712418 |
|  | chr11 | 117191043 | 117191043 | * | 8.27953151716095e-07 | 0.0163328485347103 | -4.75198976050765 |
|  | chr11 | 122949724 | 122949724 | * | 1.13521713775322e-05 | 0.0410799299966808 | 2.7027027027027 |
|  | chr11 | 125625472 | 125625472 | * | 8.62987272008938e-06 | 0.037585439420415 | -3.03030303030303 |
|  | chr11 | 126303961 | 126303961 | * | 1.28288573425337e-06 | 0.0190538219820252 | -3.16831683168317 |
|  | chr11 | 127000441 | 127000441 | * | 4.15207469561601e-06 | 0.0296589309432146 | 6.56837422872722 |
|  | chr11 | 129375888 | 129375888 | * | 1.2266890319611e-06 | 0.0190538219820252 | -3.65591397849462 |
|  | chr11 | 132746762 | 132746762 | * | 8.74278644072516e-06 | 0.0379407318863721 | -7.88712359312976 |
|  | chr12 | 1500511 | 1500511 | * | 4.36281161082836e-06 | 0.0303582860999448 | -5.76657709347084 |
|  | chr12 | 2959565 | 2959565 | * | 1.33965738565331e-05 | 0.0440217640066932 | -2.60876051242844 |
|  | chr12 | 7697103 | 7697103 | * | 4.72963857264528e-06 | 0.0313575170167993 | 4.3859649122807 |
|  | chr12 | 9162258 | 9162258 | * | 2.29862335995348e-06 | 0.0244131040502259 | 5.23910236014149 |
|  | chr12 | 9448651 | 9448651 | * | 3.62189819469872e-06 | 0.0278429801819269 | 3.04878048780488 |
|  | chr12 | 12295685 | 12295685 | * | 1.02441114518976e-05 | 0.0402474126567004 | 3.59897172236504 |
|  | chr12 | 15420019 | 15420019 | * | 3.61396102749096e-06 | 0.0278429801819269 | -3.80100847806861 |
|  | chr12 | 32547809 | 32547809 | * | 3.46720864136509e-07 | 0.0117501795376771 | 5.33707865168539 |
|  | chr12 | 42311663 | 42311663 | * | 1.26793468037919e-06 | 0.0190538219820252 | -4.37710437710438 |
|  | chr12 | 47405578 | 47405578 | * | 1.50350249139648e-05 | 0.0463189237354633 | -3.68098159509203 |
|  | chr12 | 47878049 | 47878049 | * | 5.06512046754518e-06 | 0.0313575170167993 | -7.16525634644102 |
|  | chr12 | 51270378 | 51270378 | * | 1.48894187043776e-07 | 0.00816944669016107 | 4.15647921760391 |
|  | chr12 | 51294343 | 51294343 | * | 6.94404156200661e-06 | 0.0349588605149427 | 3.51610801978449 |
|  | chr12 | 52139249 | 52139249 | * | 7.9194289854272e-06 | 0.0366425698605044 | 4.00876828516985 |
|  | chr12 | 52139271 | 52139271 | * | 1.26395208809273e-05 | 0.0429270569962312 | -7.98733286418015 |
|  | chr12 | 54031303 | 54031303 | * | 1.22710360886739e-05 | 0.0424495632726324 | -3.40909090909091 |
|  | chr12 | 54326431 | 54326431 | * | 2.74346724438192e-06 | 0.0261031234884416 | -4.77243702456088 |
|  | chr12 | 56118480 | 56118480 | * | 4.21865815670931e-06 | 0.0299578049996318 | 5.0251256281407 |
|  | chr12 | 56266470 | 56266470 | * | 4.7751032184754e-06 | 0.0313575170167993 | 17.2726837213759 |
|  | chr12 | 62639876 | 62639876 | * | 1.3224517487757e-05 | 0.0437480588205542 | 3.05297419689609 |
|  | chr12 | 71839786 | 71839786 | * | 1.07835466337509e-05 | 0.0405187716477507 | 3.95560747663551 |
|  | chr12 | 79569233 | 79569233 | * | 4.78774363272224e-06 | 0.0313575170167993 | -6.15191959136596 |
|  | chr12 | 81473334 | 81473334 | * | 1.66013819944036e-05 | 0.0479530277492557 | -9.50606486938667 |
|  | chr12 | 83325588 | 83325588 | * | 9.32209366333506e-06 | 0.0389962445826831 | 10.1619433198381 |
|  | chr12 | 89342672 | 89342672 | * | 1.82988166547853e-05 | 0.0496205507199091 | 2.62626262626262 |
|  | chr12 | 89353851 | 89353851 | * | 5.6383663101015e-07 | 0.0139321212339453 | 8.32478497593309 |
|  | chr12 | 89388966 | 89388966 | * | 2.40370345333022e-06 | 0.0250744346058153 | 4.06305923828052 |
|  | chr12 | 93467708 | 93467708 | * | 1.71904958506644e-05 | 0.048365839378543 | 6.74083117400837 |
|  | chr12 | 98511582 | 98511582 | * | 9.85776960152088e-06 | 0.0397414035646381 | 3.50877192982456 |
|  | chr12 | 101560657 | 101560657 | * | 1.07874114302932e-05 | 0.0405187716477507 | 5.83333333333333 |
|  | chr12 | 103709349 | 103709349 | * | 1.80461053162585e-05 | 0.0493776310198698 | -4.10775307327031 |
|  | chr12 | 108768794 | 108768794 | * | 1.7163564203347e-05 | 0.048365839378543 | 3.17613531899246 |
|  | chr12 | 111586478 | 111586478 | * | 4.93254729836073e-06 | 0.0313575170167993 | 5.12820512820513 |
|  | chr12 | 111778609 | 111778609 | * | 6.8769707901183e-06 | 0.0349115261097819 | 7.2538860103627 |
|  | chr12 | 112194994 | 112194994 | * | 1.5937604967921e-05 | 0.0471449739477541 | 3.08183436675057 |
|  | chr12 | 113154691 | 113154691 | * | 1.59841228560585e-05 | 0.0471449739477541 | -5.93607305936073 |
|  | chr12 | 113332722 | 113332722 | * | 4.96778252934935e-06 | 0.0313575170167993 | -10.907922614706 |
|  | chr12 | 113468408 | 113468408 | * | 1.14218369691789e-05 | 0.0410799299966808 | -5.71007798641095 |
|  | chr12 | 116591236 | 116591236 | * | 1.67213891812429e-05 | 0.0480106905549777 | 3.125 |
|  | chr12 | 121918886 | 121918886 | * | 6.45417938185866e-07 | 0.0147443353327657 | -17.5192185422808 |
|  | chr12 | 121968114 | 121968114 | * | 4.86373020767751e-06 | 0.0313575170167993 | -4.92424242424242 |
|  | chr12 | 122654450 | 122654450 | * | 1.0497860441749e-05 | 0.0402474126567004 | 5.21862176312439 |
|  | chr12 | 123602136 | 123602136 | * | 7.17708567933964e-06 | 0.0354684397187285 | -4.16666666666667 |
|  | chr12 | 123644443 | 123644443 | * | 1.78724248031585e-05 | 0.0491802621659287 | -3.2967032967033 |
|  | chr12 | 123894505 | 123894505 | * | 3.7483780435332e-06 | 0.0280920309052914 | 4.40962364417268 |
|  | chr12 | 124349394 | 124349394 | * | 1.76563357076743e-05 | 0.0489228654191022 | 5.64516129032258 |
|  | chr12 | 124616849 | 124616849 | * | 1.98666526452325e-06 | 0.0226622047432119 | -6.52941562151682 |
|  | chr12 | 125430330 | 125430330 | * | 1.67729030930864e-05 | 0.0480106905549777 | 8.67171080285834 |
|  | chr12 | 129513776 | 129513776 | * | 9.61779585397877e-06 | 0.0394742217153916 | -2.6369168356998 |
|  | chr12 | 131564416 | 131564416 | * | 9.28136204038408e-08 | 0.0074917019676711 | -16.6383749567483 |
|  | chr12 | 132147876 | 132147876 | * | 7.95532623381354e-06 | 0.0366425698605044 | -3.41850594227504 |
|  | chr12 | 132559808 | 132559808 | * | 6.25028579110982e-07 | 0.0145628956625912 | 10.1258968458102 |
|  | chr13 | 24357843 | 24357843 | * | 1.42610317676803e-05 | 0.0450629053763874 | 4.8698738170347 |
|  | chr13 | 24764177 | 24764177 | * | 3.11324821556774e-06 | 0.0267163091716573 | -3.35277591973244 |
|  | chr13 | 26950673 | 26950673 | * | 1.52589144353784e-07 | 0.00816944669016107 | -8.96117900790798 |
|  | chr13 | 46398786 | 46398786 | * | 3.97507838872503e-06 | 0.0290040143512226 | 4.94186046511628 |
|  | chr13 | 64443371 | 64443371 | * | 1.05793747548856e-05 | 0.0402474126567004 | 6.12730957558544 |
|  | chr13 | 80293012 | 80293012 | * | 1.22229956106226e-05 | 0.0424495632726324 | 6.2131087063453 |
|  | chr13 | 85951733 | 85951733 | * | 7.46678700809761e-07 | 0.0158605756232505 | 7.04050770985175 |
|  | chr13 | 94724649 | 94724649 | * | 6.06580800523873e-11 | 0.000146885421247337 | 12.6757633746568 |
|  | chr13 | 107865996 | 107865996 | * | 1.38683877976966e-05 | 0.0448358206843097 | 8.41654866711814 |
|  | chr13 | 107867566 | 107867566 | * | 1.28406502676499e-05 | 0.043325080448427 | 7.62553235334524 |
|  | chr13 | 109782741 | 109782741 | * | 1.91912578338941e-07 | 0.00903748936933798 | 4.38356164383562 |
|  | chr13 | 110615824 | 110615824 | * | 5.03451074954343e-06 | 0.0313575170167993 | 3.46534653465347 |
|  | chr13 | 113412155 | 113412155 | * | 3.79274284172897e-08 | 0.00574015272892175 | 6.69014084507042 |
|  | chr14 | 20683643 | 20683643 | * | 2.7342280437477e-06 | 0.0261031234884416 | 3.6745406824147 |
|  | chr14 | 34839284 | 34839284 | * | 2.83595988765535e-06 | 0.0261031234884416 | -11.4115855444269 |
|  | chr14 | 38256040 | 38256040 | * | 1.64774545415055e-05 | 0.0478999603521566 | -4.12442092753763 |
|  | chr14 | 52069500 | 52069500 | * | 1.99847508405513e-06 | 0.0226622047432119 | 3.82165605095541 |
|  | chr14 | 57914387 | 57914387 | * | 1.06168915650188e-07 | 0.0080341037651036 | 13.1137448052948 |
|  | chr14 | 64655787 | 64655787 | * | 1.50922589056759e-06 | 0.02041696804476 | 3.17460317460317 |
|  | chr14 | 65362689 | 65362689 | * | 1.43727441828781e-07 | 0.00816944669016107 | -5.71763126704652 |
|  | chr14 | 65411702 | 65411702 | * | 5.25901923885117e-06 | 0.0314205302796785 | -2.68595041322314 |
|  | chr14 | 74769703 | 74769703 | * | 2.69413987164203e-06 | 0.0261031234884416 | -3.51966873706004 |
|  | chr14 | 91355048 | 91355048 | * | 7.37181063915321e-06 | 0.0357736833443673 | -5.6547619047619 |
|  | chr14 | 99682798 | 99682798 | * | 1.39994353160384e-05 | 0.0448427563112155 | 8.05393773364572 |
|  | chr14 | 100170655 | 100170655 | * | 1.1434036077904e-05 | 0.0410799299966808 | 4.02476780185759 |
|  | chr14 | 102079298 | 102079298 | * | 3.37168689990897e-06 | 0.027234173286512 | -4.65557259101634 |
|  | chr14 | 102516811 | 102516811 | * | 5.69727461600943e-06 | 0.0327072575386812 | 3.96341463414634 |
|  | chr14 | 103220940 | 103220940 | * | 1.10911371942144e-05 | 0.0408887682689852 | 5.71458529936039 |
|  | chr14 | 103640453 | 103640453 | * | 8.59635589672317e-06 | 0.037585439420415 | -4.49101796407185 |
|  | chr15 | 22786057 | 22786057 | * | 1.13144778840882e-05 | 0.0410799299966808 | 4.38871473354232 |
|  | chr15 | 23068504 | 23068504 | * | 4.95170515109122e-07 | 0.0137075792844887 | -5.57577718460999 |
|  | chr15 | 27369544 | 27369544 | * | 9.37400296137303e-06 | 0.0390024721049083 | -6.31672137258354 |
|  | chr15 | 28102840 | 28102840 | * | 5.43174007383278e-07 | 0.0139321212339453 | 6.23452721013698 |
|  | chr15 | 29821653 | 29821653 | * | 2.42056077141682e-06 | 0.0250744346058153 | 4.33734939759036 |
|  | chr15 | 30618402 | 30618402 | * | 1.58437490368709e-06 | 0.0208624867957783 | -3.27868852459017 |
|  | chr15 | 31440710 | 31440710 | * | 1.05009854355458e-05 | 0.0402474126567004 | -11.2688690395786 |
|  | chr15 | 34754934 | 34754934 | * | 2.2066808456757e-06 | 0.0239620900220176 | 10.9527603198489 |
|  | chr15 | 40928146 | 40928146 | * | 6.91638552896632e-06 | 0.0349244619030497 | 8.36799608514803 |
|  | chr15 | 42245751 | 42245751 | * | 2.85659267811853e-06 | 0.0261031234884416 | -6.55593012565124 |
|  | chr15 | 43163557 | 43163557 | * | 1.44845900781228e-05 | 0.0452579147051184 | -3.19530466229733 |
|  | chr15 | 43502897 | 43502897 | * | 4.78082123042845e-06 | 0.0313575170167993 | 3.98936170212766 |
|  | chr15 | 43513156 | 43513156 | * | 3.98962080108987e-06 | 0.0290040143512226 | -3.06603773584906 |
|  | chr15 | 46924134 | 46924134 | * | 4.38520046481812e-06 | 0.0304266443173968 | 6.90903398751639 |
|  | chr15 | 49160675 | 49160675 | * | 1.63927227105222e-06 | 0.021114620328688 | 4.84330484330484 |
|  | chr15 | 49732403 | 49732403 | * | 2.82661696608139e-06 | 0.0261031234884416 | -8.48753348444262 |
|  | chr15 | 50766994 | 50766994 | * | 3.0271042331711e-06 | 0.0262732141249285 | 2.66826614535509 |
|  | chr15 | 59131479 | 59131479 | * | 3.98498805239094e-06 | 0.0290040143512226 | -5.01672240802675 |
|  | chr15 | 63966906 | 63966906 | * | 1.58845475699479e-05 | 0.0471449739477541 | 2.87520111054602 |
|  | chr15 | 64022587 | 64022587 | * | 1.72955507868895e-05 | 0.048365839378543 | 4.63022165387895 |
|  | chr15 | 64567897 | 64567897 | * | 5.01884693864211e-06 | 0.0313575170167993 | 4.14509894273354 |
|  | chr15 | 75186032 | 75186032 | * | 1.82608066662463e-06 | 0.0221112105859451 | -7.17295202939275 |
|  | chr15 | 76161994 | 76161994 | * | 5.18836974587012e-06 | 0.0313575170167993 | -12.0170544267818 |
|  | chr15 | 79923899 | 79923899 | * | 2.84357269984889e-06 | 0.0261031234884416 | 12.0500201194787 |
|  | chr15 | 82319662 | 82319662 | * | 2.13363450806989e-06 | 0.023273252720545 | -6.09318996415771 |
|  | chr15 | 84726515 | 84726515 | * | 1.75632028232892e-06 | 0.0218101744081448 | -3.43203485068502 |
|  | chr15 | 89427042 | 89427042 | * | 9.00262115636795e-06 | 0.0384844115216733 | 4.0882100697574 |
|  | chr15 | 90529458 | 90529458 | * | 1.50624684927689e-05 | 0.0463189237354633 | -5.8387041773231 |
|  | chr15 | 95811938 | 95811938 | * | 1.36125087925381e-06 | 0.0190538219820252 | 6.5806687331314 |
|  | chr15 | 96341281 | 96341281 | * | 4.14681454024622e-08 | 0.0058666195414222 | 14.1258805128381 |
|  | chr15 | 96354640 | 96354640 | * | 7.72303044561775e-07 | 0.015984237297442 | -4.53333333333333 |
|  | chr15 | 99341292 | 99341292 | * | 1.0133789347943e-05 | 0.0402474126567004 | -5.31586216923524 |
|  | chr15 | 101446031 | 101446031 | * | 1.23654640459437e-08 | 0.0029090210526172 | -9.69135802469135 |
|  | chr16 | 73918 | 73918 | * | 2.90213319093055e-07 | 0.0107812291291298 | -6.51340996168582 |
|  | chr16 | 323167 | 323167 | * | 1.59718167991118e-05 | 0.0471449739477541 | 2.89855072463768 |
|  | chr16 | 484438 | 484438 | * | 3.72196473019056e-06 | 0.0280920309052914 | 3.8135593220339 |
|  | chr16 | 1009760 | 1009760 | * | 8.59761380543363e-06 | 0.037585439420415 | -8.9301941346551 |
|  | chr16 | 1498961 | 1498961 | * | 1.18396434833447e-06 | 0.0190538219820252 | 6.62880399879293 |
|  | chr16 | 1551278 | 1551278 | * | 4.85632178370615e-10 | 0.000587986687260987 | -14.4001801429885 |
|  | chr16 | 1778032 | 1778032 | * | 7.95187107327392e-06 | 0.0366425698605044 | -8.35394385026738 |
|  | chr16 | 2179276 | 2179276 | * | 1.5568173729178e-06 | 0.0208280747506023 | -19.2307692307692 |
|  | chr16 | 2183038 | 2183038 | * | 1.25865192202877e-06 | 0.0190538219820252 | 7.05237862495927 |
|  | chr16 | 2204079 | 2204079 | * | 4.84168299081351e-06 | 0.0313575170167993 | 4.82002851033499 |
|  | chr16 | 3297760 | 3297760 | * | 1.78079382174827e-05 | 0.0491802621659287 | 2.80876078149886 |
|  | chr16 | 3471460 | 3471460 | * | 6.89708258635913e-06 | 0.0349244619030497 | 3.97553516819572 |
|  | chr16 | 4253684 | 4253684 | * | 1.13717200199963e-05 | 0.0410799299966808 | 2.4115539632781 |
|  | chr16 | 4475767 | 4475767 | * | 8.3778068717432e-06 | 0.0371558956995223 | -7.4050867910517 |
|  | chr16 | 5999643 | 5999643 | * | 1.11125461198374e-05 | 0.0408887682689852 | -8.38091710649506 |
|  | chr16 | 9763682 | 9763682 | * | 1.4727390053847e-05 | 0.0458391151214425 | 7.23034353668833 |
|  | chr16 | 12001258 | 12001258 | * | 1.6002912118446e-05 | 0.0471449739477541 | 4.50286756143325 |
|  | chr16 | 14349619 | 14349619 | * | 1.41221104144254e-05 | 0.0450555048141686 | -2.8943002960576 |
|  | chr16 | 14631533 | 14631533 | * | 2.78969757938723e-06 | 0.0261031234884416 | 4.50704225352113 |
|  | chr16 | 15045253 | 15045253 | * | 1.34625911193208e-06 | 0.0190538219820252 | -5.03076407608778 |
|  | chr16 | 19115003 | 19115003 | * | 1.66806796140381e-05 | 0.0479724261121869 | 3.42465753424658 |
|  | chr16 | 27219292 | 27219292 | * | 2.77938358704272e-07 | 0.0106831166935161 | -6.07347911540307 |
|  | chr16 | 27566337 | 27566337 | * | 7.84852895062589e-07 | 0.0161063187782526 | 6.1400641787497 |
|  | chr16 | 28984850 | 28984850 | * | 1.31484411560659e-05 | 0.0437061148818485 | 9.25 |
|  | chr16 | 29660207 | 29660207 | * | 6.86216803929427e-06 | 0.0349100275189403 | 4.80960566755222 |
|  | chr16 | 30492531 | 30492531 | * | 1.30655502656108e-06 | 0.0190538219820252 | -4.82897384305835 |
|  | chr16 | 31148504 | 31148504 | * | 1.5708830497286e-08 | 0.00315974099486277 | -17.2798755560614 |
|  | chr16 | 32885373 | 32885373 | * | 7.29888615826206e-06 | 0.0357736833443673 | 11.5406162464986 |
|  | chr16 | 49543785 | 49543785 | * | 1.39813593906158e-05 | 0.0448427563112155 | 5.47606239913931 |
|  | chr16 | 58496173 | 58496173 | * | 1.0792596796324e-05 | 0.0405187716477507 | 15.7114624505929 |
|  | chr16 | 66948713 | 66948713 | * | 1.68487389880585e-06 | 0.021423060768662 | -4.12844036697248 |
|  | chr16 | 67164831 | 67164831 | * | 1.45915944302152e-07 | 0.00816944669016107 | -4.07673860911271 |
|  | chr16 | 67165958 | 67165958 | * | 3.09302465216986e-06 | 0.0266542885373435 | 7.04621647509579 |
|  | chr16 | 67399452 | 67399452 | * | 1.65145482901581e-05 | 0.0479246852482212 | -13.3085857307573 |
|  | chr16 | 68388177 | 68388177 | * | 4.68562630686897e-06 | 0.0313575170167993 | 12.8258726302009 |
|  | chr16 | 69098112 | 69098112 | * | 3.56932261174349e-06 | 0.0277638687256849 | -6.86659682207089 |
|  | chr16 | 70338516 | 70338516 | * | 1.36661326514931e-05 | 0.0444797901420737 | -3.84312007011394 |
|  | chr16 | 73047804 | 73047804 | * | 9.82458821303416e-06 | 0.0397414035646381 | 8.52780456444873 |
|  | chr16 | 73258475 | 73258475 | * | 8.91381312522755e-06 | 0.0384076064251697 | -5.75800980022616 |
|  | chr16 | 74615261 | 74615261 | * | 2.60285391360818e-06 | 0.0258271394599251 | 3.4729907773386 |
|  | chr16 | 77803670 | 77803670 | * | 5.5109929700122e-06 | 0.0322510100560176 | 4.61538461538461 |
|  | chr16 | 81651345 | 81651345 | * | 2.48548829267391e-07 | 0.0104484958144424 | 6.59340659340659 |
|  | chr16 | 81802348 | 81802348 | * | 2.09580924262947e-06 | 0.023273252720545 | 4.21836228287841 |
|  | chr16 | 85303678 | 85303678 | * | 1.98680833119907e-06 | 0.0226622047432119 | -7.28013552589304 |
|  | chr16 | 87419491 | 87419491 | * | 1.02499945460069e-05 | 0.0402474126567004 | 4.45654500059446 |
|  | chr16 | 88903155 | 88903155 | * | 3.24804264523886e-07 | 0.011566523462894 | 8.05964745046314 |
|  | chr16 | 89160295 | 89160295 | * | 5.08978439531009e-06 | 0.0313575170167993 | 5.88235294117647 |
|  | chr16 | 89179018 | 89179018 | * | 1.56350889048357e-05 | 0.0469371566071801 | -2.88248337028825 |
|  | chr16 | 89246907 | 89246907 | * | 1.86721958875754e-06 | 0.0223838124652655 | 19.9421250854309 |
|  | chr17 | 936515 | 936515 | * | 3.70036445061261e-06 | 0.0280894897443775 | 3.94731822285901 |
|  | chr17 | 1182657 | 1182657 | * | 9.61036535711878e-06 | 0.0394742217153916 | -4.49219309554505 |
|  | chr17 | 3901289 | 3901289 | * | 1.188227465169e-05 | 0.0418825275394198 | 10.2565275943674 |
|  | chr17 | 4501609 | 4501609 | * | 5.1828677815594e-06 | 0.0313575170167993 | 10.794740764399 |
|  | chr17 | 4596987 | 4596987 | * | 1.5414094966607e-05 | 0.046774071176169 | 5.102770596956 |
|  | chr17 | 8765040 | 8765040 | * | 1.3144049335684e-05 | 0.0437061148818485 | -3.59477124183006 |
|  | chr17 | 10609726 | 10609726 | * | 1.11444317902724e-05 | 0.0408887682689852 | 6.14531186578271 |
|  | chr17 | 16417819 | 16417819 | * | 1.42919133924222e-05 | 0.0450629053763874 | 3.35418010488824 |
|  | chr17 | 16666752 | 16666752 | * | 7.99795835237274e-06 | 0.0366425698605044 | -12.3298533104269 |
|  | chr17 | 17518537 | 17518537 | * | 1.69423379447805e-05 | 0.0482096316636455 | 3.46676260002808 |
|  | chr17 | 18154099 | 18154099 | * | 1.20324875940849e-05 | 0.0421055516129941 | -3.97111139685397 |
|  | chr17 | 21755475 | 21755475 | * | 1.70351068560861e-05 | 0.0482254425991749 | 4.15224913494809 |
|  | chr17 | 28227362 | 28227362 | * | 6.86225908279332e-06 | 0.0349100275189403 | 6.98004444346659 |
|  | chr17 | 31409827 | 31409827 | * | 9.86342257949516e-06 | 0.0397414035646381 | 3.52492148466427 |
|  | chr17 | 32132724 | 32132724 | * | 7.46397745236181e-07 | 0.0158605756232505 | 5.09208147815643 |
|  | chr17 | 36661426 | 36661426 | * | 7.65145681419443e-07 | 0.0159726205782182 | 9.3528691621449 |
|  | chr17 | 37487644 | 37487644 | * | 4.55987792883869e-06 | 0.0311197644277593 | 6.39786263645059 |
|  | chr17 | 37844916 | 37844916 | * | 2.28476788042397e-06 | 0.0244131040502259 | 4.56803455723542 |
|  | chr17 | 39126990 | 39126990 | * | 2.62374353544992e-06 | 0.0258271394599251 | -4.66893039049235 |
|  | chr17 | 39981127 | 39981127 | * | 1.25267652365513e-05 | 0.0427841330747974 | 3.00546448087432 |
|  | chr17 | 40287899 | 40287899 | * | 1.56174906367517e-05 | 0.0469371566071801 | 3.35195530726257 |
|  | chr17 | 42085892 | 42085892 | * | 1.52137989765452e-05 | 0.046434456775118 | -3.82513661202186 |
|  | chr17 | 42675746 | 42675746 | * | 3.60098350354462e-06 | 0.0278429801819269 | 3.69393139841689 |
|  | chr17 | 43713974 | 43713974 | * | 1.15045861049031e-05 | 0.0412111122709942 | 8.9213441568954 |
|  | chr17 | 44004889 | 44004889 | * | 9.41277385467556e-06 | 0.039029663844326 | 3.50411046009901 |
|  | chr17 | 44241955 | 44241955 | * | 5.2748432220422e-06 | 0.0314205302796785 | 4.33436532507739 |
|  | chr17 | 45469902 | 45469902 | * | 7.20279790930761e-06 | 0.0355230110470949 | 4.33526011560693 |
|  | chr17 | 48107848 | 48107848 | * | 6.0730939230357e-06 | 0.033300446843227 | -3.45679012345679 |
|  | chr17 | 48554778 | 48554778 | * | 7.84939763179239e-06 | 0.0365550330541403 | 3.38868658023721 |
|  | chr17 | 50560643 | 50560643 | * | 1.41547275764989e-05 | 0.0450629053763874 | 8.99155049985813 |
|  | chr17 | 58308453 | 58308453 | * | 2.00239063962822e-06 | 0.0226622047432119 | -16.21519709755 |
|  | chr17 | 60140019 | 60140019 | * | 1.51128600849828e-07 | 0.00816944669016107 | -4.21940928270042 |
|  | chr17 | 62886495 | 62886495 | * | 6.48311227849935e-06 | 0.034128385562754 | 5.53584366654936 |
|  | chr17 | 63596174 | 63596174 | * | 1.13295284708133e-05 | 0.0410799299966808 | 2.02360876897133 |
|  | chr17 | 64785457 | 64785457 | * | 1.05422046110732e-05 | 0.0402474126567004 | 4.16831742007052 |
|  | chr17 | 76347215 | 76347215 | * | 2.9811241675423e-06 | 0.0262732141249285 | -3.22940783042651 |
|  | chr17 | 78475612 | 78475612 | * | 1.41032972164476e-05 | 0.0450555048141686 | 1.76100628930818 |
|  | chr17 | 79214029 | 79214029 | * | 5.32124236144897e-06 | 0.0315050203832809 | -3.71352785145889 |
|  | chr17 | 79903565 | 79903565 | * | 6.80971064966432e-06 | 0.034862421647341 | 4.45026178010471 |
|  | chr17 | 80665834 | 80665834 | * | 6.15488621122e-06 | 0.0334175958787932 | -6.21001041045256 |
|  | chr17 | 81204867 | 81204867 | * | 4.90417915157036e-06 | 0.0313575170167993 | 4.07523510971787 |
|  | chr17 | 81834335 | 81834335 | * | 6.10908628925676e-06 | 0.033300446843227 | 10.0283194723258 |
|  | chr17 | 81906193 | 81906193 | * | 4.79128840635636e-06 | 0.0313575170167993 | -2.99132760013189 |
|  | chr17 | 82242926 | 82242926 | * | 8.0164607371268e-06 | 0.0366425698605044 | -16.3322591587517 |
|  | chr17 | 82663760 | 82663760 | * | 6.38111732643071e-06 | 0.034035603327331 | 4.04312668463612 |
|  | chr18 | 3452445 | 3452445 | * | 5.47086568861524e-06 | 0.0322332624375137 | -3.36498822143698 |
|  | chr18 | 3743544 | 3743544 | * | 1.04033569771367e-05 | 0.0402474126567004 | -5.10067243591599 |
|  | chr18 | 5629486 | 5629486 | * | 1.92587025520217e-07 | 0.00903748936933798 | -3.17725752508361 |
|  | chr18 | 7279870 | 7279870 | * | 2.40509483413734e-06 | 0.0250744346058153 | 2.85171102661597 |
|  | chr18 | 8959689 | 8959689 | * | 1.31757404153609e-05 | 0.0437061148818485 | -7.47317073170731 |
|  | chr18 | 9836141 | 9836141 | * | 1.22442724356282e-05 | 0.0424495632726324 | -5.45492270982467 |
|  | chr18 | 32927969 | 32927969 | * | 1.55088867919586e-05 | 0.0468854558579503 | 4.0455330507144 |
|  | chr18 | 37317115 | 37317115 | * | 7.165337073076e-06 | 0.0354684397187285 | -4.83870967741935 |
|  | chr18 | 37567215 | 37567215 | * | 5.19196958098639e-07 | 0.0139321212339453 | -4.74576271186441 |
|  | chr18 | 58691682 | 58691682 | * | 1.64761924135674e-05 | 0.0478999603521566 | -14.7362954590582 |
|  | chr18 | 64756372 | 64756372 | * | 8.16170429176444e-06 | 0.0367265471261412 | -4.1166077738516 |
|  | chr18 | 75090597 | 75090597 | * | 1.46313095956927e-07 | 0.00816944669016107 | -7.10160427807487 |
|  | chr18 | 76491561 | 76491561 | * | 1.42674412350095e-05 | 0.0450629053763874 | 2.97872340425532 |
|  | chr18 | 79005927 | 79005927 | * | 3.92748447805114e-07 | 0.0121929813020765 | 8.03251053582179 |
|  | chr19 | 420780 | 420780 | * | 1.12106006918777e-05 | 0.04100727659215 | -4.36363636363636 |
|  | chr19 | 785070 | 785070 | * | 1.00542402830628e-05 | 0.0402424041766698 | -9.38108213312574 |
|  | chr19 | 919923 | 919923 | * | 1.2616496874556e-05 | 0.0429270569962312 | 8.97080267961889 |
|  | chr19 | 1513082 | 1513082 | * | 1.17170240655591e-05 | 0.0416028401796149 | 3.23325635103926 |
|  | chr19 | 1595553 | 1595553 | * | 1.55403460770476e-05 | 0.0469219822647122 | 3.51122349392246 |
|  | chr19 | 1757801 | 1757801 | * | 1.17149005579792e-06 | 0.0190538219820252 | 10.4107787198493 |
|  | chr19 | 1976177 | 1976177 | * | 1.49436419396493e-05 | 0.0462742867132491 | -5.44270302123705 |
|  | chr19 | 2096033 | 2096033 | * | 1.44234449582004e-05 | 0.0451834658383908 | -2.66393442622951 |
|  | chr19 | 2124148 | 2124148 | * | 3.82472064962926e-07 | 0.0120281553498927 | -8.04620841721792 |
|  | chr19 | 3032742 | 3032742 | * | 8.87902257227625e-06 | 0.0383258973412953 | -2.99074920638526 |
|  | chr19 | 4176461 | 4176461 | * | 8.65938682702716e-06 | 0.0376462722489011 | 3.14606741573034 |
|  | chr19 | 4408414 | 4408414 | * | 1.17525996082248e-05 | 0.0416680589778977 | -3.27535280603872 |
|  | chr19 | 4924351 | 4924351 | * | 3.00422706802322e-06 | 0.0262732141249285 | 7.65590326525067 |
|  | chr19 | 5243964 | 5243964 | * | 4.81371782784968e-08 | 0.00613503523441613 | 6.16883116883117 |
|  | chr19 | 5818002 | 5818002 | * | 3.59952835544408e-07 | 0.0117501795376771 | 5.72289156626506 |
|  | chr19 | 6805146 | 6805146 | * | 8.8214408728719e-06 | 0.0381453438184399 | 3.69458128078818 |
|  | chr19 | 7620821 | 7620821 | * | 5.91281805614754e-06 | 0.0329223099813284 | 2.81954887218046 |
|  | chr19 | 7846168 | 7846168 | * | 2.65517615838173e-07 | 0.0105403137343972 | -4.86153331712063 |
|  | chr19 | 8319420 | 8319420 | * | 1.47196591418621e-06 | 0.0201784722939121 | -6.53611508623936 |
|  | chr19 | 10284042 | 10284042 | * | 6.9227863337136e-06 | 0.0349244619030497 | -5.07971820541342 |
|  | chr19 | 10834669 | 10834669 | * | 5.6424607531736e-08 | 0.00632913995781165 | -7.72494777394258 |
|  | chr19 | 10964607 | 10964607 | * | 3.56914306308506e-06 | 0.0277638687256849 | 6.17034038086669 |
|  | chr19 | 11988053 | 11988053 | * | 1.09416469944656e-05 | 0.0408251731712714 | 7.80754597336715 |
|  | chr19 | 12632862 | 12632862 | * | 1.5933746494898e-05 | 0.0471449739477541 | -4.18006430868167 |
|  | chr19 | 12696268 | 12696268 | * | 6.45707560558156e-06 | 0.0341068911473657 | 3.53260869565217 |
|  | chr19 | 12876353 | 12876353 | * | 3.18769964739122e-06 | 0.0268166249280202 | -4.97021467910531 |
|  | chr19 | 12918675 | 12918675 | * | 6.76087384896742e-06 | 0.0347593749731718 | -10.7927234099057 |
|  | chr19 | 12961376 | 12961376 | * | 1.8461587110248e-05 | 0.0499982580458389 | -3.23597175771089 |
|  | chr19 | 13750351 | 13750351 | * | 8.56414004309151e-06 | 0.037585439420415 | -4.33673469387755 |
|  | chr19 | 14491896 | 14491896 | * | 6.31461684600393e-07 | 0.0145628956625912 | 8.68707900746513 |
|  | chr19 | 17391280 | 17391280 | * | 3.00087620404307e-06 | 0.0262732141249285 | -3.17848410757946 |
|  | chr19 | 17511841 | 17511841 | * | 1.84794004086777e-05 | 0.0499982580458389 | -3.66666666666667 |
|  | chr19 | 19164541 | 19164541 | * | 1.10847098222657e-05 | 0.0408887682689852 | -3.74084562054486 |
|  | chr19 | 19359802 | 19359802 | * | 1.04609650733706e-05 | 0.0402474126567004 | -3.91282673892244 |
|  | chr19 | 29475660 | 29475660 | * | 8.2144536666094e-06 | 0.0367265471261412 | -3.70354760472503 |
|  | chr19 | 32581485 | 32581485 | * | 1.51300983470581e-05 | 0.046434456775118 | 2.10772833723653 |
|  | chr19 | 33119567 | 33119567 | * | 1.1314256267279e-05 | 0.0410799299966808 | 3.65535248041775 |
|  | chr19 | 33430032 | 33430032 | * | 1.23432072353255e-05 | 0.0425789195721407 | 6.22120086514952 |
|  | chr19 | 34559174 | 34559174 | * | 1.58306734959631e-05 | 0.0471449739477541 | -5.21468926553672 |
|  | chr19 | 35269781 | 35269781 | * | 1.66325336166866e-05 | 0.0479530277492557 | -4.02684563758389 |
|  | chr19 | 35787456 | 35787456 | * | 1.31693185286984e-05 | 0.0437061148818485 | -5.08077654086343 |
|  | chr19 | 35918721 | 35918721 | * | 1.2680354615985e-05 | 0.0430054226801131 | 4.50917546505782 |
|  | chr19 | 36570072 | 36570072 | * | 5.59899046728957e-07 | 0.0139321212339453 | 3.36711897306305 |
|  | chr19 | 39464184 | 39464184 | * | 4.07633518739481e-06 | 0.0292907181681524 | -8.3521484204078 |
|  | chr19 | 41865676 | 41865676 | * | 2.43524330453485e-06 | 0.025078736651872 | 4.59429153924567 |
|  | chr19 | 45812385 | 45812385 | * | 4.10099597655419e-06 | 0.0293807363553291 | -6.47224045403653 |
|  | chr19 | 46471549 | 46471549 | * | 4.47066413665828e-06 | 0.0309310051357322 | -5.25124688279302 |
|  | chr19 | 47129040 | 47129040 | * | 1.83214072859984e-06 | 0.0221112105859451 | -4.61350162554982 |
|  | chr19 | 47604981 | 47604981 | * | 7.73051297772162e-06 | 0.036456021313964 | 5.59190923006813 |
|  | chr19 | 48061904 | 48061904 | * | 5.83205753313647e-06 | 0.0328689369263055 | 15.0541640187291 |
|  | chr19 | 49512221 | 49512221 | * | 1.32144629074702e-08 | 0.0029090210526172 | 17.1450617283951 |
|  | chr19 | 49567215 | 49567215 | * | 1.88260165525309e-06 | 0.0224263216453233 | 4.71014492753623 |
|  | chr19 | 49800221 | 49800221 | * | 1.07382840750743e-05 | 0.0405187716477507 | 7.26256983240223 |
|  | chr19 | 50476947 | 50476947 | * | 1.81471111972981e-05 | 0.0495839338892745 | 8.46681096681097 |
|  | chr19 | 50929292 | 50929292 | * | 3.44983320779333e-06 | 0.0275705546452178 | 4.94253419452887 |
|  | chr19 | 52398075 | 52398075 | * | 1.2711602356552e-05 | 0.0430284231750481 | 5.2308962713587 |
|  | chr19 | 55193689 | 55193689 | * | 6.89584190218106e-08 | 0.00632913995781165 | -12.9910087025097 |
|  | chr19 | 55193694 | 55193694 | * | 1.48411208173134e-05 | 0.0461338050498971 | -10.4326548770993 |
|  | chr19 | 55330974 | 55330974 | * | 4.57126173208874e-06 | 0.0311197644277593 | 4.27165249023699 |
|  | chr19 | 55437155 | 55437155 | * | 3.56509515156776e-06 | 0.0277638687256849 | -7.99075704225353 |
|  | chr19 | 57614351 | 57614351 | * | 2.23653461760413e-06 | 0.024177847808489 | -2.56410256410256 |
|  | chr2 | 2116789 | 2116789 | * | 1.52026858424062e-05 | 0.046434456775118 | -10.6679502369668 |
|  | chr2 | 3483468 | 3483468 | * | 1.11224791668618e-05 | 0.0408887682689852 | -7.52440106477373 |
|  | chr2 | 3518926 | 3518926 | * | 1.41670884388068e-05 | 0.0450629053763874 | -2.18487394957983 |
|  | chr2 | 4929262 | 4929262 | * | 9.35640225235146e-06 | 0.0389962445826831 | 4.56792993266697 |
|  | chr2 | 10016384 | 10016384 | * | 7.06893712552139e-07 | 0.0157042664096339 | 7.22822822822823 |
|  | chr2 | 11926520 | 11926520 | * | 3.27538144155194e-06 | 0.027162457868297 | -7.02135448444173 |
|  | chr2 | 11943525 | 11943525 | * | 7.99864067098279e-06 | 0.0366425698605044 | -3.78548895899053 |
|  | chr2 | 12717923 | 12717923 | * | 6.51077316166529e-06 | 0.0341324465221042 | 8.6222754996645 |
|  | chr2 | 15952196 | 15952196 | * | 1.54426089959768e-05 | 0.0467778690068462 | 7.0005480953686 |
|  | chr2 | 23587287 | 23587287 | * | 9.7073000743135e-07 | 0.0174807954033733 | 10.8941537888661 |
|  | chr2 | 24077555 | 24077555 | * | 1.18889849817421e-06 | 0.0190538219820252 | -9.92912827781715 |
|  | chr2 | 25277170 | 25277170 | * | 3.32882284689235e-06 | 0.0272325936393854 | -7.04769517719158 |
|  | chr2 | 26045529 | 26045529 | * | 2.29722036120952e-06 | 0.0244131040502259 | 10.4753248142009 |
|  | chr2 | 27127812 | 27127812 | * | 9.34502987434426e-06 | 0.0389962445826831 | -5.41871921182266 |
|  | chr2 | 27134250 | 27134250 | * | 1.70211323120166e-05 | 0.0482254425991749 | 2.7906976744186 |
|  | chr2 | 27137735 | 27137735 | * | 1.23699498923887e-05 | 0.0425789195721407 | 3.98032801091925 |
|  | chr2 | 28072575 | 28072575 | * | 3.78792168276261e-06 | 0.0280920309052914 | -3.80952380952381 |
|  | chr2 | 30360762 | 30360762 | * | 6.29893233365606e-07 | 0.0145628956625912 | -5.99555946546018 |
|  | chr2 | 32646852 | 32646852 | * | 5.5973841388021e-06 | 0.0325041707698264 | -3.51437699680511 |
|  | chr2 | 38334476 | 38334476 | * | 1.1107137315293e-05 | 0.0408887682689852 | -4.0506329113924 |
|  | chr2 | 39449800 | 39449800 | * | 7.1750040148792e-06 | 0.0354684397187285 | -5.25284854692101 |
|  | chr2 | 44219671 | 44219671 | * | 5.24570646615724e-06 | 0.0314205302796785 | -5.2863436123348 |
|  | chr2 | 45545055 | 45545055 | * | 1.30142873542532e-05 | 0.043528315291757 | 2.31660231660231 |
|  | chr2 | 47307993 | 47307993 | * | 1.31256473585002e-06 | 0.0190538219820252 | 4.04193480425956 |
|  | chr2 | 47342487 | 47342487 | * | 4.90467670997669e-06 | 0.0313575170167993 | 4.49438202247191 |
|  | chr2 | 48038661 | 48038661 | * | 2.87674916720143e-06 | 0.0261571757711947 | 4.05405405405406 |
|  | chr2 | 54673773 | 54673773 | * | 2.69650526448291e-06 | 0.0261031234884416 | 10.5483605714873 |
|  | chr2 | 70100768 | 70100768 | * | 9.67736552915266e-07 | 0.0174807954033733 | -4.39560439560439 |
|  | chr2 | 70203337 | 70203337 | * | 1.24178018103362e-05 | 0.0425789195721407 | 4.42727083633339 |
|  | chr2 | 70321063 | 70321063 | * | 1.42768050736496e-05 | 0.0450629053763874 | -2.89855072463768 |
|  | chr2 | 71227828 | 71227828 | * | 6.42203283351377e-06 | 0.034035603327331 | -16.6173127542474 |
|  | chr2 | 74503277 | 74503277 | * | 5.20304760216931e-06 | 0.0313575170167993 | -2.6615969581749 |
|  | chr2 | 81985457 | 81985457 | * | 6.03412351464725e-06 | 0.0332843215228867 | 9.65568211459873 |
|  | chr2 | 85435738 | 85435738 | * | 7.0399979795464e-07 | 0.0157042664096339 | -5.16431924882629 |
|  | chr2 | 85923164 | 85923164 | * | 6.05737962751427e-06 | 0.033300446843227 | -4.20588760490803 |
|  | chr2 | 88284413 | 88284413 | * | 1.74226267122703e-06 | 0.0217471292191704 | 18.416181914331 |
|  | chr2 | 88349555 | 88349555 | * | 1.82250015221101e-06 | 0.0221112105859451 | -6.69456066945606 |
|  | chr2 | 99330064 | 99330064 | * | 1.25549171336402e-06 | 0.0190538219820252 | 8.89729395881346 |
|  | chr2 | 112490137 | 112490137 | * | 5.08950816974697e-08 | 0.00616220090389777 | 7.93164643920311 |
|  | chr2 | 115965976 | 115965976 | * | 9.17474973178589e-06 | 0.0387730207552552 | 6.29303834021947 |
|  | chr2 | 127428667 | 127428667 | * | 1.41654070586735e-06 | 0.0196498640125655 | 4.80225988700564 |
|  | chr2 | 128056779 | 128056779 | * | 1.33645618764737e-05 | 0.0440217640066932 | -6.77410030351208 |
|  | chr2 | 130727332 | 130727332 | * | 5.13162347712572e-06 | 0.0313575170167993 | -3.50404312668464 |
|  | chr2 | 134108663 | 134108663 | * | 1.36080305836791e-06 | 0.0190538219820252 | -8.88904103336527 |
|  | chr2 | 135556720 | 135556720 | * | 1.77248755202798e-07 | 0.00894194490489554 | 3.50194552529183 |
|  | chr2 | 141584094 | 141584094 | * | 3.35856230128782e-06 | 0.027234173286512 | 12.3173687805645 |
|  | chr2 | 145116662 | 145116662 | * | 2.5840885427365e-06 | 0.0258271394599251 | 6.15782964350549 |
|  | chr2 | 156029659 | 156029659 | * | 8.0137320561873e-06 | 0.0366425698605044 | 6.45900945392973 |
|  | chr2 | 156453802 | 156453802 | * | 4.00056203876103e-06 | 0.0290040143512226 | -3.53200883002207 |
|  | chr2 | 172558109 | 172558109 | * | 1.53129829218262e-05 | 0.0465327867483021 | -4.80018829125685 |
|  | chr2 | 174396092 | 174396092 | * | 3.30817185744686e-06 | 0.0272325936393854 | 8.63840043451694 |
|  | chr2 | 176002333 | 176002333 | * | 2.62248357026246e-06 | 0.0258271394599251 | -3.21100917431193 |
|  | chr2 | 177167117 | 177167117 | * | 1.6545333042407e-05 | 0.0479246852482212 | -2.74509803921569 |
|  | chr2 | 177836587 | 177836587 | * | 7.53335412218327e-06 | 0.0360517060018553 | -3.63128491620112 |
|  | chr2 | 189699961 | 189699961 | * | 5.86719325544795e-06 | 0.0328689369263055 | 3.64583333333334 |
|  | chr2 | 191882032 | 191882032 | * | 1.82143214515053e-05 | 0.0496136603360911 | 4.68085106382978 |
|  | chr2 | 197805276 | 197805276 | * | 9.56295312719535e-06 | 0.0393826317160723 | 5.43933054393306 |
|  | chr2 | 202146370 | 202146370 | * | 4.9814228148845e-07 | 0.0137075792844887 | -6.13752760989344 |
|  | chr2 | 202774304 | 202774304 | * | 3.57721088122088e-06 | 0.0277638687256849 | -3.703783226693 |
|  | chr2 | 206180997 | 206180997 | * | 3.27027758526756e-06 | 0.027162457868297 | 6.62466401992454 |
|  | chr2 | 207688274 | 207688274 | * | 2.93847620584896e-07 | 0.0107812291291298 | -3.50413290554136 |
|  | chr2 | 208137962 | 208137962 | * | 7.73824285354079e-06 | 0.036456021313964 | -3.7070980014235 |
|  | chr2 | 218399526 | 218399526 | * | 9.89743353815815e-07 | 0.0175211836284023 | 6.05017134111854 |
|  | chr2 | 218569335 | 218569335 | * | 1.1544762803554e-05 | 0.0412330398472758 | -2.32558139534884 |
|  | chr2 | 218672206 | 218672206 | * | 1.04562510705025e-05 | 0.0402474126567004 | 3.24189526184539 |
|  | chr2 | 222301568 | 222301568 | * | 6.77196306306057e-08 | 0.00632913995781165 | -8.62820727821566 |
|  | chr2 | 232436201 | 232436201 | * | 5.35722639231182e-07 | 0.0139321212339453 | 5.28169014084507 |
|  | chr2 | 236404585 | 236404585 | * | 2.77165770808521e-06 | 0.0261031234884416 | -4.6916030228316 |
|  | chr2 | 238487662 | 238487662 | * | 5.56168345441599e-06 | 0.0323744925409985 | 7.91495818939075 |
|  | chr2 | 239724525 | 239724525 | * | 1.8184812873219e-05 | 0.0495890631775887 | 4.10371360217255 |
|  | chr2 | 240558045 | 240558045 | * | 1.34154677073642e-06 | 0.0190538219820252 | 9.16650674854474 |
|  | chr2 | 240780611 | 240780611 | * | 1.5815874632584e-05 | 0.0471449739477541 | 7.65911976679722 |
|  | chr2 | 241474032 | 241474032 | * | 3.54680925100814e-06 | 0.0277638687256849 | 3.48121460288761 |
|  | chr2 | 241779379 | 241779379 | * | 1.55931398150248e-05 | 0.0469371566071801 | 2.94117647058824 |
|  | chr2 | 241779382 | 241779382 | * | 2.76507434274612e-06 | 0.0261031234884416 | -4 |
|  | chr20 | 609951 | 609951 | * | 1.03025338850801e-05 | 0.0402474126567004 | 5.99516207063377 |
|  | chr20 | 3049119 | 3049119 | * | 1.41514936804818e-07 | 0.00816944669016107 | -5.1490514905149 |
|  | chr20 | 4104670 | 4104670 | * | 7.80752733488604e-06 | 0.0364983966694477 | -4.39586625529886 |
|  | chr20 | 9838624 | 9838624 | * | 1.09806668791967e-05 | 0.0408887682689852 | -5.859375 |
|  | chr20 | 20369368 | 20369368 | * | 1.37851770647647e-05 | 0.0446273176508244 | 2.60303687635575 |
|  | chr20 | 22579072 | 22579072 | * | 1.82444521281585e-05 | 0.0496205507199091 | 5.91928945805685 |
|  | chr20 | 25386540 | 25386540 | * | 5.35019252752125e-06 | 0.0315991635642953 | 4.73171325902918 |
|  | chr20 | 25476601 | 25476601 | * | 1.08638385477829e-05 | 0.040600542752425 | 4.68249861039235 |
|  | chr20 | 31379944 | 31379944 | * | 1.21938918608024e-05 | 0.0424495632726324 | 8.50800462122462 |
|  | chr20 | 33732464 | 33732464 | * | 1.7472152821138e-05 | 0.0485756138841827 | -3.67892976588629 |
|  | chr20 | 35741802 | 35741802 | * | 6.72739477937461e-08 | 0.00632913995781165 | 3 |
|  | chr20 | 37519327 | 37519327 | * | 1.20144853053397e-05 | 0.0421055516129941 | -6.46026328620185 |
|  | chr20 | 38407179 | 38407179 | * | 7.2505669585931e-06 | 0.0356225044397533 | 3.17460317460317 |
|  | chr20 | 47470076 | 47470076 | * | 1.54539814710103e-05 | 0.0467778690068462 | 2.88759763523351 |
|  | chr20 | 47490598 | 47490598 | * | 1.23851225225991e-06 | 0.0190538219820252 | -4.62046204620462 |
|  | chr20 | 49655239 | 49655239 | * | 7.50373243766906e-06 | 0.0360517060018553 | -5.78739102331668 |
|  | chr20 | 51022705 | 51022705 | * | 5.78343103323569e-06 | 0.0328689369263055 | 3.10421286031042 |
|  | chr20 | 53807931 | 53807931 | * | 5.06282096384773e-06 | 0.0313575170167993 | -13.2609130957149 |
|  | chr20 | 56603756 | 56603756 | * | 1.68525973740295e-05 | 0.0480106905549777 | 4.7582833283451 |
|  | chr20 | 57212687 | 57212687 | * | 1.35840960433573e-06 | 0.0190538219820252 | -15.8317507963734 |
|  | chr20 | 60043306 | 60043306 | * | 9.4345415549385e-06 | 0.0390468070056357 | -6.15662242941464 |
|  | chr20 | 62035116 | 62035116 | * | 8.08288145806809e-06 | 0.0367265471261412 | -3.02325581395348 |
|  | chr20 | 62170272 | 62170272 | * | 1.80393188768893e-06 | 0.0221112105859451 | -8.23123807178477 |
|  | chr20 | 62796018 | 62796018 | * | 9.95850335124074e-07 | 0.0175211836284023 | 3.1496062992126 |
|  | chr20 | 63031269 | 63031269 | * | 5.69783837082554e-06 | 0.0327072575386812 | 3.10235249968154 |
|  | chr21 | 26171066 | 26171066 | * | 1.18699816916724e-05 | 0.0418825275394198 | 3.35267569310122 |
|  | chr21 | 28112294 | 28112294 | * | 1.6264352183809e-05 | 0.047590414573974 | 12.4949302717374 |
|  | chr21 | 32521661 | 32521661 | * | 3.36131450582208e-06 | 0.027234173286512 | -2.60691613765452 |
|  | chr21 | 33246133 | 33246133 | * | 1.33852846247437e-07 | 0.00816944669016107 | 3.5781041388518 |
|  | chr21 | 34670772 | 34670772 | * | 1.49006873912849e-05 | 0.0462595851785968 | -7.27213801356203 |
|  | chr21 | 37154797 | 37154797 | * | 9.7198834929306e-06 | 0.0397414035646381 | 2.94117647058824 |
|  | chr21 | 39054217 | 39054217 | * | 1.83534851619697e-06 | 0.0221112105859451 | 5.74818361874729 |
|  | chr21 | 39396482 | 39396482 | * | 9.31824850795603e-07 | 0.0172134685291864 | -3.04568527918782 |
|  | chr21 | 40772770 | 40772770 | * | 9.21132014729348e-09 | 0.0029090210526172 | 5.28266066657291 |
|  | chr21 | 40772860 | 40772860 | * | 5.12150178143637e-06 | 0.0313575170167993 | -5.01792114695341 |
|  | chr21 | 40847077 | 40847077 | * | 3.14340659223398e-06 | 0.0267673661748649 | -7.50392984197899 |
|  | chr21 | 41426026 | 41426026 | * | 9.38322840323998e-07 | 0.0172134685291864 | -4.41413596297568 |
|  | chr21 | 43802530 | 43802530 | * | 1.42128061395756e-05 | 0.0450629053763874 | 2.97242864087784 |
|  | chr21 | 43897825 | 43897825 | * | 1.296969831219e-05 | 0.0434993442155344 | 3.00429184549357 |
|  | chr21 | 44294953 | 44294953 | * | 1.59081332816273e-05 | 0.0471449739477541 | -8.70753289150839 |
|  | chr21 | 44455543 | 44455543 | * | 1.62727065097455e-05 | 0.047590414573974 | -3.2345013477089 |
|  | chr21 | 44751723 | 44751723 | * | 6.78809594240408e-06 | 0.0348253914311561 | -6.51162790697675 |
|  | chr21 | 46137299 | 46137299 | * | 1.51962049215177e-05 | 0.046434456775118 | 5.87546544545193 |
|  | chr21 | 46344218 | 46344218 | * | 2.58619001275929e-07 | 0.0104484958144424 | 5.79710144927537 |
|  | chr22 | 11057580 | 11057580 | * | 7.4608696459023e-07 | 0.0158605756232505 | -5.17581163779691 |
|  | chr22 | 19692927 | 19692927 | * | 2.42301985717332e-06 | 0.0250744346058153 | 4.17069409923594 |
|  | chr22 | 19786862 | 19786862 | * | 4.74652425650443e-06 | 0.0313575170167993 | -3.1578947368421 |
|  | chr22 | 19981528 | 19981528 | * | 1.26324769719523e-05 | 0.0429270569962312 | -8.12830586221276 |
|  | chr22 | 21458070 | 21458070 | * | 9.28612401604674e-06 | 0.0389716415506095 | 6.99588477366255 |
|  | chr22 | 21973237 | 21973237 | * | 4.24799076588882e-06 | 0.0300778986178758 | 4.93421052631579 |
|  | chr22 | 29693745 | 29693745 | * | 1.04491361585048e-05 | 0.0402474126567004 | -4.15130324221233 |
|  | chr22 | 30246489 | 30246489 | * | 2.93400027498114e-06 | 0.0262052441014524 | -2.74223034734918 |
|  | chr22 | 32469080 | 32469080 | * | 1.01806772173446e-06 | 0.0177358456710746 | 8.91292490164448 |
|  | chr22 | 32847421 | 32847421 | * | 1.56422880326514e-05 | 0.0469371566071801 | 4.20643409577094 |
|  | chr22 | 35257586 | 35257586 | * | 9.16791654294357e-06 | 0.0387730207552552 | 9.38481991781484 |
|  | chr22 | 37257394 | 37257394 | * | 1.62139007958298e-05 | 0.0475774355698065 | -5.27472527472528 |
|  | chr22 | 38061256 | 38061256 | * | 4.26975079129608e-06 | 0.0301438306221516 | 3.82022471910113 |
|  | chr22 | 38282988 | 38282988 | * | 1.55188823825674e-07 | 0.00816944669016107 | -10.1271289672432 |
|  | chr22 | 38355313 | 38355313 | * | 8.19635112915716e-06 | 0.0367265471261412 | -4.97452139141513 |
|  | chr22 | 38411441 | 38411441 | * | 5.84979607778193e-06 | 0.0328689369263055 | -4 |
|  | chr22 | 38872450 | 38872450 | * | 8.62392087210798e-06 | 0.037585439420415 | 10.6687216371265 |
|  | chr22 | 39510209 | 39510209 | * | 1.59831408400313e-05 | 0.0471449739477541 | -4.65016366612112 |
|  | chr22 | 40001961 | 40001961 | * | 1.82964095253076e-05 | 0.0496205507199091 | -9.72614126036266 |
|  | chr22 | 45027694 | 45027694 | * | 1.72669937845869e-05 | 0.048365839378543 | 5.68681318681319 |
|  | chr22 | 45930791 | 45930791 | * | 9.01363982591285e-06 | 0.0384844115216733 | -6.50038802590233 |
|  | chr22 | 48545249 | 48545249 | * | 5.0132377396395e-06 | 0.0313575170167993 | -7.46580624213645 |
|  | chr22 | 49775690 | 49775690 | * | 1.66432782402167e-05 | 0.0479530277492557 | 4.10686223153484 |
|  | chr3 | 5919212 | 5919212 | * | 2.31821905964116e-06 | 0.0245137088109691 | -4.13093211227789 |
|  | chr3 | 9844018 | 9844018 | * | 9.94671653898148e-06 | 0.0399440836606242 | 3.14769975786925 |
|  | chr3 | 10211849 | 10211849 | * | 1.33932961767404e-05 | 0.0440217640066932 | -4.63681838313836 |
|  | chr3 | 11480121 | 11480121 | * | 1.79560167986735e-05 | 0.0492048294438072 | 4.34782608695652 |
|  | chr3 | 12768963 | 12768963 | * | 1.26033308448717e-05 | 0.0429270569962312 | -12.1813362438606 |
|  | chr3 | 14810552 | 14810552 | * | 9.08598431690872e-06 | 0.0385427631373967 | -3.77358490566038 |
|  | chr3 | 15465099 | 15465099 | * | 1.50718106355024e-05 | 0.0463189237354633 | 2.37724642165986 |
|  | chr3 | 25851970 | 25851970 | * | 5.62764811725361e-07 | 0.0139321212339453 | -4.80225988700564 |
|  | chr3 | 33219143 | 33219143 | * | 7.3655725161254e-06 | 0.0357736833443673 | -4.24481555572748 |
|  | chr3 | 35942708 | 35942708 | * | 1.29018648414381e-07 | 0.00816944669016107 | 10.3641456582633 |
|  | chr3 | 36945830 | 36945830 | * | 7.09938436805248e-06 | 0.0354461429446485 | 2.17391304347826 |
|  | chr3 | 38025286 | 38025286 | * | 1.97803346963104e-07 | 0.00903748936933798 | 9.36225895316804 |
|  | chr3 | 48269413 | 48269413 | * | 1.76602616972716e-06 | 0.0218188118204366 | 9.36724565756824 |
|  | chr3 | 48640203 | 48640203 | * | 8.28534371263196e-07 | 0.0163328485347103 | 5.01567398119123 |
|  | chr3 | 48940470 | 48940470 | * | 3.30958659829064e-07 | 0.0116148790506454 | 5.5045871559633 |
|  | chr3 | 49347314 | 49347314 | * | 3.76944845853265e-06 | 0.0280920309052914 | -7.36590382494703 |
|  | chr3 | 49657354 | 49657354 | * | 1.29103114922295e-05 | 0.0433602212178779 | 5.36912751677853 |
|  | chr3 | 75306684 | 75306684 | * | 3.67493362190298e-07 | 0.0117501795376771 | 5.53935860058309 |
|  | chr3 | 85206603 | 85206603 | * | 1.63532412844349e-06 | 0.021114620328688 | 4.12362611689777 |
|  | chr3 | 122179671 | 122179671 | * | 2.01210474686905e-06 | 0.0226622047432119 | 8.9059002175989 |
|  | chr3 | 126593623 | 126593623 | * | 1.90765689320357e-06 | 0.0224263216453233 | -4.74868528257166 |
|  | chr3 | 127034602 | 127034602 | * | 2.20218953245774e-07 | 0.0098753152235591 | -2.92249047013977 |
|  | chr3 | 128617568 | 128617568 | * | 2.44414870172704e-06 | 0.025078736651872 | 3.7885109114249 |
|  | chr3 | 129161581 | 129161581 | * | 1.07159560339821e-05 | 0.0405187716477507 | 3.16301703163017 |
|  | chr3 | 129230020 | 129230020 | * | 1.29034763856002e-05 | 0.0433602212178779 | -6.01712144556784 |
|  | chr3 | 129278792 | 129278792 | * | 6.42332091581329e-06 | 0.034035603327331 | 9.4451508778794 |
|  | chr3 | 129398949 | 129398949 | * | 4.23627557438222e-07 | 0.0129851552252017 | 4.03022670025189 |
|  | chr3 | 133463166 | 133463166 | * | 1.59553418420496e-05 | 0.0471449739477541 | -9.0045772610091 |
|  | chr3 | 133677552 | 133677552 | * | 4.83615905674836e-07 | 0.0137075792844887 | -5.3639846743295 |
|  | chr3 | 136796252 | 136796252 | * | 2.06813181455647e-06 | 0.0230785497743537 | -3.0741410488246 |
|  | chr3 | 141402158 | 141402158 | * | 1.31867636557795e-06 | 0.0190538219820252 | 4.65116279069767 |
|  | chr3 | 141737312 | 141737312 | * | 1.28717864627731e-06 | 0.0190538219820252 | 19.7775220560031 |
|  | chr3 | 141737342 | 141737342 | * | 7.54121239601965e-08 | 0.00632913995781165 | 21.5074798619102 |
|  | chr3 | 143943388 | 143943388 | * | 1.19290025422139e-05 | 0.0419861183939677 | -5.06688963210702 |
|  | chr3 | 153131522 | 153131522 | * | 5.6510762864524e-06 | 0.0326593231766333 | 3.20469690108042 |
|  | chr3 | 155080060 | 155080060 | * | 3.65183544227445e-07 | 0.0117501795376771 | 4 |
|  | chr3 | 156445158 | 156445158 | * | 1.32103741675468e-05 | 0.0437480588205542 | 2.78422273781903 |
|  | chr3 | 171067442 | 171067442 | * | 4.35057059622884e-06 | 0.0303582860999448 | -5.59088262952846 |
|  | chr3 | 181075703 | 181075703 | * | 1.32533230201344e-06 | 0.0190538219820252 | 5.24861878453039 |
|  | chr3 | 184074592 | 184074592 | * | 3.30467915374721e-06 | 0.0272325936393854 | -5.48233139770097 |
|  | chr3 | 185175718 | 185175718 | * | 4.92609424179556e-06 | 0.0313575170167993 | -18.0898897367488 |
|  | chr3 | 185175790 | 185175790 | * | 2.11494406789755e-06 | 0.023273252720545 | -19.9308755760369 |
|  | chr3 | 185175799 | 185175799 | * | 1.90781049630858e-06 | 0.0224263216453233 | -20.6832217018974 |
|  | chr3 | 185175895 | 185175895 | * | 1.24877332157633e-08 | 0.0029090210526172 | -20.3564589777581 |
|  | chr3 | 186567173 | 186567173 | * | 2.96328117578107e-06 | 0.0262732141249285 | -2.64084507042254 |
|  | chr3 | 187392884 | 187392884 | * | 7.69797242991089e-06 | 0.036456021313964 | 4.35967302452316 |
|  | chr3 | 189381717 | 189381717 | * | 1.62289732324964e-05 | 0.0475774355698065 | -6.43360844954621 |
|  | chr3 | 190529414 | 190529414 | * | 1.43968241874949e-05 | 0.0451834658383908 | -4.00946311968261 |
|  | chr3 | 191329299 | 191329299 | * | 7.41818061435977e-06 | 0.0358886380697614 | 2.57352941176471 |
|  | chr3 | 195732639 | 195732639 | * | 4.95204999178977e-06 | 0.0313575170167993 | -3.89221556886228 |
|  | chr3 | 196131581 | 196131581 | * | 2.65145460408153e-06 | 0.025994249064276 | 4.26136363636363 |
|  | chr4 | 1022188 | 1022188 | * | 1.55962722181127e-05 | 0.0469371566071801 | -6.15253789315223 |
|  | chr4 | 1739124 | 1739124 | * | 1.79182259236447e-05 | 0.0491944892733664 | 2.50415767024084 |
|  | chr4 | 5731783 | 5731783 | * | 1.12629969404482e-05 | 0.0410799299966808 | -12.1774545943463 |
|  | chr4 | 6641453 | 6641453 | * | 7.33297721147725e-06 | 0.0357736833443673 | -9.89877029689517 |
|  | chr4 | 8260107 | 8260107 | * | 9.98510174232551e-07 | 0.0175211836284023 | -9.96790095150751 |
|  | chr4 | 9054152 | 9054152 | * | 1.35346248149959e-05 | 0.0441560802478204 | -3.85874802873664 |
|  | chr4 | 17546576 | 17546576 | * | 4.741611753718e-07 | 0.0137075792844887 | 5.9007070197888 |
|  | chr4 | 21632361 | 21632361 | * | 7.65814523244511e-06 | 0.0363616393781726 | -9.44688575849784 |
|  | chr4 | 40806120 | 40806120 | * | 1.4478808940192e-07 | 0.00816944669016107 | -16.1822502513481 |
|  | chr4 | 41085124 | 41085124 | * | 1.48041175560735e-06 | 0.0201784722939121 | -5.55555555555556 |
|  | chr4 | 54231010 | 54231010 | * | 1.67797538234882e-05 | 0.0480106905549777 | 10.0642938185486 |
|  | chr4 | 56213361 | 56213361 | * | 4.31725270821291e-06 | 0.0303024964283232 | 3.88699596091794 |
|  | chr4 | 58218843 | 58218843 | * | 1.39794329615041e-05 | 0.0448427563112155 | -5.06100996755202 |
|  | chr4 | 67451360 | 67451360 | * | 1.82774355551001e-05 | 0.0496205507199091 | 2.85714285714286 |
|  | chr4 | 74933774 | 74933774 | * | 5.59965729121427e-07 | 0.0139321212339453 | 9.71060811698262 |
|  | chr4 | 82935535 | 82935535 | * | 1.08646768113113e-05 | 0.040600542752425 | -2.35756385068763 |
|  | chr4 | 84325253 | 84325253 | * | 1.70874722690134e-06 | 0.0215509603182585 | 6.66344435327045 |
|  | chr4 | 95942157 | 95942157 | * | 5.72029022574664e-06 | 0.0327467142095567 | -5.63933729821581 |
|  | chr4 | 100608839 | 100608839 | * | 1.35965980573009e-07 | 0.00816944669016107 | 4.01606425702812 |
|  | chr4 | 112407847 | 112407847 | * | 3.22074528134587e-06 | 0.0268935673858026 | 6.99570907072562 |
|  | chr4 | 128746837 | 128746837 | * | 6.68419731856234e-06 | 0.0345492461630478 | -6.21260056042664 |
|  | chr4 | 131615842 | 131615842 | * | 2.50321823783772e-06 | 0.0255764580704194 | -11.1706288473965 |
|  | chr4 | 139087708 | 139087708 | * | 1.50396221952125e-05 | 0.0463189237354633 | -4.65838509316771 |
|  | chr4 | 143514235 | 143514235 | * | 9.22701162542489e-06 | 0.0388582516318726 | -4.08805031446541 |
|  | chr4 | 144173623 | 144173623 | * | 4.52911846427565e-07 | 0.0134619944368297 | 5.53347814975722 |
|  | chr4 | 147617936 | 147617936 | * | 2.7080919902305e-06 | 0.0261031234884416 | 10.233918128655 |
|  | chr4 | 160832876 | 160832876 | * | 1.74654397369897e-05 | 0.0485756138841827 | -4.67515865406565 |
|  | chr4 | 177568269 | 177568269 | * | 8.22410460745599e-06 | 0.0367265471261412 | -3.84615384615384 |
|  | chr4 | 184290618 | 184290618 | * | 1.63334719028829e-07 | 0.00841532096818298 | 4.5548585418508 |
|  | chr4 | 188220882 | 188220882 | * | 3.74240184669757e-06 | 0.0280920309052914 | 6.51585991932526 |
|  | chr5 | 1424093 | 1424093 | * | 4.33074839040418e-06 | 0.0303093684409359 | 6.77322677322678 |
|  | chr5 | 2608686 | 2608686 | * | 1.0248145231695e-05 | 0.0402474126567004 | 8.21229418353229 |
|  | chr5 | 2839320 | 2839320 | * | 6.24766483737638e-06 | 0.0337698975029394 | 9.86182767004685 |
|  | chr5 | 15555427 | 15555427 | * | 1.72523099355322e-05 | 0.048365839378543 | -6.44376899696049 |
|  | chr5 | 17141887 | 17141887 | * | 1.440642631228e-05 | 0.0451834658383908 | -2.94117647058824 |
|  | chr5 | 27063535 | 27063535 | * | 1.59885158947429e-05 | 0.0471449739477541 | 7.6494605250235 |
|  | chr5 | 27120101 | 27120101 | * | 1.39871882061887e-05 | 0.0448427563112155 | 3.43964043654446 |
|  | chr5 | 52372525 | 52372525 | * | 2.89960362276628e-07 | 0.0107812291291298 | -5.71428571428572 |
|  | chr5 | 55988715 | 55988715 | * | 1.25457732798963e-07 | 0.00816944669016107 | 8.36857589408844 |
|  | chr5 | 57219233 | 57219233 | * | 2.29118076684971e-07 | 0.010087573188237 | 7.53597141951906 |
|  | chr5 | 64769449 | 64769449 | * | 8.08932460490612e-06 | 0.0367265471261412 | -3.43137254901961 |
|  | chr5 | 65448742 | 65448742 | * | 1.3485190925006e-05 | 0.0440685666205409 | -4.25452202190951 |
|  | chr5 | 72420960 | 72420960 | * | 6.71477181284763e-06 | 0.034595804473908 | -4.36046511627907 |
|  | chr5 | 72946355 | 72946355 | * | 5.03300503907113e-06 | 0.0313575170167993 | 5.39909848817265 |
|  | chr5 | 79585734 | 79585734 | * | 7.55088837211655e-06 | 0.0360517060018553 | 5.64914488865336 |
|  | chr5 | 79622165 | 79622165 | * | 6.09637450311972e-06 | 0.033300446843227 | 3.94736842105263 |
|  | chr5 | 80112498 | 80112498 | * | 1.0863050277283e-05 | 0.040600542752425 | -4.91505183737719 |
|  | chr5 | 82013122 | 82013122 | * | 7.31905877908652e-06 | 0.0357736833443673 | -2.9623424403196 |
|  | chr5 | 90765970 | 90765970 | * | 5.69989097035035e-06 | 0.0327072575386812 | 4.22535211267606 |
|  | chr5 | 110894313 | 110894313 | * | 1.68975933275867e-06 | 0.021423060768662 | -16.3926681783825 |
|  | chr5 | 116078052 | 116078052 | * | 5.20013478258187e-06 | 0.0313575170167993 | -3.84615384615384 |
|  | chr5 | 122758561 | 122758561 | * | 1.39998718873634e-05 | 0.0448427563112155 | -2.92792792792793 |
|  | chr5 | 126663676 | 126663676 | * | 6.96334884442245e-06 | 0.0349833300634505 | 5.69199817795323 |
|  | chr5 | 128965515 | 128965515 | * | 9.09946714415888e-07 | 0.0170316828996343 | 4.57317073170732 |
|  | chr5 | 138057155 | 138057155 | * | 4.94908943626472e-06 | 0.0313575170167993 | -9.07636945221911 |
|  | chr5 | 139372773 | 139372773 | * | 4.66617137765147e-06 | 0.0313575170167993 | 4.79626362821365 |
|  | chr5 | 139795462 | 139795462 | * | 1.64127908471886e-05 | 0.0478268132767551 | 12.7010032160515 |
|  | chr5 | 149581740 | 149581740 | * | 3.37400263963319e-06 | 0.027234173286512 | 4.89702105600034 |
|  | chr5 | 158037442 | 158037442 | * | 3.78932169518143e-06 | 0.0280920309052914 | -3.87983763345195 |
|  | chr5 | 171159749 | 171159749 | * | 5.53915514460005e-07 | 0.0139321212339453 | 7.50870855373501 |
|  | chr5 | 173031166 | 173031166 | * | 1.51522046793024e-05 | 0.046434456775118 | 3.38028169014084 |
|  | chr5 | 173502211 | 173502211 | * | 8.23230110978445e-06 | 0.0367265471261412 | 3.93430502746093 |
|  | chr5 | 176292832 | 176292832 | * | 3.04471537973899e-06 | 0.0263316881364812 | -2.68562401263823 |
|  | chr5 | 177313461 | 177313461 | * | 3.80424074596685e-06 | 0.0280920309052914 | -6.56063841744408 |
|  | chr5 | 177479857 | 177479857 | * | 1.42006284544735e-06 | 0.0196498640125655 | -7.52689735388193 |
|  | chr5 | 178319664 | 178319664 | * | 1.24389026559539e-05 | 0.0425789195721407 | 7.14124293785311 |
|  | chr5 | 178971620 | 178971620 | * | 1.04183203745016e-05 | 0.0402474126567004 | 5.27735840595525 |
|  | chr5 | 180137214 | 180137214 | * | 1.31403178594142e-06 | 0.0190538219820252 | 3.98770754014454 |
|  | chr5 | 180297205 | 180297205 | * | 1.24350910293486e-05 | 0.0425789195721407 | 3.20869508024204 |
|  | chr6 | 6826565 | 6826565 | * | 6.40389617553987e-06 | 0.034035603327331 | 10.407960199005 |
|  | chr6 | 10421288 | 10421288 | * | 5.30944655325793e-06 | 0.0315050203832809 | -4.31585496917246 |
|  | chr6 | 13631099 | 13631099 | * | 1.7297680425387e-05 | 0.048365839378543 | -2.82608695652173 |
|  | chr6 | 13772581 | 13772581 | * | 8.18727818680242e-06 | 0.0367265471261412 | -4.63768115942029 |
|  | chr6 | 13902194 | 13902194 | * | 2.60005703291728e-06 | 0.0258271394599251 | -3.49693737459077 |
|  | chr6 | 17511650 | 17511650 | * | 5.74385680739047e-07 | 0.014049421533997 | -6.78823601645484 |
|  | chr6 | 21267044 | 21267044 | * | 9.08843114189062e-06 | 0.0385427631373967 | -3.37288845455815 |
|  | chr6 | 21571801 | 21571801 | * | 1.52762369053012e-05 | 0.0465306682132464 | 4.48687398228682 |
|  | chr6 | 23364947 | 23364947 | * | 1.16248490745542e-05 | 0.041396959418168 | -5.35294117647058 |
|  | chr6 | 27640220 | 27640220 | * | 1.14904165436845e-05 | 0.0412111122709942 | -5.08446802699856 |
|  | chr6 | 28281877 | 28281877 | * | 4.75344743641367e-06 | 0.0313575170167993 | -2.88065843621399 |
|  | chr6 | 28461107 | 28461107 | * | 1.18048750239715e-06 | 0.0190538219820252 | -8.64339551449003 |
|  | chr6 | 28642312 | 28642312 | * | 1.73640968011667e-06 | 0.0217471292191704 | 4.59540067498626 |
|  | chr6 | 30102495 | 30102495 | * | 7.25239308883445e-06 | 0.0356225044397533 | -14.0286352292132 |
|  | chr6 | 31725517 | 31725517 | * | 2.77398317639341e-06 | 0.0261031234884416 | 8.33031983285117 |
|  | chr6 | 36567602 | 36567602 | * | 8.13121031542391e-07 | 0.0163328485347103 | 5.8028185979282 |
|  | chr6 | 41896051 | 41896051 | * | 1.50467878531266e-05 | 0.0463189237354633 | 6.85714285714286 |
|  | chr6 | 43470638 | 43470638 | * | 4.79446781483946e-06 | 0.0313575170167993 | 3.92156862745098 |
|  | chr6 | 50843125 | 50843125 | * | 1.34526897920997e-05 | 0.0440217640066932 | -7.75567541319089 |
|  | chr6 | 103695681 | 103695681 | * | 2.58889829973907e-07 | 0.0104484958144424 | -11.2293537301851 |
|  | chr6 | 106026394 | 106026394 | * | 1.06037743983863e-05 | 0.0402474126567004 | 3.5264483627204 |
|  | chr6 | 122825809 | 122825809 | * | 5.7879394018354e-06 | 0.0328689369263055 | -6.74597063956472 |
|  | chr6 | 136068591 | 136068591 | * | 4.86701632068498e-07 | 0.0137075792844887 | -2.75229357798165 |
|  | chr6 | 138242174 | 138242174 | * | 6.5980902878361e-06 | 0.0342703714790988 | -4.34541533073429 |
|  | chr6 | 145636377 | 145636377 | * | 4.89595473175967e-07 | 0.0137075792844887 | -2.97339593114241 |
|  | chr6 | 149741599 | 149741599 | * | 1.19556320075455e-05 | 0.0420187714526322 | 4.53967320665964 |
|  | chr6 | 150842862 | 150842862 | * | 1.10896740720072e-05 | 0.0408887682689852 | -3.34261838440112 |
|  | chr6 | 151477940 | 151477940 | * | 5.2810209011692e-06 | 0.0314205302796785 | -2.07336523125997 |
|  | chr6 | 151670968 | 151670968 | * | 1.4275625049842e-05 | 0.0450629053763874 | -4.04411764705882 |
|  | chr6 | 154731514 | 154731514 | * | 1.01372316002767e-05 | 0.0402474126567004 | 3.48027842227379 |
|  | chr6 | 155248192 | 155248192 | * | 1.22272032293097e-06 | 0.0190538219820252 | -3.92158081153297 |
|  | chr6 | 157274827 | 157274827 | * | 4.01248004161811e-06 | 0.0290040143512226 | -3 |
|  | chr6 | 163714603 | 163714603 | * | 1.59981769044961e-06 | 0.0209405844960656 | -4.96559633027523 |
|  | chr6 | 166782569 | 166782569 | * | 6.38838449873361e-06 | 0.034035603327331 | -10.4849848684721 |
|  | chr6 | 169988847 | 169988847 | * | 1.34076105122376e-05 | 0.0440217640066932 | -7 |
|  | chr7 | 226462 | 226462 | * | 2.84950239951122e-06 | 0.0261031234884416 | -12.7575778887404 |
|  | chr7 | 261962 | 261962 | * | 1.0188741318228e-05 | 0.0402474126567004 | 2.53411306042886 |
|  | chr7 | 346289 | 346289 | * | 4.8938669164941e-06 | 0.0313575170167993 | -3.14606741573034 |
|  | chr7 | 868312 | 868312 | * | 2.89146892593403e-06 | 0.0261571757711947 | 3.82978723404256 |
|  | chr7 | 1499867 | 1499867 | * | 1.20171672815594e-05 | 0.0421055516129941 | 14.9506479554153 |
|  | chr7 | 1563587 | 1563587 | * | 9.22134230258398e-06 | 0.0388582516318726 | -4.81927710843374 |
|  | chr7 | 1817621 | 1817621 | * | 1.94882006123356e-06 | 0.0226622047432119 | 9.02777777777778 |
|  | chr7 | 2247755 | 2247755 | * | 3.31862277593911e-06 | 0.0272325936393854 | -6.92970822281167 |
|  | chr7 | 2525942 | 2525942 | * | 2.94778574507238e-08 | 0.00475876970870058 | -18.302111210229 |
|  | chr7 | 2526825 | 2526825 | * | 1.01369161400933e-05 | 0.0402474126567004 | 3.6923076923077 |
|  | chr7 | 2625858 | 2625858 | * | 1.71005204502355e-05 | 0.048319066961936 | -5.26947837942773 |
|  | chr7 | 2626944 | 2626944 | * | 1.67481306686356e-06 | 0.021423060768662 | 3.24636532801345 |
|  | chr7 | 4972779 | 4972779 | * | 7.70844990782583e-06 | 0.036456021313964 | 4.01002506265664 |
|  | chr7 | 5429762 | 5429762 | * | 1.96655518986887e-07 | 0.00903748936933798 | 5.94795539033457 |
|  | chr7 | 5568635 | 5568635 | * | 1.11630676206037e-05 | 0.0408951804816762 | 2.93453724604966 |
|  | chr7 | 20952773 | 20952773 | * | 1.23667789642854e-05 | 0.0425789195721407 | 4.15430267062314 |
|  | chr7 | 23367925 | 23367925 | * | 9.50058595084955e-06 | 0.0391924419048495 | 4.89725542674061 |
|  | chr7 | 23502297 | 23502297 | * | 1.27226746192117e-05 | 0.0430284231750481 | 4.34662940379404 |
|  | chr7 | 33028852 | 33028852 | * | 6.0030364066937e-07 | 0.0145365387529374 | -6.15942028985508 |
|  | chr7 | 34321336 | 34321336 | * | 7.57971129737913e-08 | 0.00632913995781165 | -10.4835732742709 |
|  | chr7 | 39031710 | 39031710 | * | 1.15356860245811e-05 | 0.0412330398472758 | 11.4967984582867 |
|  | chr7 | 39603434 | 39603434 | * | 1.37091722334355e-05 | 0.0445002487233289 | -3.96883429202821 |
|  | chr7 | 57235206 | 57235206 | * | 1.1396725785386e-05 | 0.0410799299966808 | 8.00653594771241 |
|  | chr7 | 64889547 | 64889547 | * | 2.81824147767907e-06 | 0.0261031234884416 | -7.57267068788501 |
|  | chr7 | 67291064 | 67291064 | * | 1.05545652046925e-05 | 0.0402474126567004 | 2.73348519362187 |
|  | chr7 | 72481097 | 72481097 | * | 4.36084244825277e-08 | 0.0058666195414222 | 5.0125313283208 |
|  | chr7 | 73607045 | 73607045 | * | 4.55861826183533e-07 | 0.0134619944368297 | 11.3077593722755 |
|  | chr7 | 73768768 | 73768768 | * | 9.26924266197624e-06 | 0.0389683306466979 | 14.2284959622421 |
|  | chr7 | 77340630 | 77340630 | * | 1.16062039429369e-05 | 0.0413914324597112 | 4.26229508196722 |
|  | chr7 | 90596770 | 90596770 | * | 1.33711051168839e-05 | 0.0440217640066932 | 2.68041237113402 |
|  | chr7 | 95552580 | 95552580 | * | 8.55403020277162e-06 | 0.037585439420415 | 5.87345748636071 |
|  | chr7 | 97034460 | 97034460 | * | 2.37775522556613e-07 | 0.0102817999805721 | 5.5921052631579 |
|  | chr7 | 99355175 | 99355175 | * | 5.63823994310755e-06 | 0.0326593231766333 | 4.47275684467335 |
|  | chr7 | 99392838 | 99392838 | * | 9.97942499144923e-06 | 0.0400090844022666 | 13.573883161512 |
|  | chr7 | 100018678 | 100018678 | * | 8.17146908830771e-06 | 0.0367265471261412 | -5.4671159656054 |
|  | chr7 | 100660692 | 100660692 | * | 1.65383067722429e-05 | 0.0479246852482212 | 6.03506787330317 |
|  | chr7 | 101205783 | 101205783 | * | 5.43027253522758e-07 | 0.0139321212339453 | 4.6875 |
|  | chr7 | 101363889 | 101363889 | * | 2.82894260773789e-06 | 0.0261031234884416 | 10.3257778860996 |
|  | chr7 | 103989582 | 103989582 | * | 6.60915077310145e-06 | 0.0342703714790988 | -3.27380952380952 |
|  | chr7 | 117323133 | 117323133 | * | 2.91651746693417e-06 | 0.0261571757711947 | -4.18250950570342 |
|  | chr7 | 117535825 | 117535825 | * | 1.35484400671024e-05 | 0.0441560802478204 | -4.07253906173992 |
|  | chr7 | 122300438 | 122300438 | * | 1.05502788364223e-05 | 0.0402474126567004 | 10.1644736842105 |
|  | chr7 | 127251716 | 127251716 | * | 5.07805220854235e-06 | 0.0313575170167993 | 10.721318729948 |
|  | chr7 | 138700635 | 138700635 | * | 1.29203305320105e-08 | 0.0029090210526172 | 6.9951421566388 |
|  | chr7 | 140078163 | 140078163 | * | 6.69146769150154e-06 | 0.0345492461630478 | -4.30673642903859 |
|  | chr7 | 140393969 | 140393969 | * | 2.02415432021201e-06 | 0.0226923723850801 | -5.7210164315266 |
|  | chr7 | 143379998 | 143379998 | * | 8.79682126970516e-07 | 0.0166420120359769 | -11.9819939097048 |
|  | chr7 | 143468437 | 143468437 | * | 1.10236288052571e-05 | 0.0408887682689852 | 4.93479292704794 |
|  | chr7 | 146906074 | 146906074 | * | 1.34491804613436e-05 | 0.0440217640066932 | 8.59518410538819 |
|  | chr7 | 148993478 | 148993478 | * | 1.73168060789628e-05 | 0.048365839378543 | -3.6697247706422 |
|  | chr7 | 149421900 | 149421900 | * | 5.0803423588224e-06 | 0.0313575170167993 | 9.96917970602181 |
|  | chr7 | 152676305 | 152676305 | * | 1.14172769672771e-05 | 0.0410799299966808 | 2.77777777777778 |
|  | chr7 | 155360499 | 155360499 | * | 3.02641332177058e-06 | 0.0262732141249285 | -9.61845172371488 |
|  | chr7 | 155457235 | 155457235 | * | 1.05378819791469e-05 | 0.0402474126567004 | 5.34173110501372 |
|  | chr8 | 949899 | 949899 | * | 1.62108761943337e-05 | 0.0475774355698065 | -4.70588235294118 |
|  | chr8 | 1247088 | 1247088 | * | 1.30232528203337e-06 | 0.0190538219820252 | -12.451703547594 |
|  | chr8 | 5672393 | 5672393 | * | 1.33278733650977e-06 | 0.0190538219820252 | -3.98009950248757 |
|  | chr8 | 12921672 | 12921672 | * | 3.02261043768104e-06 | 0.0262732141249285 | -6.54931275620931 |
|  | chr8 | 29303376 | 29303376 | * | 5.17391497758656e-06 | 0.0313575170167993 | 5.56142986996123 |
|  | chr8 | 30750423 | 30750423 | * | 5.55668637627258e-06 | 0.0323744925409985 | 4.46272950839243 |
|  | chr8 | 40519426 | 40519426 | * | 7.16446950246069e-07 | 0.0157541361209436 | 6.18102421595872 |
|  | chr8 | 41767111 | 41767111 | * | 1.34184082341507e-05 | 0.0440217640066932 | 3.49891764173234 |
|  | chr8 | 42690828 | 42690828 | * | 1.0328927801209e-06 | 0.0178655849052782 | -6.38092408260288 |
|  | chr8 | 48403659 | 48403659 | * | 3.20397063165275e-06 | 0.0268460699226184 | -5.26184029966993 |
|  | chr8 | 55229356 | 55229356 | * | 6.43718126614293e-08 | 0.00632913995781165 | 5.4320987654321 |
|  | chr8 | 55977099 | 55977099 | * | 1.17008167916795e-05 | 0.0416028401796149 | 3.05927342256214 |
|  | chr8 | 61132393 | 61132393 | * | 4.4851964818974e-06 | 0.0309431405185342 | -5.15231746165692 |
|  | chr8 | 63039418 | 63039418 | * | 1.67567748969994e-05 | 0.0480106905549777 | -4.21052631578947 |
|  | chr8 | 67064253 | 67064253 | * | 1.70474707385095e-05 | 0.0482254425991749 | -3.17460317460317 |
|  | chr8 | 73767122 | 73767122 | * | 3.67142813711528e-06 | 0.0280456689220722 | 5.48233649087694 |
|  | chr8 | 80873302 | 80873302 | * | 4.30549316380994e-06 | 0.0303024964283232 | 4.38871473354232 |
|  | chr8 | 94723366 | 94723366 | * | 2.53103641970704e-06 | 0.0256442809725925 | -8.10091547796466 |
|  | chr8 | 100224350 | 100224350 | * | 8.43105484438887e-07 | 0.0163328485347103 | 3.34029227557411 |
|  | chr8 | 121624983 | 121624983 | * | 1.57650842442788e-05 | 0.0471449739477541 | -5.16317957418972 |
|  | chr8 | 122542120 | 122542120 | * | 3.76143781964883e-06 | 0.0280920309052914 | -4.21686746987952 |
|  | chr8 | 134760095 | 134760095 | * | 7.55061407887537e-06 | 0.0360517060018553 | -4.90754198966409 |
|  | chr8 | 138496780 | 138496780 | * | 1.96347429738915e-06 | 0.0226622047432119 | -7.14688525910858 |
|  | chr8 | 140200571 | 140200571 | * | 1.41101363641215e-05 | 0.0450555048141686 | 2.79636564552765 |
|  | chr8 | 141154068 | 141154068 | * | 3.54372199963189e-06 | 0.0277638687256849 | 5.31977910361133 |
|  | chr8 | 142834808 | 142834808 | * | 5.95006298328293e-06 | 0.0330464723990186 | 5.31993004659111 |
|  | chr8 | 142940667 | 142940667 | * | 9.14346657941798e-07 | 0.0170316828996343 | -11.8873323502563 |
|  | chr8 | 143381688 | 143381688 | * | 1.93928158698498e-07 | 0.00903748936933798 | 6.99588477366255 |
|  | chr8 | 143498599 | 143498599 | * | 1.44180217331032e-05 | 0.0451834658383908 | -4.00470884150801 |
|  | chr8 | 144266859 | 144266859 | * | 2.01163495116898e-06 | 0.0226622047432119 | 7.53289789645567 |
|  | chr8 | 144277336 | 144277336 | * | 1.90634935996776e-06 | 0.0224263216453233 | -5.50372208436725 |
|  | chr9 | 871426 | 871426 | * | 1.60445244765348e-05 | 0.0472081572298759 | 2.60223048327137 |
|  | chr9 | 2095327 | 2095327 | * | 1.6003581447049e-05 | 0.0471449739477541 | 3.2258064516129 |
|  | chr9 | 3441971 | 3441971 | * | 6.84832269406648e-06 | 0.0349100275189403 | 4.72375690607735 |
|  | chr9 | 6529949 | 6529949 | * | 3.16184463446396e-06 | 0.0267709958025809 | -6.5264423076923 |
|  | chr9 | 24223375 | 24223375 | * | 6.11955776954168e-06 | 0.033300446843227 | -7.39347058932212 |
|  | chr9 | 42328585 | 42328585 | * | 1.24490973095433e-05 | 0.0425789195721407 | -2.30416816385196 |
|  | chr9 | 75909145 | 75909145 | * | 1.25641428165982e-06 | 0.0190538219820252 | 4.97076023391813 |
|  | chr9 | 77020934 | 77020934 | * | 6.23112634873035e-07 | 0.0145628956625912 | -4.3859649122807 |
|  | chr9 | 77380531 | 77380531 | * | 1.13576225719322e-05 | 0.0410799299966808 | 4.08567027830891 |
|  | chr9 | 78237223 | 78237223 | * | 1.12685113379828e-08 | 0.0029090210526172 | 5.38672282508019 |
|  | chr9 | 97077607 | 97077607 | * | 7.44523984648551e-06 | 0.0359141017743026 | 13.9398906318145 |
|  | chr9 | 97854388 | 97854388 | * | 1.13369414588314e-05 | 0.0410799299966808 | 8.22414299346693 |
|  | chr9 | 98255710 | 98255710 | * | 3.53099352560251e-06 | 0.0277638687256849 | -3.27510917030568 |
|  | chr9 | 99204645 | 99204645 | * | 2.40636225902177e-06 | 0.0250744346058153 | -3.75073127925117 |
|  | chr9 | 103389890 | 103389890 | * | 1.68366574849017e-05 | 0.0480106905549777 | 5.57693023717623 |
|  | chr9 | 120580967 | 120580967 | * | 1.81624556364492e-05 | 0.0495839338892745 | -8.97815126050421 |
|  | chr9 | 126515516 | 126515516 | * | 8.53761816626588e-06 | 0.037585439420415 | 9.81593203644423 |
|  | chr9 | 126619069 | 126619069 | * | 4.87999839751813e-06 | 0.0313575170167993 | -4.47761194029851 |
|  | chr9 | 127785405 | 127785405 | * | 7.57798201011853e-06 | 0.0360517060018553 | -2.4390243902439 |
|  | chr9 | 127983532 | 127983532 | * | 1.53153649647255e-05 | 0.0465327867483021 | 3.0961549664431 |
|  | chr9 | 129055710 | 129055710 | * | 7.57447162940646e-06 | 0.0360517060018553 | -3.8235294117647 |
|  | chr9 | 130355118 | 130355118 | * | 7.1429424720936e-06 | 0.0354684397187285 | -6.9781768821134 |
|  | chr9 | 130433571 | 130433571 | * | 8.20087232196028e-06 | 0.0367265471261412 | -3.05343511450382 |
|  | chr9 | 130666469 | 130666469 | * | 2.58084972690541e-06 | 0.0258271394599251 | 7.13938546556144 |
|  | chr9 | 130918909 | 130918909 | * | 1.03643721743917e-05 | 0.0402474126567004 | -7.17668691146153 |
|  | chr9 | 133116163 | 133116163 | * | 1.71597193909503e-05 | 0.048365839378543 | -10.2272727272727 |
|  | chr9 | 133348445 | 133348445 | * | 6.00726834241919e-06 | 0.0332118413618417 | 8.32371367964087 |
|  | chr9 | 133376595 | 133376595 | * | 5.81326498931851e-06 | 0.0328689369263055 | 5.12820512820513 |
|  | chr9 | 134136074 | 134136074 | * | 9.02699397377545e-06 | 0.0384844115216733 | 4.62633451957295 |
|  | chr9 | 134324904 | 134324904 | * | 1.17722671003965e-06 | 0.0190538219820252 | 16.6843920666858 |
|  | chr9 | 136171634 | 136171634 | * | 6.5977828159003e-06 | 0.0342703714790988 | 14.0753851523159 |
|  | chr9 | 136370647 | 136370647 | * | 8.96546826005828e-06 | 0.0384844115216733 | -4.0590405904059 |
|  | chr9 | 136371063 | 136371063 | * | 1.02554388295039e-05 | 0.0402474126567004 | 5.09416195856874 |
|  | chr9 | 136397335 | 136397335 | * | 3.39554499204956e-06 | 0.0273170015287135 | -7.17336313391202 |
|  | chr9 | 136554810 | 136554810 | * | 4.55388660179903e-06 | 0.0311197644277593 | -7.64874281317358 |
|  | chr9 | 136714856 | 136714856 | * | 3.44718018308453e-06 | 0.0275705546452178 | -15.5861637941396 |
|  | chr9 | 137162431 | 137162431 | * | 4.39295111763726e-07 | 0.0132970841410541 | -21.3974040320353 |
|  | chr9 | 137199360 | 137199360 | * | 1.03107906018935e-05 | 0.0402474126567004 | 4.80419963178583 |
|  | chrX | 398301 | 398301 | * | 1.68268214308046e-05 | 0.0480106905549777 | -3.09197260575812 |
|  | chrX | 414532 | 414532 | * | 8.50781463645002e-06 | 0.037585439420415 | 5.35271180432472 |
|  | chrX | 478689 | 478689 | * | 1.31149057498679e-05 | 0.0437061148818485 | 4.25924695935519 |
|  | chrX | 1089526 | 1089526 | * | 4.72706866418603e-06 | 0.0313575170167993 | -10.8288159561618 |
|  | chrX | 1696066 | 1696066 | * | 9.44915795226347e-06 | 0.0390468070056357 | -3.89972144846796 |
|  | chrX | 2201089 | 2201089 | * | 1.69630836579074e-08 | 0.00315974099486277 | 5.08849557522124 |
|  | chrX | 2488670 | 2488670 | * | 1.72309847421424e-05 | 0.048365839378543 | -6.14025661415462 |
|  | chrX | 15382048 | 15382048 | * | 1.03705420622926e-05 | 0.0402474126567004 | 7.46147285745453 |
|  | chrX | 22739030 | 22739030 | * | 3.12228730318918e-06 | 0.0267163091716573 | -9.50068712780577 |
|  | chrX | 23121065 | 23121065 | * | 4.507097469653e-06 | 0.0310058984170065 | -5.9242671009772 |
|  | chrX | 23859640 | 23859640 | * | 1.79626315865151e-05 | 0.0492048294438072 | -7.99878345498783 |
|  | chrX | 27142747 | 27142747 | * | 5.64826945659072e-09 | 0.00273549191709752 | -15.2111046847889 |
|  | chrX | 48468264 | 48468264 | * | 9.83972611004655e-06 | 0.0397414035646381 | -11.109943977591 |
|  | chrX | 51639919 | 51639919 | * | 6.5837437040591e-06 | 0.0342703714790988 | -11.1652353423181 |
|  | chrX | 53499755 | 53499755 | * | 2.58406320262923e-07 | 0.0104484958144424 | 7.99140049140049 |
|  | chrX | 53721470 | 53721470 | * | 8.59110866493183e-06 | 0.037585439420415 | -5.33310995700005 |
|  | chrX | 54373986 | 54373986 | * | 7.9981422628521e-06 | 0.0366425698605044 | -7.9023183197337 |
|  | chrX | 54770121 | 54770121 | * | 1.04881391756922e-05 | 0.0402474126567004 | -9.24320858662614 |
|  | chrX | 55488722 | 55488722 | * | 1.17877225545908e-05 | 0.0417314847738899 | -10.9950681752248 |
|  | chrX | 77624089 | 77624089 | * | 1.50728245492398e-05 | 0.0463189237354633 | 7.23748295343853 |
|  | chrX | 94040148 | 94040148 | * | 1.78122809981075e-05 | 0.0491802621659287 | -5.96432964329644 |
|  | chrX | 97318732 | 97318732 | * | 1.20931903760079e-06 | 0.0190538219820252 | 6.22084871376656 |
|  | chrX | 100846021 | 100846021 | * | 1.54749831975432e-06 | 0.0208184175207388 | -7.82350077766037 |
|  | chrX | 129740705 | 129740705 | * | 7.86493017070898e-06 | 0.0365550330541403 | 3.87096774193548 |
|  | chrX | 144717246 | 144717246 | * | 1.05009018773622e-05 | 0.0402474126567004 | -14.0084159087824 |
|  | chrX | 147912219 | 147912219 | * | 7.57087054682713e-07 | 0.0159418241096773 | 14.1434661495606 |
|  | chrX | 153769168 | 153769168 | * | 1.04207357904666e-06 | 0.0178965494747691 | -4.73815461346634 |
